# Optical control of gene expression using a DNA G-quadruplex targeting reversible photoswitch

**DOI:** 10.1038/s41557-025-01792-1

**Published:** 2025-04-03

**Authors:** Xiaoyun Zhang, Somdutta Dhir, Larry Melidis, Yuqi Chen, Zutao Yu, Angela Simeone, Jochen Spiegel, Santosh Adhikari, Shankar Balasubramanian

**Affiliations:** 1 Yusuf Hamied Department of Chemistry, University of Cambridge, Cambridge, UK; 2 Cancer Research UK Cambridge Institute, Li Ka Shing Centre, University of Cambridge, Cambridge, UK; 3 School of Clinical Medicine, University of Cambridge, Cambridge, UK

**Keywords:** Chemical tools, DNA, Nucleic acids, Small molecules, Target identification

## Abstract

Transcriptional regulation is a dynamic process that coordinates diverse cellular activities, and the use of small molecules to perturb gene expression has propelled our understanding of the fundamental regulatory mechanisms. However, small molecules typically lack the spatiotemporal precision required in highly non-invasive, controlled settings. Here we present the development of a cell-permeable small-molecule DNA G-quadruplex (G4) binder, termed G4switch, that can be reversibly toggled on and off by visible light. We have biophysically characterized the light-mediated control of G4 binding in vitro, followed by cellular, genomic mapping of G4switch to G4 targets in chromatin to confirm G4-selective, light-dependent binding in a cellular context. By deploying G4switch in living cells, we show spatiotemporal control over the expression of a set of G4-containing genes and G4-associated cell proliferation. Our studies demonstrate a chemical tool and approach to interrogate the dynamics of key biological processes directly at the molecular level in cells.

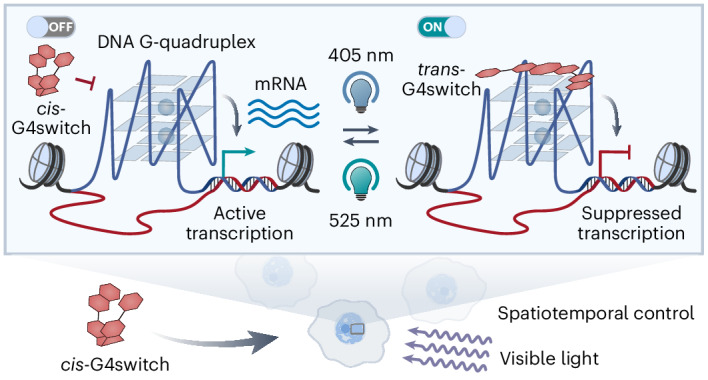

## Abstract (web summary)

Dynamic gene expression is crucial for coordinating diverse cellular activities, but challenging to modulate with high spatiotemporal precision. Now, a photoswitchable DNA structure-specific, small-molecule ligand enables reversible, light-controlled regulation of gene expression and cell proliferation in live cells.

## Main

The dynamic gene expression control is vital for regulating critical biological processes such as cell differentiation and development, and cellular homeostasis^1^. During these processes, genes are expressed at specific times and locations within cell populations^2,3^. Methods that enable spatiotemporal modulation of gene expression will help us elucidate gene regulatory mechanisms. Small molecules can be used to interrogate gene regulation^4^; however, they generally lack the high resolution for spatiotemporal control necessary in highly non-invasive and controlled applications. Photomodulation of biomolecular targets can enable spatiotemporal control with high precision, as exemplified by light-inducible transcriptional effectors^5^. Photocontrolled bioactive small molecules offer an approach that simplifies dosing and circumvents the genetic engineering required by the conventional optogenetics technique^5–7^. For example, photoswitchable inhibitors of histone deacetylase have provided optical control of the epigenetically regulated transcriptome^8^. Herein we introduce an approach for reversible, spatiotemporal control of gene expression by manipulating DNA with light-responsive small molecules.

**Fig. 1:**
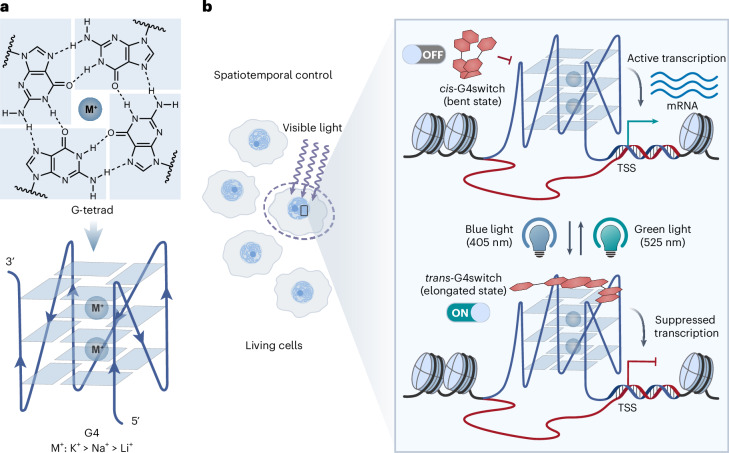
Schematic for the optical control of gene expression by G4switch. **a**, A G-tetrad, comprising four Hoogsteen hydrogen-bonded guanines, stabilized by a central monovalent cation (top) following a preference order of potassium (K^+^) > sodium (Na^+^) > lithium (Li^+^), and a parallel intramolecular G4 structure composed of stacked G-tetrads (bottom). **b**, Schematic diagram for the concept of optically controlling gene expression using a photoswitchable G4 ligand G4switch. Upon blue (405 nm) or green (525 nm) light illumination, G4switch can be switched ‘ON’ or ‘OFF’, respectively, in live cells to enable it to reversibly bind to endogenous DNA G4s and inhibit the gene expression, thus achieving spatiotemporal control of gene expression. TSS, transcription start site.

DNA G-quadruplexes (G4s) are four-stranded secondary nucleic acid structures formed by stacked G-tetrads, stabilized by centrally coordinated cations (M^+^) in a preference order of K^+^ > Na^+^ > Li^+^, within certain guanine-rich genomic sequences^9,10^ (Fig. 1a). DNA G4 structures have previously been identified in human cells^11,12^, and the dynamics of their formation have been visualized in live cells^13^. Recently, endogenous G4-forming sites have been mapped in human chromatin, showing their predominant localization in regulatory regions, particularly within active promoters of cancer genes^14,15^. These G4s have been shown to be highly correlated with transcriptional programmes across different breast cancer subtypes^16^. Furthermore, it has been demonstrated that G4 formation within gene promoters at specific loci, through site-specific G4 editing in human genome, can promote downstream gene transcription^17,18^. Notably, accumulating evidence suggests that G4s can operate as binding hubs for transcription factors and the associated proteins^19–21^. However, the exact timing of G4 formation and how this is precisely linked to transcriptional regulation are still not fully understood^22^. Thus, understanding the mechanisms of G4-associated transcriptional regulation has become a significant challenge central to the field, underscoring the need for the development of tools to target folded G4s with high spatiotemporal precision. Several G4-selective small-molecule ligands have been shown to perturb gene expression^23,24^, but they lack the precision for highly non-invasive spatiotemporal control. Attempts at photoresponsive G4 ligands have shown limited applicability in biological contexts^25–29^, owing to a number of challenges that include lack of reversibility or insufficient difference in activity between two isomers, loss of efficacy in physiological conditions (for example, high K^+^ concentration), the low tissue penetration and the potential for causing cell phototoxicity upon ultraviolet (UV) light exposure^30^.

Herein we present a chemical approach to optically modulate transcription of G4-containing genes in situ. We developed a cell-permeable small-molecule G4 ligand that is photoswitchable, designated G4switch. Its elongated *trans* conformation exhibits considerably higher G4-binding affinity and selectivity, compared with the bent *cis* form, which we demonstrate in vitro and in cells. Upon illumination with low-intensity blue (405 nm) and green (525 nm) light, G4switch can undergo rapid *cis*-to-*trans* and *trans*-to-*cis* photoisomerizations, respectively. We demonstrate that activated G4switch predominantly localizes to promoter G4s in human, cellular chromatin and can modulate transcription. This enables the optical control of expression at hundreds of G4-containing genes and G4-associated cell proliferation with a high spatial and temporal resolution. Here we demonstrate that a DNA-targeting small molecule can be exploited for the reversible optical control of gene expression.

## Results

### Design and characterization of G4switch

**Fig. 2:**
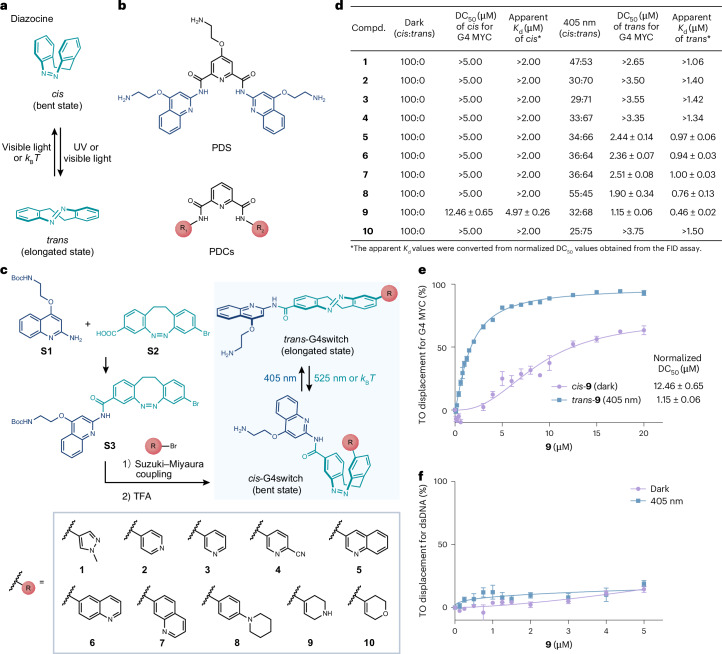
Design, synthesis and screening of the G4switch. **a**, Diazocine photoisomerization. **b**, Chemical structures of PDS and PDC scaffold. R_1_ and R_2_ represent *N*-heterocycles. **c**, Synthesis and photoswitching of the putative G4switches. TFA, trifluoroacetic acid. **d**, Summary of *cis*:*trans* ratios of putative G4switches in the dark and following 405 nm illumination in DMSO, as determined by ^1^H NMR spectroscopy at room temperature (Supplementary Fig. 1), and their normalized DC_50_ values, the concentrations of the corresponding isomers required to achieve a 50% decrease in fluorescence upon competitive displacement of a fluorescent G4 binder TO from a G4 MYC structure, obtained from the FID assay, as well as the apparent *K*_d_ values derived from the normalized DC_50_ values and the association rate constant (*K*_a_) of TO with G4 MYC (Methods). The FID results are shown as mean ± s.d. from three or four technical replicates (*n* = 3 for **9**; *n* = 4 for the other molecules). **e**,**f**, FID curves showing the percentage of displacement of TO for increasing concentrations of **9** from G4 MYC (**e**) (*n* = 3) and dsDNA (**f**) (*n* = 4) before and after 405 nm illumination. Source data

We set out to design and construct a photoswitchable small molecule with differential binding to G4 DNA in the two conformational states. We reasoned that such a ligand would, in its ‘active’ state, selectively target DNA G4 structures and inhibit gene transcription in living cells, enabling spatiotemporal control of G4-containing gene expression (Fig. 1b). We selected diazocine as the photoswitching moiety, owing to its well-characterized photothermal properties, a large geometrical difference between its bent *cis* and elongated *trans* isomers, and visible light-controlled photoisomerization in both directions^31–33^ (Fig. 2a). We designed our G4switch probes by tethering a key G4-binding moiety 4-(2-aminoethoxy)quinolin-2-amine (blue), derived from a widely used small-molecule G4 ligand pyridostatin (PDS)^34^, and a lipophilic positively charged nitrogen heterocycle group (red), derived from a potent G4 ligand of the pyridine-2,6-dicarboxamides (PDCs)^35,36^, to a diazocine core (teal) for reversible photoswitching (Fig. 2b,c). Given that small-molecule G4 ligands typically necessitate a flat aromatic skeleton for *π*-stacking with G-tetrads^37^, our design rationale was that such ligands could much more efficiently stack on the G-tetrad of G4s in the elongated *trans* form, which is closer to a planar conformation, compared with the bent *cis* form.

**Extended Data Fig. 1:**
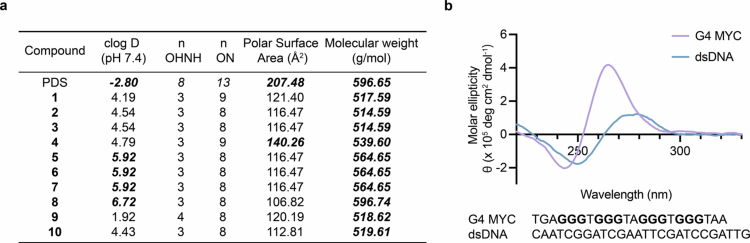
Assessment of the putative G4switches. **a**, Calculated physicochemical properties of PDS and the putative G4switches. Values highlighted in bold italics indicate violation of Lipinski’s rule of five: clog D, partition coefficient (≤ 5); n OHNH, number of hydrogen bond donors (≤ 5); n ON, number of hydrogen bond acceptors (≤ 10); Polar surface area (80 – 140 Å^2^); Molecular weight (≤ 500 Da). **b**, Structure verification of 5 μM G4 MYC (G-runs are highlighted in bold) and dsDNA control used in the FID assay by CD. Mean of three scans (n = 3) is shown. Source data

To identify a suitable photoswitchable ligand of this structural class, we synthesized a series of putative G4switches (**1**–**10**) by amide and Suzuki–Miyaura coupling, in two to three steps from known building blocks **S1** and **S2** (Fig. 2c and Supplementary Information). Given the importance of cell permeability of probes for explorations in living systems, we first calculated the theoretical physiochemical properties of all compounds and assessed them with the ‘Lipinski’s rule of five’ (Extended Data Fig. 1a), which is widely used as a guideline for drug-likeness^38^. Compared with the parent molecule PDS, we observed considerable improvements for all compounds, which obey most of the properties in the rule. Furthermore, we determined the photoswitching properties of all compounds using ^1^H NMR spectroscopy (Fig. 2d and Supplementary Fig. 1). As expected, the selected 3,3′-disubstituted diazocine moiety enabled rapid and reversible photoisomerization for all molecules in dimethyl sulfoxide (DMSO) controlled by visible light (405 nm and 525 nm)^33^: without illumination, we exclusively obtained the thermodynamically more stable *cis* isomers, which could be switched to the *trans* state by 405 nm illumination, reaching the photoisomerization ratios of up to 75% of the *trans* isomer (Fig. 2d and Supplementary Fig. 1).

We then assessed the G4-binding affinity and selectivity of putative G4switches towards G4 structures before and after 405 nm illumination using a well-established fluorescent intercalator displacement (FID) assay^39,40^. Specifically, we evaluated the ability of the putative ligand to competitively displace a known fluorescent G4 binder, thiazole orange (TO), from a folded DNA G4 MYC structure (Extended Data Fig. 1b), by measuring a decrease in fluorescence (DC_50_)^39^ (Fig. 2d,e and Supplementary Fig. 2), which were converted to the binding constant (*K*_d_) of ligands as previously reported^40^ (Fig. 2d and Methods). Compounds **5**–**9** all show a clear difference in apparent G4-binding affinity between their *cis* and *trans* isomers, with at least 2-fold selectivity, while others showed negligible G4 binding in either state (Fig. 2d,e and Supplementary Fig. 2). Compound **9**, where R is 1,2,3,6-tetrahydropyridine, showed the largest change in G4-binding affinity, with the lowest apparent *K*_d_ = 0.46 ± 0.02 µM for the 405 nm illuminated *trans* isomer, and about 10-fold lower G4-binding affinity (apparent *K*_d_ = 4.97 ± 0.26 µM) for the non-illuminated *cis* isomer (Fig. 2d,e). In addition, both isomers of **9** showed no apparent binding to a double-stranded DNA (dsDNA) control (Fig. 2f and Extended Data Fig. 1b). On substituting the basic secondary amine group, compound **10** is incapable of binding to G4s, for either isomer (Fig. 2d and Supplementary Fig. 2i), therefore suggesting that the electrostatic interactions between the positively charged secondary amine group of **9** and the negatively charged phosphate backbone of G4 DNA are critical^37^. Overall, our assay reveals that compound **9** is the most potent photoswitchable G4 ligand, exhibiting the highest G4 selectivity between its *cis* and *trans* forms.

**Extended Data Fig. 2:**
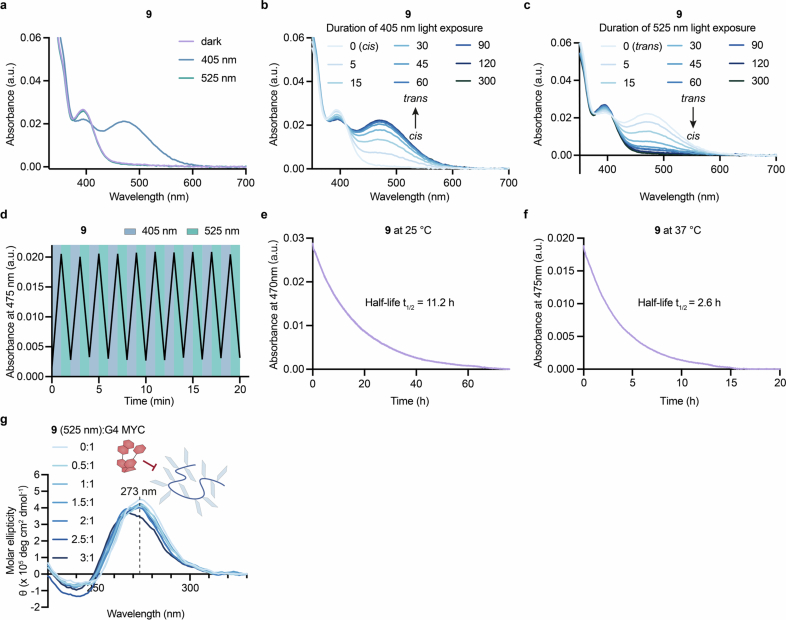
Photophysical characterization of 9 and its interactions with G4 DNA. **a-c**, UV-Vis absorption spectra of 50 μM **9** in PBS, measured (**a**) in the dark, after 405 nm illumination for 2 min and subsequent 525 nm illumination for 2 min, and (**b,c**) after a series of 405 nm and 525 nm illumination durations (0-300 s), starting from the steady dark-adapted state and 405 nm illuminated state, respectively. **d**, UV-Vis absorbance of 50 μM **9** at 475 nm in PBS, measured during consecutive *cis*-*trans* photoisomerization cycles, alternating between 405 nm and 525 nm illuminations. **e**,**f**, Thermal *trans*-to-*cis* relaxation of 50 μM **9** in PBS, with half-lives of (**e**) t_1/2_ = 11.2 h at 25 °C and (**f**) t_1/2_ = 2.6 h at 37 °C, measured by recording UV-Vis absorbance of *trans*-**9** at 470 nm and 475 nm, respectively. **g**, CD spectra of G4 MYC recorded by a titration with increasing amounts of **9** in 10 mM Tris-HCl buffer, 100 mM LiCl, pH 7.4 after further 525 nm illumination for 60 s of the 405 nm illuminated samples shown in Fig. 3e. Source data

Next, we pursued a more detailed characterization of **9**. The photoswitching of **9** was demonstrated in phosphate buffered saline (PBS) by UV–visible spectroscopy (Extended Data Fig. 2a–f). The data confirmed that 405 nm and 525 nm are the ideal photoswitching wavelengths, enabled by the 3,3′-disubstituted diazocine^33^ (Extended Data Fig. 2a). The *cis*–*trans* photoisomerization is rapid and fully reversible by establishing its steady states of photoisomerizations under 405 nm and 525 nm illuminations within ~60 s, without observable degradation of the compound after several cycles (Extended Data Fig. 2b–d). In the dark, a spontaneous, thermally driven *trans*-to-*cis* isomerization of **9** generates 100% *cis* isomer, with half-lives (*t*_1/2_) of 11.2 h and 2.6 h at 25 °C and 37 °C, respectively (Extended Data Fig. 2e,f). This relatively fast reversal mechanism largely confines the bioactivity of activated **9** to 405 nm illuminated sites, as it reverts to its inactivated *cis* form at physiological temperatures within a few hours and would do so when it diffuses into non-illuminated regions.

**Fig. 3:**
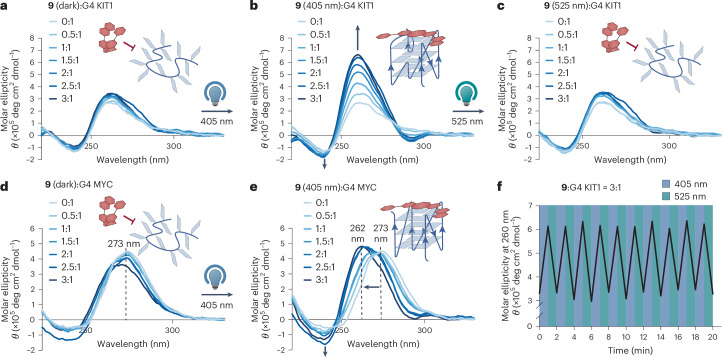
Characterization of light-dependent G4-binding activity of G4switch in vitro. **a**–**e**, CD spectra of 10 μM G4 KIT1 (**a**–**c**) and G4 MYC (**d**,**e**) collected by titrating with increasing amounts of **9** in 10 mM Tris-HCl buffer, 100 mM LiCl, pH 7.4 before (**a**,**d**) and after (**b**,**e**) 405 nm illumination for 60 s, and further (**c**) 525 nm illumination for 60 s. **f**, G4 KIT1 (10 μM) formation, indicated by the CD intensity measured at 260 nm, in the presence of 3 molar equivalents of **9**, is rapidly and reversibly photoswitched in 10 mM Tris-HCl buffer, 100 mM LiCl, pH 7.4, by alternating phases of 405 nm and 525 nm illuminations. Source data

To evaluate the ligand’s capacity to stabilize G4 structure formation, we carried out a titration experiment with **9** monitored by circular dichroism (CD) spectroscopy using two G4 DNA oligonucleotides G4 KIT1 and G4 MYC in the presence of Li^+^ ions, which do not favour G4 formation^41,42^. The CD spectrum of G4 KIT1, in the absence of **9**, exhibits a broad positive band at ~260 nm and a negative band at ~240 nm (Fig. 3a), consistent with the formation of a parallel G4 structure^43^. Upon titration with increasing amounts of **9** in the absence of 405 nm illumination, we observed very little spectral changes for G4 KIT1 (Fig. 3a,c), whereas upon illumination of **9** at 405 nm, we observed a substantial increase in ellipticity at the characteristic G4 bands (Fig. 3b and Supplementary Fig. 3a). For G4 MYC in Li^+^ buffer, we observed a positive band at ~273 nm and a broad negative band at 240–250 nm in the absence of **9** (Fig. 3d), which is not characteristic of any known G4 topology. In the presence of in situ activated **9**, the positive band shifted from ~273 nm to ~260 nm, with increased negative ellipticity at ~240 nm (Fig. 3e and Supplementary Fig. 3b), suggestive of parallel G4 formation^43^, whereas only a slight shift was observed with at least three molar equivalents of inactivated **9** (30 μM, where there will be some G4 binding given its apparent *K*_d_ = 4.97 ± 0.65 μM measured in K^+^ buffer, as shown in Fig. 2d,e) (Fig. 3d and Extended Data Fig. 2g). Taken together, this demonstrates stabilization of parallel G4 structures by the *trans*-**9**, but not the *cis* isomer. Importantly, G4 stabilization by **9** was shown to be fully reversible after at least 10 complete cycles of photoswitching, without any noticeable efficiency loss (Fig. 3f). Our CD spectroscopy data confirm the robustness of **9** as a photoswitchable G4 ligand.

### Mapping the binding sites of 9 in cells upon light exposure

**Fig. 4:**
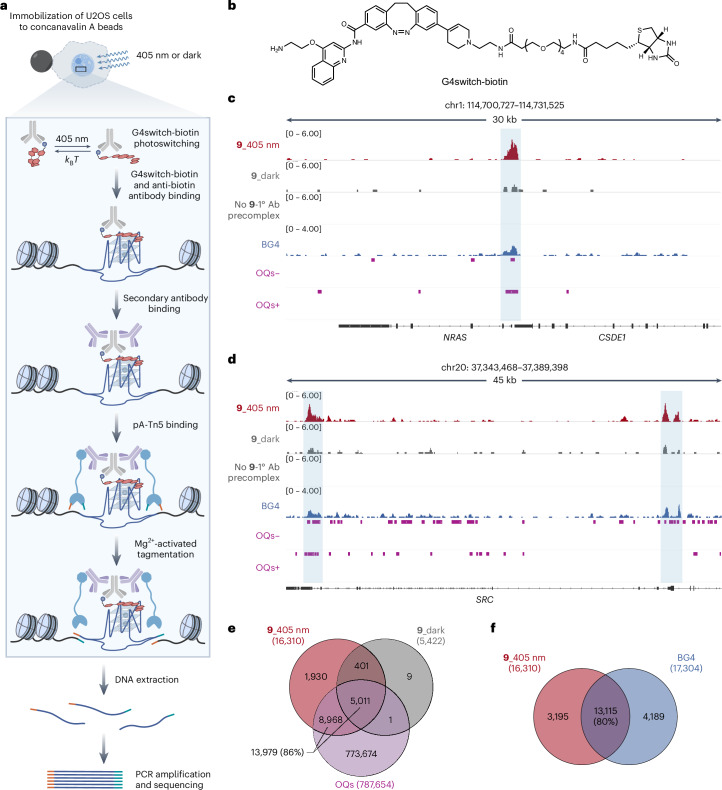
Genome-wide profiling of G4switch binding sites in human cells. **a**, Schematic workflow of the G4switch-biotin Chem-map in U2OS cells. In permeabilized cells with or without 405 nm illumination, a precomplex comprising biotinylated **9** (red orange and blue) and anti-biotin antibody (grey) binds to DNA targets of **9**, followed by a sequential secondary antibody (purple) binding and a fusion transposome pA-Tn5 (light blue) binding to the target sites. Mg^2+^ was then used to activate the Tn5 transposase in situ for DNA cleavage and sequencing adapter (orange and teal) integration adjacent to the **9** binding sites. After DNA isolation, PCR amplification of the fragments labelled with adapters at both ends generated the library, which was sequenced to identify the genomic binding sites of **9**. **b**, Chemical structure of G4switch-biotin. **c**,**d**, Genome browser views of Chem-map binding sites of **9**, highlighted in light blue, in the presence (red) and absence (dark grey) of 405 nm illumination, and the negative control without adding G4switch-biotin-primary antibody precomplex (no **9**-1° Ab), compared with sites mapped by G4-CUT&Tag using G4-selective antibody BG4 (blue) at the *NRAS* (**c**) and *SRC* (**d**) loci, and G4 formation sites identified by G4-seq in both reverse (−) and forward (+) strand (purple). **e**,**f**, Venn diagrams showing the overlap of high-confidence binding sites between *trans*-**9**, *cis*-**9** and OQs (**e**), and between *trans*-**9** and BG4 (**f**). Chem-map experiments for both isomers of **9** were performed in three biological replicates, each with three technical replicates.

Next, we sought to determine whether **9** can bind to folded genomic G4 structures inside cells in a light-dependent manner. We used Chem-map methodology, which enables in situ mapping of binding sites for biotin-tethered chromatin-interacting small molecules^44–46^. This involves the recruitment of a transposase (Tn5) to a biotin tag covalently attached to small molecules, which allows for the site-selective introduction of next-generation sequencing adapters (Fig. 4a).

**Extended Data Fig. 3:**
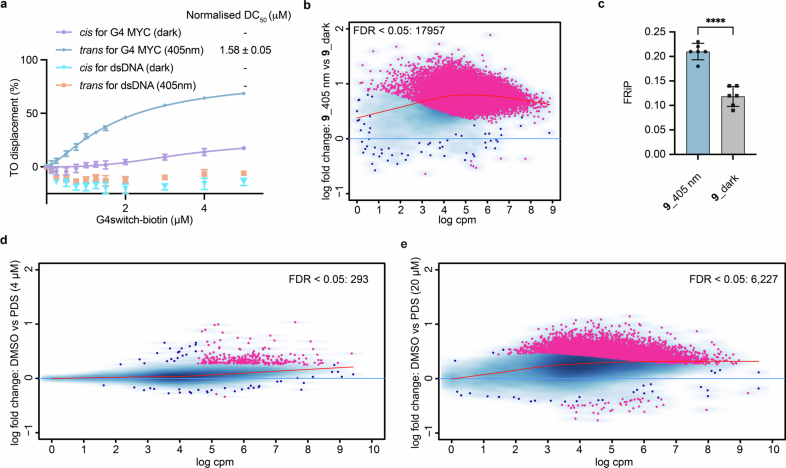
Genome-wide mapping of binding sites of 9 in cells under light illumination. **a**, FID curves for G4 MYC and dsDNA induced by increasing concentrations of non-illuminated and 405 nm illuminated G4switch-biotin. The apparent *K*_d_ of *tran*-G4switch-biotin for G4 MYC is 0.63 ± 0.02 µM, derived from its normalised DC_50_ (1.58 ± 0.05 µM) (See Methods), while *K*_d_ values for other conditions were not determined within the molecule concentration range (0-5 µM). Results are shown as mean ± s.d. from four replicates (*n* = 4). **b**, Differential binding analysis of the union of Chem-map binding sites across all replicates (three biological replicates, each with three technical replicates) for both isomers of **9** shows 17,957 significantly differentially binding sites of *trans*-**9** compared to the *cis* isomer. Dots highlighted in red represent binding sites that are significantly different (FDR < 0.05) between the two isomers. Positive fold changes indicate an increase in *trans*-**9** binding. **c**, FRiP analysis comparing the Chem-map profiles of **9** between the 405 nm illuminated *trans* form and non-illuminated *cis* form in U2OS cells. Results represent the mean ± s.d. from three biological replicates, each with two technical replicates (*n* = 6). ****P < 0.0001, two-tailed unpaired *t*-test. **d**,**e**, Differential binding analysis displaying changes in 405 nm illuminated **9** Chem-map binding events across the union of all three biological replicates for pre-treatment with (**d**) 4 µM and (**e**) 20 µM PDS, compared to the vehicle DMSO for 3 h in U2OS cells. Dots highlighted in red represent significantly changing sites (FDR < 0.05) between DMSO and PDS treatments. A positive fold change indicates a decrease in 405 nm illuminated *trans*-**9** binding. Source data

We first synthesized a biotinylated probe, G4switch-biotin, featuring a long PEG4 spacer to minimize potential steric effects on **9**-target interactions (Fig. 4b). Using the FID assay, we confirmed that G4switch-biotin retains light-dependent G4-binding affinity and selectivity with an apparent *K*_d_ = 0.63 ± 0.02 µM towards G4 MYC only in *trans* state, but not for dsDNA (Extended Data Fig. 3a). To distinguish binding profiles of *cis-* and *trans*-**9**, we performed the Chem-map experiments in permeabilized human osteosarcoma U2OS cells in the presence or absence of a 405 nm light source (Fig. 4a). We identified 16,310 high-confidence binding sites in the genome for *trans*-**9**, whereas a much smaller number of binding sites (5,422) were observed for the *cis*-**9** (Fig. 4c–e), of which nearly all (5,412/5,422) were a subset of *trans*-**9** binding sites (Fig. 4e). This is consistent with the relatively higher binding affinity of *trans*-**9** than the *cis* isomer for G4s (Fig. 2d,e). We carried out an analysis of differential binding sites^47^ for both isomers of **9** and also evaluated the fraction of all mapped sequencing reads that fall into the called binding peak regions (FRiP, a quantitative measure of signal in each binding peak) for binding sites of both isomers of **9**, and the data confirmed substantially stronger and more frequent on-target binding of the *trans* isomer compared with the *cis* form (Extended Data Fig. 3b,c). A comparison of binding sites of *trans*-**9** in the genome with the previously observed universe of possible G-quadruplexes (OQs) detected in purified human genomic DNA by G4 sequencing (G4-seq)^48^ showed high overlap (86%, 13,979/16,310) (Fig. 4e). Furthermore, we mapped 17,306 high-confidence endogenous folded G4 sites in U2OS cells by G4-CUT&Tag using a G4-specific antibody BG4 (ref. ^49^). The majority of *trans*-**9** binding sites (80%, 13,115/16,310) overlapped with endogenous, folded G4s (Fig. 4f). In a competition experiment, we pre-incubated live U2OS cells with a competing G4-binding molecule, PDS (4 μM and 20 μM), for 3 h before mapping the binding of **9** using Chem-map, in the presence of 405 nm illumination. Compared with a DMSO control, we observed substantial dose-dependent competition for *trans*-**9** binding events by PDS (293 and 6,227 significantly changing sites for 4 μM and 20 μM PDS treatment, respectively), as judged by differential binding analysis^47^ (Extended Data Fig. 3d,e). These data suggest that light-activated *trans*-**9** engages at G4 DNA structures in cellular chromatin, while *cis*-**9** shows binding to fewer genomic G4 sites, consistent with its relatively weaker G4-binding affinity.

### Reversible optical modulation of gene expression in live cells

Formation of endogenous G4s in gene promoters has been linked to active transcription^14,17,18^. Treatment with G4-stabilizing molecules can potentially alter gene expression, by interfering with transcription machinery or transcription factor binding^24,50^. We therefore evaluated whether DNA G4 targeting by light-activated **9** could modulate the expression of genes, where G4s are targeted, in living cells.

**Fig. 5:**
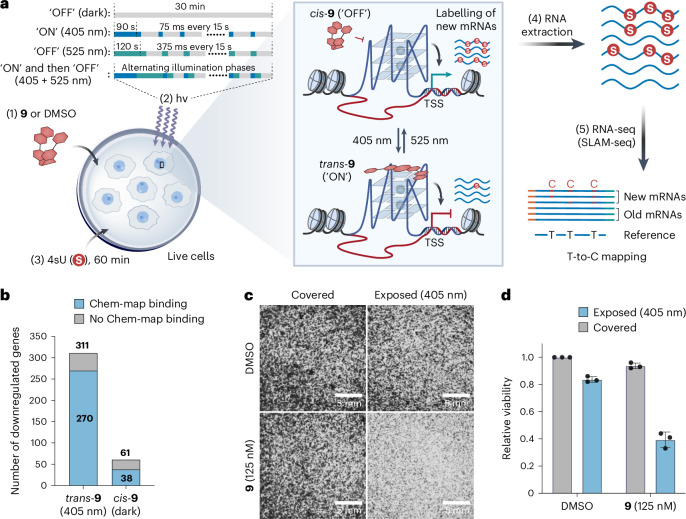
G4switch optically controls transcription in living cells. **a**, Schematic workflow of G4switch-mediated optical control of G4-containing gene expression profiled by SLAM-seq. U2OS cells were treated with **9** or DMSO control for 30 min in the dark or under indicated illumination conditions, followed by 4sU labelling over 60 min. RNA was extracted, and the abundance of newly synthesized RNAs was quantified by analysing T > C conversions through the SLAM-seq protocol. **b**, Stacked bar plot showing the overlap of downregulated genes by *trans*-**9** (upon 405 nm illumination) and *cis*-**9** (in the dark) with the corresponding Chem-map sites. Genes with Chem-map binding sites are shown in blue, while genes without Chem-map binding signals are shown in grey. Four independent biological replicates were performed. **c**, Images showing light-dependent spatial control of U2OS cell proliferation with **9** compared with DMSO control within the same 6-well plate. Half of the plate was covered with a black mask before pulsed 405 nm illumination for 72 h, followed by crystal violet staining and imaging. Representative images from one out of three independent biological replicates with similar results are shown. **d**, Bar plot showing quantification of cell viability relative to DMSO treatment in the dark from **c**. Results are shown as mean ± s.d. from three independent biological replicates (*n* = 3). Source data

**Extended Data Fig. 4:**
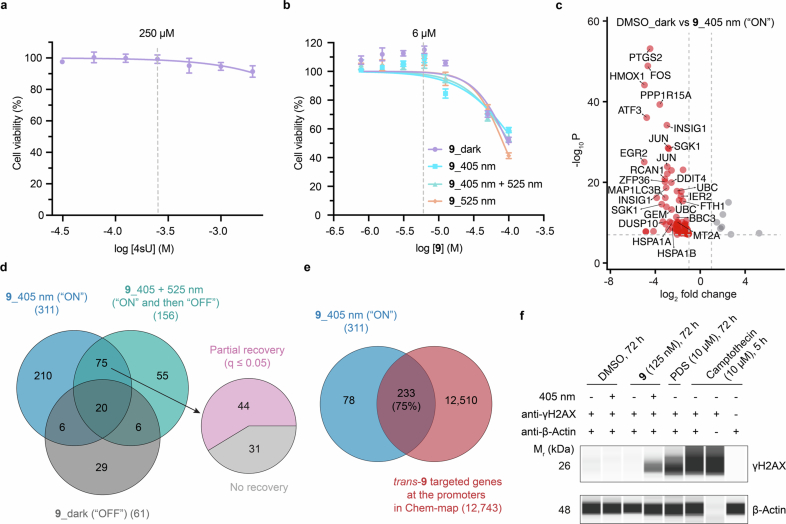
G4switch-mediated optical control of gene expression in U2OS cells. **a**, Cell viability measured by the Cell Titer-Glo luminescent cell viability assay after treatment of U2OS cells with a series of concentrations of 4sU for 60 min. Results represent the mean ± s.d. from four technical replicates (n = 4). **b**, Cell viability after treatment with **9** in the dark and under all illumination conditions used in this study for 30 min, followed by 60 min of 4sU (250 µM) labelling. Results are shown as mean ± s.d. from four technical replicates (n = 4). **c**, Volcano plot displaying the most significantly downregulated genes (fold change >2, q < 0.05, p < 10^-8^), highlighted in red, by *trans*-**9** under pulsed 405 nm illumination compared to the dark-adapted DMSO control. **d**, Venn diagram displaying the overlap of downregulated genes (q ≤ 0.05) by **9** between dark treatment and 405 nm illumination, and the 405 + 525 nm illumination, compared to the DMSO treatment in the dark. The pie chart illustrates that, among the 75 overlapping downregulated genes under the 405 nm and 405 + 525 nm illumination conditions, 44 genes show partial recovery (reduced downregulation, q ≤ 0.05) under the 405 + 525 nm illumination (Supplementary Fig. 6b and Supplementary Table 7), while 31 genes show no recovery compared to the 405 nm condition. **e**, The overlap between downregulated genes (fold change >2, q < 0.05) by *trans*-**9** under 405 nm illumination and genes displaying *trans*-**9** Chem-map binding sites in their promoter regions. **f**, DNA damage marker γH2AX was examined by Western blot after treating cells with compound **9** (125 nM) with or without 405 nm illumination (60 s continuous illumination, followed by 75 ms pulses every 22.5 s) and its parent molecule PDS (10 μM) for 72 h, compared to the dark-adapted DMSO control. Cells treated with Camptothecin, a DNA topoisomerase I inhibitor, for 5 h served as a positive control. A representative image from one of three independent biological replicates with similar results is shown. Source data

**Extended Data Fig. 5:**
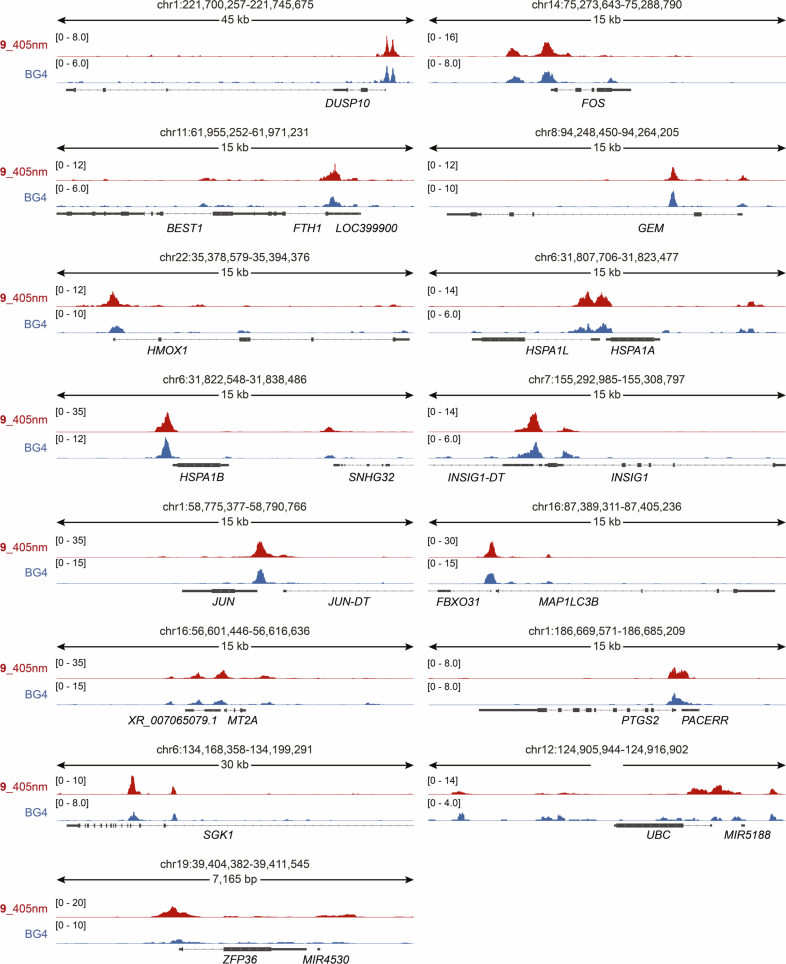
G4 binding of *trans*-9 in the promoters of several of its most significantly downregulated genes. Genome browser views display *trans*-**9** Chem-map binding sites (red), overlapping with naturally occurring G4 sites mapped by G4-CUT&Tag (blue), at the promoter regions of several genes that are most significantly downregulated by *trans*-**9** (fold change >2, q ≤ 0.05, p < 10^-8^), as randomly selected from those highlighted in Extended Data Fig. 4c.

We quantified immediate changes in transcription using a nascent RNA detection method called SLAM-seq^51,52^. We deployed a custom illumination system to light-activate **9** in cultured cells (Supplementary Fig. 4). We subjected U2OS cells to a 30 min treatment with **9** (6 μM) under either 405 nm illumination, control light illuminations or in the dark (Fig. 5a and Extended Data Fig. 4a,b). We then performed SLAM-seq to quantify changes in nascent RNA transcripts compared with an unilluminated DMSO treatment control (Fig. 5a). We observed that in situ light-activated *trans*-**9** significantly downregulated 311 genes (fold change >2, *q* < 0.05) (Fig. 5b, Extended Data Fig. 4c,d, Supplementary Fig. 5 and Supplementary Data 1). Notably, 87% (270/311) of these genes have *trans*-**9** binding sites as judged by Chem-map (Fig. 5b, and Extended Data Figs. 4e and 5). These observations are consistent with the hypothesis that targeting G4s with small molecules can reduce transcription^24,50^. By contrast, inactivated *cis*-**9** affected 80% fewer genes (61 genes), with reduced *cis*-**9** Chem-map binding signals (38/61) (Fig. 5b and Supplementary Data 1). This suggests that *trans*-**9** binding has a substantial direct impact on transcription, whereas the weaker G4-binding *cis*-**9** has a smaller effect. Interestingly, the expression levels of most (68%, 210/311) *trans*-**9** altered genes were ‘rescued’ by cycles of reversal of **9** (Extended Data Fig. 4d, Supplementary Fig. 6a and Supplementary Data 1). These data show potential for **9** to control gene expression under reversible control using light.

Stabilization of endogenous DNA G4 structures by G4-selective small molecules can inhibit gene expression and has shown good activity against cancer cell lines^23,24^. To exemplify spatial control using **9**, we grew U2OS cells in the same 6-well plate in the presence of **9** (125 nM) or vehicle DMSO for 72 h, activated the molecule with pulsed 405 nm illumination (60 s continuous illumination followed by pulses of 75 ms every 22.5 s) of half of the respective wells and quantified differences in cell density (Fig. 5c,d and Supplementary Fig. 7). We observed a clear inhibition of cell proliferation (<40% relative viability) that is conditional on **9** treatment and 405 nm light exposure, while 405 nm light in the absence of **9** or **9** without light showed negligible effects (Fig. 5c,d). Consistent with a previous study^23^, we observed activation of the cellular DNA damage marker γH2AX upon incubation with 405 nm light-activated **9**, its parent molecule PDS, and camptothecin, a DNA topoisomerase I inhibitor (Extended Data Fig. 4f). In the U2OS cell line, after 72 h of treatment with both G4 ligands (activated **9** and PDS), we did not observe apoptosis, as measured by PARP1 cleavage, unlike with camptothecin (Supplementary Fig. 8), indicating that 405 nm light-activated **9** behaves similarly to PDS, but differently from camptothecin, causing antiproliferative effects rather than cell death. These results confirm the spatial, light-mediated control of **9** action on cell antiproliferation, induced by DNA damage response.

## Discussion

We have designed and validated a small-molecule G4-targeting photoswitchable ligand. The G4switch, **9**, is a chemical tool capable of binding and stabilizing DNA G4s in human cells with reversible, spatiotemporal control. Using a variety of biophysics and cellular genomic approaches, we demonstrated that the ‘ON’ and ‘OFF’ conformations have considerably different affinities and stabilization capacities towards G4s. The activated *trans*-**9** maps strongly to endogenous G4 structures in human chromatin, relative to the inactivated *cis* form, and causes inhibition of genes proximal to the mapped binding sites of **9**. Importantly, the activation of G4switch in living cells is fully biocompatible using only visible light and can be easily reversed to the ‘OFF’ *cis* form. Notably, we have shown that **9** can be used to reversibly modulate the expression of several oncogenes, such as *FOS* and *JUN* (Supplementary Fig. 6b). This may provide ways to elucidate role of cancer-related gene regulation mechanisms through G4-mediated control.

Given that the dynamics of G4 formation have been implicated in a wide range of critical biological processes^10^, such as DNA replication, cancer biology and cellular differentiation^53^, we envisage that there is scope to exploit the spatiotemporal control of G4switch molecule **9** to explore such processes in the future.

## Methods

### Chemical synthesis and characterizations

Full synthesis and characterization of molecules, photoisomerization details, UV–visible spectroscopy, cell viability assays, western blotting and general information are described in Supplementary Information. PDS was synthesized as previously reported^34^. Physicochemical properties of the compounds were calculated using ChemAxon MarvinSketch (version 21.4.0, http://www.chemaxon.com).

### FID assay

The protocol was adapted from that previously reported^54^. HPLC-purified DNA oligonucleotides (G4 MYC, 5′-TGA**GGG**T**GGG**TA**GGG**T**GGG**TAA-3′; dsDNA, 5′-CAATCGGATCGAATTCGATCCGATTG-3′)^54,55^ were purchased from Merck and annealed at 5 μM in the assay buffer (10 mM lithium cacodylate, pH 7.3, 100 mM KCl) at 95 °C for 5 min and slowly cooling to room temperature. A mixture of 2.5 μM annealed oligonucleotides and TO (5 µM for G4 MYC, 7.5 µM for dsDNA) was prepared and added to a 96-well plate (HSP9655, Bio-Rad) at 10 μl per well. Ligand solutions (10 μM or 50 μM in assay buffer) were added along the line, followed by adding assay buffer to make ligand concentration series 0–5 µM or 0–20 µM, with the total volume adjusted to 100 μl per well. The plate was sealed, centrifuged (500 × *g*, 1 min) and incubated with orbital shaking (500 rpm, 3 min). End-point fluorescence was measured on a fluorescence plate reader (BMG PHERAstar Plus) using 485 nm/520 nm filters, with gain adjusted at 80% of the fluorescence from a well without ligand. To measure the binding of *trans* isomers, the same plate was illuminated at 405 nm for 2 min, before the incubation and fluorescence measurement. The percentage of displacement was calculated from the fluorescence intensity (*F*) subtracting buffer absorbance, using the following equation: percentage of displacement = 100 − [(*F*/*F*_0_) × 100], where *F*_0_ is the fluorescence from the fluorescent probe bound to DNA without ligand. FID curves were fitted using a one-site specific binding with the Hill slope model in GraphPad Prism 10 (version 10.2.2) to determine DC_50_. Apparent dissociation constants (*K*_d_) were calculated using the following equations with *K*^TO^_a_ = 5.01 × 10^−^^6^ M^−^^1^ for G4 MYC: *K*^ligand^_a_ = (*K*^TO^_a_ × [TO])/DC_50_, *K*^ligand^_d_ = 1/*K*^ligand^_a_ as described previously^40^. Standard deviations (s.d.) were calculated from three or four replicates (*n* = 3 or 4).

### CD titrations

CD spectra were recorded on an Applied Photophysics Chirascan Plus CD spectrometer using a 1 mm path length quartz cuvette at 20 °C, with the following scanning parameters: 220–330 nm or 220–450 nm, 0.5 s response time, 1 nm intervals and 1 nm bandwidth. For titration experiments, HPLC-purified G4 DNA oligonucleotides (G4 MYC, 5′-TGA**GGG**T**GGG**TA**GGG**T**GGG**TAA-3′; G4 KIT1, 5′-A**GGG**A**GGG**CGCT**GGG**AGGA**GGG**-3′)^55,56^ were purchased from Merck and annealed in 10 mM Tris-HCl (pH 7.4) with 100 mM LiCl. Indicated amounts of **9** were added to make a final concentration of 10 μM oligonucleotides. After a 2 min equilibration, the same sample was measured before and after 1 min of 405 nm illumination, followed by another measurement after 1 min of 525 nm illumination. Reported spectra, plotted using GraphPad Prism 10 (version 10.2.2), represent a smoothed average of three scans, with baseline correction against the buffer and zero adjustment at 330 nm. Molar ellipticity (*θ*) is quoted in ×10^5^ deg cm^2^ dmol^−^^1^.

### Cell culture

Human osteosarcoma U2OS cells (ATCC, HTB-96) were cultured in phenol red-free Dulbecco’s modified Eagle medium (DMEM) (high glucose and l-glutamine plus, Gibco, catalogue number 21063045) supplemented with 10% (v/v) heat-inactivated fetal bovine serum (Gibco, catalogue number 10082147) and 1 mM sodium pyruvate (hereafter referred to as full-growth DMEM media). Cells were maintained at 37 °C in a humidified 5% CO_2_ atmosphere. After thawing, cells were passaged at least twice before use in experiments. Periodic tests confirmed that the cells were mycoplasma-free.

### G4switch Chem-map

The protocol was adapted from that previously described^46^. U2OS cells were grown to a 70–80% confluence. Cells were detached with Accutase (Gibco, catalogue number A1110501), quenched with full-growth DMEM media and centrifuged (200 × *g*, 5 min). Cell pellets were fixed in 0.1% (w/v) formaldehyde in PBS for 2 min at room temperature and then quenched with a final concentration of 110 mM glycine. Cells were centrifuged (300 × *g*, 5 min, 4 °C) and resuspended in cold wash buffer (20 mM HEPES, pH 7.5, 150 mM KCl, 0.5 mM spermidine, cOmplete EDTA-free protease inhibitor cocktail (Roche, catalogue number 11836170001)). Cells were either used directly or stored frozen in wash buffer containing 10% (v/v) DMSO at −80 °C until use. A final density of 6 million cells per ml was used.

G4switch-biotin stock solution in DMSO was diluted to 10 μM in antibody buffer (wash buffer containing 2 mM EDTA, 0.1% bovine serum albumin, 0.05% digitonin). The solution was either kept in the dark or illuminated at 405 nm for 2 min to obtain the *cis* and *trans* G4switch-biotin, respectively. For each sample, 4 μl of 10 μM *cis* or *trans* G4switch-biotin and 3.4 μl anti-biotin (D5A7) rabbit mAb (Cell Signaling Technology, catalogue number 5597S; ~10 μM) were added to 32.6 μl of antibody buffer to make a final volume of 40 μl. The mixture was incubated in the dark or under pulsed 405 nm illumination (1.5 s every 15 s) inside a light-proof box at 4 °C for 1 h to form a high-concentration precomplex. Next, 60 μl of antibody buffer was added to the precomplex, resulting in a final concentration of 0.4 μM precomplex solution based on the G4switch-biotin concentration, with the anti-biotin antibody diluted to a final ratio of ~1:30.

Concanavalin A beads (15 μl) (Bangs Laboratories, catalogue number BP531) per sample were washed twice in binding buffer (20 mM HEPES, pH 7.5, 10 mM KCl, 1 mM CaCl_2_, 1 mM MnCl_2_) and resuspended in 10 μl of binding buffer. A 100 μl cell suspension was incubated with 10 μl of prewashed concanavalin A beads in a 0.2 ml colourless transparent PCR tube on an Intelli Mixer RM-2M (ELMI) set to mode C3 at 20 rpm with the following mixing cycle: 6 s at 108°, 6 s at 252° and 12 s pause at 0°, for 10 min at room temperature. Bead-bound cells were gently washed twice with 100 μl of wash buffer using a magnet stand and resuspended in 100 μl of G4switch-biotin-anti-biotin precomplex solution. The *trans*-G4switch-biotin sample was illuminated at 405 nm with cooling by a desk fan, while the *cis* isomer was kept in the dark by wrapping the tube with foil. Samples without precomplex were used as controls. All samples were incubated on the mixer using the same mixing programme at room temperature for 2 h.

Cells were washed three times in 200 μl Dig-wash buffer (wash buffer containing 0.05% digitonin) to remove unbound antibodies. They were then incubated in 100 μl of a 1:100 dilution of guinea pig anti-rabbit IgG secondary antibody (antibodies-online, catalogue number ABIN101961) in Dig-wash buffer on the mixer using the above mixing programme at room temperature for 1 h, with or without 405 nm illumination with cooling by a desk fan for the *trans* and *cis* probe isomers.

pA-Tn5 conjugated with sequencing adapters were prepared as previously described^49^. pA-Tn5 adapter complex (the pA-Tn5 concentration is 2 μM) is diluted 1:200 into Dig-300 buffer (20 mM HEPES, pH 7.5, 300 mM KCl, 0.5 mM spermidine, 0.01% digitonin, cOmplete EDTA-free protease inhibitor cocktail). Cells were then washed three times in 200 μl Dig-wash buffer to remove unbound antibodies before incubating in 100 μl pA-Tn5 adapter complex solution on the mixer using the above mixing programme at room temperature for 1 h, with or without 405 nm illumination for the *trans* and *cis* probe isomers. Cells were then washed three times with 200 μl Dig-300 buffer to remove unbound pA-Tn5 before incubation in 200 μl tagmentation buffer (Dig-300 buffer containing 10 mM MgCl_2_) on the mixer using the above mixing programme at 37 °C for 1 h, in the presence and absence of 405 nm illumination for the *trans* and *cis* probe isomers.

Cells were washed twice in 200 μl TAPS wash buffer (10 mM TAPS, 0.2 mM EDTA) and resuspended in 100 μl extraction buffer (10 mM Tris-HCl, pH 8.0, 0.5 mg ml^−1^ proteinase K (Thermo Scientific, catalogue number EO0491), 0.5% SDS) with vortexing. The suspension was incubated at 55 °C for 1.5 h at 800 rpm. DNA extraction was performed using the DNA Clean & Concentrator-5 kit (Zymo Research, catalogue number D4013). DNA was eluted in 25 μl of the provided DNA elution buffer after a 10 min incubation at room temperature.

To amplify DNA libraries, PCR was performed by mixing 21 μl tagmented DNA with 2 μl uniquely barcoded v2 Ad1.x primer (10 μM) and 2 μl uniquely barcoded v2 Ad2.x primer (10 μM)^57^, and 25 μl NEBNext Ultra II Q5 2× PCR Master mix (New England Biolabs, catalogue number M0544) using the following cycle: 72 °C for 5 min; 98 °C for 30 s; 10 cycles of 98 °C for 10 s and 63 °C for 10 s; 72 °C for 1 min. Libraries were cleaned up by adding 1.3× volume (65 μl) of Ampure XP beads (Beckman Coulter, catalogue number A63882), incubated at room temperature for 10 min and washed gently twice with 200 μl of 80% ethanol. DNA was eluted in 25 μl of 10 mM Tris-HCl, pH 8.0.

The size distribution of libraries was confirmed by TapeStation analysis using High Sensitivity D1000 ScreenTape (Agilent, catalogue number 5067-5584). Libraries were quantified using the NEBNext Library Quant Kit (New England Biolabs, catalogue number E7630L). Libraries were balanced and pooled, followed by size selection by adding 0.4× volume of Ampure XP beads and incubation at room temperature for 10 min. The supernatant was transferred to a new DNA LoBind tube, and 1.3× volume of Ampure XP beads was then added and incubated at room temperature for 10 min. On-bead libraries were washed twice with 80% ethanol and eluted in 30 μl of 10 mM Tris-HCl, pH 8.0. Paired-end sequencing of libraries was performed on a NextSeq 2000 sequencer (Illumina) using the NextSeq 1000/2000 P2 reagent kit (100 cycles) (Illumina, catalogue number 20046811). Experiments were performed in three independent biological replicates, each with three technical replicates.

For the Chem-map competition experiments, U2OS cells were grown to a 70–80% confluence and treated with PDS (4 μM or 20 μM) or vehicle DMSO at 37 °C for 3 h. Cells were then washed twice with PBS and processed according to the standard G4switch Chem-map protocol. Three independent biological replicates were conducted.

### G4-CUT&Tag

The G4-CUT&Tag was adapted from that previously described^49^, and performed in five replicates. The BG4 (scFv) antibody was expressed as previously reported^11,15^. pA-Tn5 adapter complex was prepared as described previously^49^. Concanavalin A-coated beads were prepared with Dynabeads MyOne Streptavidin T1 beads (Invitrogen, catalogue number 65601) and biotin-conjugated concanavalin A solution (Sigma-Aldrich, catalogue number C2272) as described previously^58^. In brief, U2OS cells (~300,000 cells per 100 μl for each sample) were bound to concanavalin A-conjugated beads (10 μl). The bead-bound cells were permeabilized and incubated in 50 μl of ~600 nM BG4 antibody solution diluted in antibody buffer at 4 °C overnight, followed by an incubation in 50 μl rabbit anti-FLAG antibody (Cell Signaling Technology, catalogue number 2368S) solution at a 1:25 dilution in Dig-wash buffer at room temperature for 1 h. After a further incubation with 50 μl guinea pig anti-rabbit IgG secondary antibody solution at a 1:100 dilution in Dig-wash buffer at room temperature for 1 h, 50 μl pA-Tn5 adapter complex dilution at a 1:250 dilution in Dig-300 buffer was added to cells for incubation at room temperature for 1 h, followed by the tagmentation reaction at 37 °C for 1 h. After proteinase K digestion, the DNA fragments were extracted for the library preparation and Illumina sequencing.

### SLAM-seq

About 2.1 × 10^5^ U2OS cells per well were seeded in a 6-well plate in full-growth DMEM media and grown for 24 h. In one set of experiments, U2OS cells were treated with **9** (6 μM) in 1 ml full-growth DMEM media at 37 °C for 30 min, in the presence and absence of pulsed 405 nm light illumination (90 s of continuous 405 nm illumination for maximum *cis*-to-*trans* photoisomerization, followed by light pulses of 75 ms every 15 s to maintain activation of **9** with minimal light exposure), to determine the effects of *cis* and *trans* isomers, respectively. In a ‘rescue’ experiment, alternating pulses of 405 nm and 525 nm light illumination, which involves 90 s of 405 nm illumination, 120 s of 525 nm illumination, and pulses of alternating phases of 75 ms 405 nm and 375 ms 525 nm every 15 s, to measure the effects of cycles of reversibly photoswitching **9** on and off, ending with the off state. **9** with only pulsed 525 nm light illumination (120 s of continuous 525 nm illumination followed by pulses of 375 ms every 15 s) served as a control. All conditions were compared with a non-illuminated DMSO control. After the 30 min treatment under all conditions, newly synthesized RNA was then labelled by incubating the cells with 250 μM 4sU (Sigma-Aldrich, catalogue number T4509) at 37 °C for 60 min in the dark. Cells were washed twice with 2 ml PBS, followed by RNA extraction using the RNeasy Plus Mini Kit (QIAGEN, catalogue number 74134). Total RNA was quantified by a Nanodrop One (Thermo Fisher Scientific), and 3 μg of RNA was subjected to iodoacetamide alkylation using the SLAMseq Kinetics Kit (Lexogen, catalogue number 061). Alkylated RNA (500 ng) was then used for preparation of 3′-end mRNA sequencing libraries using the QuantSeq 3′ mRNA-Seq Library Prep kit (FWD) (Lexogen, catalogue number 015) and PCR Add-on kit (Lexogen, catalogue number 020) for Illumina. Libraries were sequenced on a NextSeq 500 sequencer (Illumina) using the High Output kit (Illumina, catalogue number FC-404-2005). Four independent biological replicates were performed (*n* = 4).

### Crystal violet staining and cell density quantification

The protocol was adapted from that previously described^59^. In brief, U2OS cells were seeded at a density of 35,000 cells per well in full-growth DMEM media in a 6-well plate and grown for 24 h. Cells were then cultured in the presence of 125 nM **9** compared with vehicle DMSO in 1.5 ml full-growth DMEM media, with half of the wells shielded from the pulsed 405 nm illumination (60 s continuous illumination followed by pulses of 75 ms every 22.5 s) for 72 h. Cells were washed with 2 ml PBS, stained with 1 ml crystal violet solution (0.5% crystal violet in 20% methanol) at room temperature for 10 min. Unbound crystal violet was removed by rinsing in distilled water (2 ml 4×) and cells were subsequently air-dried. Cells were visualized on a Bio-Rad ChemiDoc MP system using the Coomassie Blue Gel channel (590/110, white trans) with 0.5 s manual exposure. Image processing and cell density quantification were performed using the open-source software Fiji (version 2.14.0/1.54f)^60^ (Supplementary Information). Three independent biological replicates were performed (*n* = 3).

### Chem-map and G4-CUT&Tag analysis

Illumina sequencing paired-end output files (Supplementary Tables 1–3) were demultiplexed using demuxIllumina (version 3.0.9) using the following flags: -c -d -i -e -t 1 -r 0.01 -R -l 9. The resulting fastq.gz files underwent sequencing quality control using FastQC (version 0.11.8), and their summary was visualized by MultiQC (version 1.11). Bases with a quality score below 20 were trimmed from both reads using cutadapt (cutadapt -q 20). Fastq files were aligned to the combined hg38 and *Escherichia coli* genomes using bwa (version 0.7.17-r1188) with only reads in the whitelist regions of hg38 continuing the process pipeline. Duplicates were removed using Picard (version 2.20.3) (Picard MarkDuplicates). Peaks were called using Seacr (version 1.3) without input control reporting the top 1% by AUC regions, stringent criteria (Supplementary Tables 4 and 5). BigWig files were created of the bam files, normalized at cpm using deepTools (version 2.0)^61^. Consensus peaks are defined by the overlap of the technical replicates for each biological and two out of three biological replicates for each feature. High-confidence peak regions, unless otherwise stated, are considered those regions at the top 1% by AUC, with minimum total signal 5 (min5) and present in 2 of 3 replicates (multi2). Differential analysis on the union of all the peaks among the examined molecules was carried out using DiffBind (version 3.10.1)^47,62^.

### SLAM-seq analysis

Illumina sequencing single-end output files (Supplementary Table 6) were demultiplexed using demuxIllumina with the following flags: -c -d -i -e -t 1 -r 0.01 -l 9. The quality of the resulting FASTQ files was assessed using FastQC (version 0.11.8), and bases with a quality score below 20 were trimmed from the read using cutadapt (cutadapt -q 20), with adapter overlaps set to 3 bp for trimming.

Gene and 3′ untranslated region annotations were obtained from the UCSC table browser (https://genome.ucsc.edu/cgi-bin/hgTables), with Gencode v37 used for transcript annotation*.*

The SlamDunk package (v.0.3.4) was used to process the trimmed reads to analyse 4sU incorporation events. The full analysis was performed using the ‘Slamdunk all’ command with the following parameters: -t 5 -5 12 -n 100 -m -mv 0.2 -c 2 -rl 100, aligning against the human genome (GRCh38) and filtering for variants with a variant fraction of 0.2. Reads were filtered for having ≥2 T > C conversions, unless otherwise stated.

Differential gene expression of TcReadCounts was restricted to genes with ≥10 reads in at least one condition. DESeq2 (version 1.2.10) was used for differential gene expression analysis on raw read counts with ≥2 T > C conversions using default settings. Size factors were calculated based on corresponding total read counts for global normalization. Volcano plots were generated using the EnhancedVolcano package (v.1.16.0). Plots of differential gene expression were visualized using the ggplot2 package in R (version 4.1) with significant genes (*q* ≤ 0.05, |log2FC| ≥ 1).

### Reporting summary

Further information on research design is available in the Nature Portfolio Reporting Summary linked to this article.

## Online content

Any methods, additional references, Nature Portfolio reporting summaries, source data, extended data, supplementary information, acknowledgements, peer review information; details of author contributions and competing interests; and statements of data and code availability are available at 10.1038/s41557-025-01792-1.

## Supplementary information

Supplementary Information General information, other experimental methods, Supplementary Figs. 1–8, Tables 1–7, synthetic procedures, NMR spectra and characterization of the emission spectra of 405 nm and 525 nm LED lights.

Reporting Summary

Supplementary Data 1 The expression of all genes downregulated (*q* ≤ 0.05) by **9** under all treatment conditions, compared with the dark-adapted DMSO control.

Supplementary Data 2 Source data for Supplementary Fig. 3.

## Source data

Source Data Fig. 2 Statistical source data.

Source Data Fig. 3 Statistical source data.

Source Data Fig. 5 Unprocessed image.

Source Data Fig. 5 Statistical source data.

Source Data Extended Data Fig. 1 Statistical source data.

Source Data Extended Data Fig. 2 Statistical source data.

Source Data Extended Data Fig. 3 Statistical source data.

Source Data Extended Data Fig. 4 Statistical source data.

Source Data Extended Data Fig. 4 Unprocessed western blots.

## Data Availability

Sequencing data reported in this study have been deposited in the NCBI Gene Expression Omnibus (GEO) repository under accession number GSE261837. The previously published GRCh38 (hg38) (https://www.ensembl.org/Homo_sapiens/Info/Index) and OQs (GSE110582)^48^ datasets were used. Source data are provided with this paper.

## Code availability

All detailed bioinformatics scripts are available at https://github.com/sblab-informatics/G4-switch.
