# Supplementary material for: Optical control of gene expression using a DNA G-quadruplex targeting reversible photoswitch
Source: Nat Chem. 2025 Apr 3;17(6):875–82. doi: 10.1038/s41557-025-01792-1 (PMC12141046; doi:10.1038/s41557-025-01792-1)
Supplement: Supplementary file 1 — General information, other experimental methods, Supplementary Figs. 1–8, Tables 1–7, synthetic procedures, NMR spectra and characterization of the emission spectra of 405 nm and 525 nm LED lights. [file 41557_2025_1792_MOESM1_ESM.pdf]

nature chemistry

Supplementary information https://doi.org/10.1038/s41557-025-01792-1

Optical control of gene expression using a DNA G-quadruplex targeting reversible photoswitch

In the format provided by the authors and unedited

Table of Contents

General information 2
Experimental methods 2
Supplementary Fig. 1 9
Supplementary Fig. 2 10
Supplementary Fig. 3 11
Supplementary Fig. 4 12
Supplementary Fig. 5 13
Supplementary Fig. 6 14
Supplementary Fig. 7 15
Supplementary Fig. 8 16
Supplementary Table 1 17
Supplementary Table 2 18
Supplementary Table 3 19
Supplementary Table 4 20
Supplementary Table 5 21
Supplementary Table 6 22
Supplementary Table 7 23
Synthetic procedures 24
Synthesis of putative G4switches 24
Synthesis of G4switch-biotin 30
NMR spectra 33
Characterization of the emission spectra of 405 nm and 525 nm LED lights 48
References 50
Source Data for Supplementary Fig. 8 51

General information
All chemical reagents were used as purchased from commercial sources unless otherwise stated. Organic solvents were either distilled by standard purification methods or purchased as anhydrous from commercial sources. All reactions were performed in oven-dried glassware under argon atmosphere unless otherwise specified. NMR spectra were recorded on a Bruker 400 MHz Avance III HD Smart Probe Spectrometer, a 500 MHz Avance III Smart Probe Spectrometer, or a 700 MHz TXO Cryoprobe Spectrometer, operating at 400, 500 and 700 MHz for ^{1}H NMR, and at 100, 125 and 175 MHz for ^{13}C NMR, respectively in the indicated solvents. NMR data were processed using MestReNova (version 12.0.1) and were reported as follows: chemical shifts in parts per million (ppm) referred to the solvent residual peak, peak multiplicities (s = singlet, d = doublet, t = triplet, q = quartet; m = multiplet, br = broad), and coupling constants in Hz. LC-MS analysis was performed using a Dionex UltiMate 3000 UHPLC system (Thermo Fisher Scientific) coupled to an Amazon ESI-MS (Bruker). High-resolution mass spectra (HRMS) were measured using a Waters LCT Premier (ESI) mass spectrometer. Flash column chromatography was performed using a CombiFlash NEXTGEN 300+ system (TELEDYNE ISCO) with RediSep normal-phase silica flash columns. Reversed-phase flash chromatography was performed using an Interchim puriFlash 4250 system with a C18 flash column (catalogue no. PF-15C18HP-F0025, 15 µm particle size).

NHS-PEG4-Biotin (catalogue no. 21363) was purchased from Thermo Scientific. 6,6'-(ethane-1,2-diyl)bis(3-bromoaniline) (**S1**)[1] and *tert*-butyl (2-((2-aminoquinolin-4-yl)oxy)ethyl)carbamate (**S4**)[2] were prepared as described previously. ChemAxon MarvinSketch (http://www.chemaxon.com) was used to calculate the number of trifluoroacetic acid (TFA) molecules for compounds in the form of a TFA salt at pH 2 (the pH of 0.1% v/v TFA-containing mobile phase solvents used in the reversed-phase chromatography).

Experimental methods
Photoisomerizations. For the ^{1}H NMR experiments and FID assay, photoisomerizations were performed using EvoluChem LED lights (HepatoChem, catalogue no. HCK1012-01-010 for 405 nm; HCK1012-01-004 for 525 nm) at distances of approximately 10 cm and 40 cm, respectively. For circular dichroism (CD) titration experiments, UV-Vis spectroscopy and cell studies, photoisomerizations were performed using LED lights from Sahlmann Photochemical Solutions (405 nm, NVSU233A-U405, FWHM: 13 nm, 3 × 900 mW; 525 nm, Nichia NCSG219B-V1, FWHM: 32 nm, 3 × 600 mW) at a distance of approximately 10 cm.

UV-Vis spectroscopy. UV-Vis absorption spectra were acquired on an Agilent Cary 3500 UV-Vis spectrophotometer using a 400 µl quartz cuvette with a 10 mm path length at 25 °C. Settings: 200-700 nm range, 0.02 s averaging time, 1 nm data intervals, and 1 nm bandwidth. A 50 µM solution of compound **9** in PBS was measured before and after 1 min of 405 or 525 nm illumination. Recorded spectra were baseline-corrected against the buffer, zero-adjusted by subtracting the average absorption value in the 650–700 nm range, and plotted using GraphPad Prism 10 (version 10.2.2).

Cell viability assay. U2OS cells were seeded in a 96-well black-wall transparent flat-bottom plate at a density of 7,000 cells per well in 100 µl full growth DMEM media and grown at 37 °C

for 20-24 h. Cells were treated with 0-100 μM of **9** either in the dark or under indicated illumination conditions employed in the SLAM-seq experiments for 30 min, followed by 60 min of incubation with 250 μM 4-thiouridine (4sU) in the dark. Cell viability was determined using the CellTiter-Glo 2.0 cell viability assay (Promega, catalogue no. G9242). CellTiter-Glo 2.0 reagent (100 μl per well) was added and equilibrated at room temperature for 30 minutes before measuring end-point luminescence on a multi-mode plate reader (BMG PHERAstar Plus). The absorbance was subtracted from the media blank and converted to cell viability (%) relative to the no-ligand control. The dose-dependent cell viability was calculated using GraphPad Prism 10 (version 10.2.2). Results are shown as mean ± s.d. from four replicates (n = 4).

Quantification of relative cell viability. TIFF image files were opened in FIJI (version 2.14.0/1.54f)[3] and converted from RGB to greyscale (Image→Type→8-bit). Considering light in the edge areas could be partially shielded by plate walls, only cells in the central circular areas were selected for quantification. The regions of interest (ROI) manager was opened (Analyse→Tools→ROI Manager), and "Show all" and "Labels" were selected. A circular region (w = 0.95 (285), h = 0.95 (285)) was created on one well of the 6-well plate image using the oval selection tool and added to the ROI manager. The same-sized circular selection was applied to all wells. Measurements were set up by choosing the following parameters: "Area", "Min & max grey value", "Mean gray value", "Centroid", "Limit to threshold", and "Display label". The settings were configured as "Redirect to: None" and "Decimal places (0-9): 3". Images were inverted (Edit→Invert), and the threshold was adjusted (Image→Adjust→Threshold: 85 (used in this study); select "Dark background"). The "Measure" in the ROI Manager was clicked to obtain the area values representing the cell density in the Results window. Relative cell viability was calculated by normalising the cell area to that of the DMSO treatment in the dark.

Protein extraction and Western blot analysis. U2OS cells were seeded at 50,000 cells/well in 6-well plates using full growth DMEM media and cultured for 24 h. Cells were then treated with 125 nM of compound **9** or vehicle DMSO in 1.5 mL of full growth media, with or without 405 nm pulsed light (60 s continuous, followed by 75 ms pulses every 22.5 s) for 72 h. PDS (10 μM) and Camptothecin (10 μM) treatments in the dark for 72 h and 5 h, respectively, were used as positive controls. After treatment, cells were washed with PBS, detached with Accutase (Gibco, catalogue no. A1110501), and cell pellets were collected. Following another cold PBS wash, cells were lysed in RIPA buffer (Thermo Scientific, catalogue no. 89900) with protease inhibitor cocktail (ThermoFisher, catalogue no. 78438) and phosphatase inhibitor cocktail 3 (Sigma Aldrich, catalogue no. P0044). Lysates were sonicated using a Diagenode Bioruptor Plus (5 cycles: 30 s ON, 30 s OFF, high setting, 4°C), then centrifuged at 14,000 g for 15 min at 4°C to collect the supernatant as protein extracts. Protein concentrations were measured using the BCA assay, and samples were analysed by capillary electrophoresis on a Jess automated Western blot system (ProteinSimple) following the manufacturer's protocol (https://www.bio-techne.com/instruments/simple-western). The primary antibodies used for Western blotting in this study were rabbit anti-γH2AX (Cell Signaling Technology, catalogue

no. 2577) at 1:10 dilution, rabbit anti-PARP-1 (Cell Signaling Technology, catalogue no. 9532) at 1:400 dilution and rabbit anti-β-Actin (Cell Signaling Technology, catalogue no. 4970) at 1:1000 dilution. Similar results were obtained in one of three independent biological replicates.

a

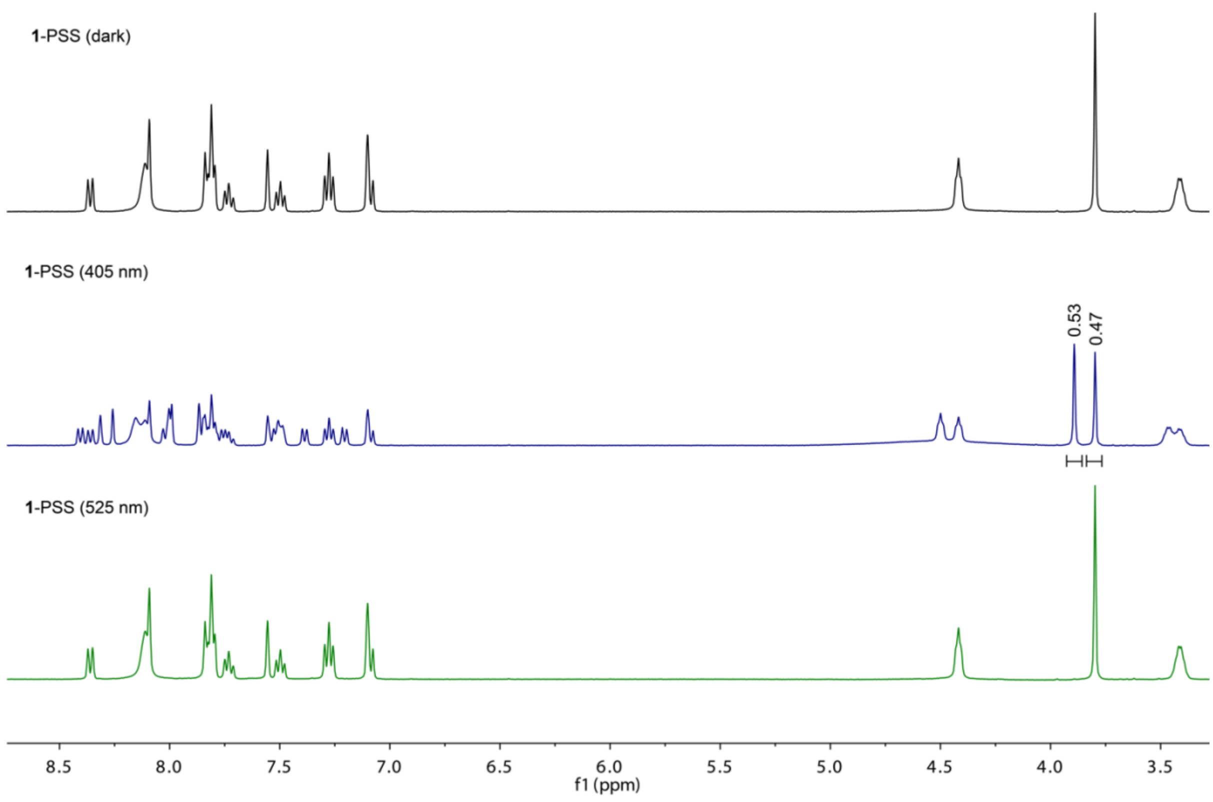

1-PSS (dark)
1-PSS (405 nm)
0.53
0.47
1-PSS (525 nm)
8.5
8.0
7.5
7.0
6.5
6.0
5.5
5.0
4.5
4.0
3.5
f1 (ppm)

b

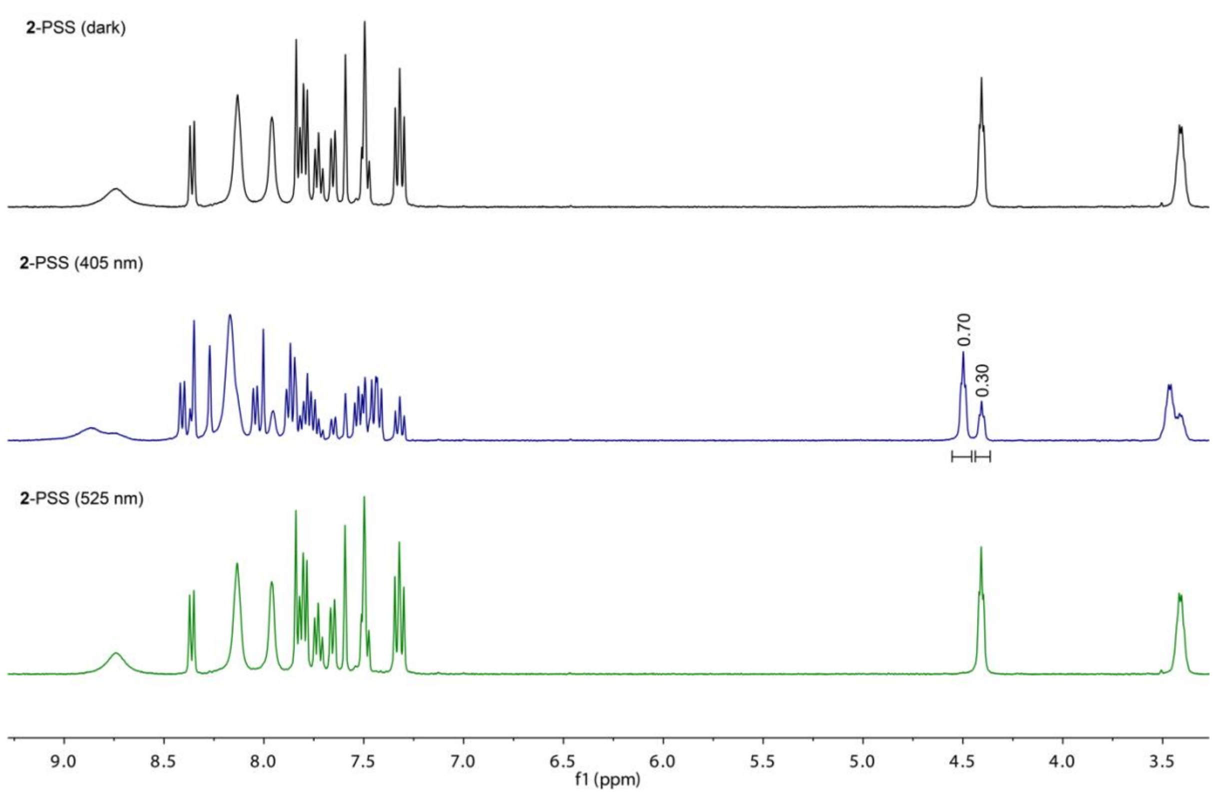

2-PSS (dark)
2-PSS (405 nm)
0.70
0.30
2-PSS (525 nm)
9.0
8.5
8.0
7.5
7.0
6.5
6.0
5.5
5.0
4.5
4.0
3.5
f1 (ppm)

c

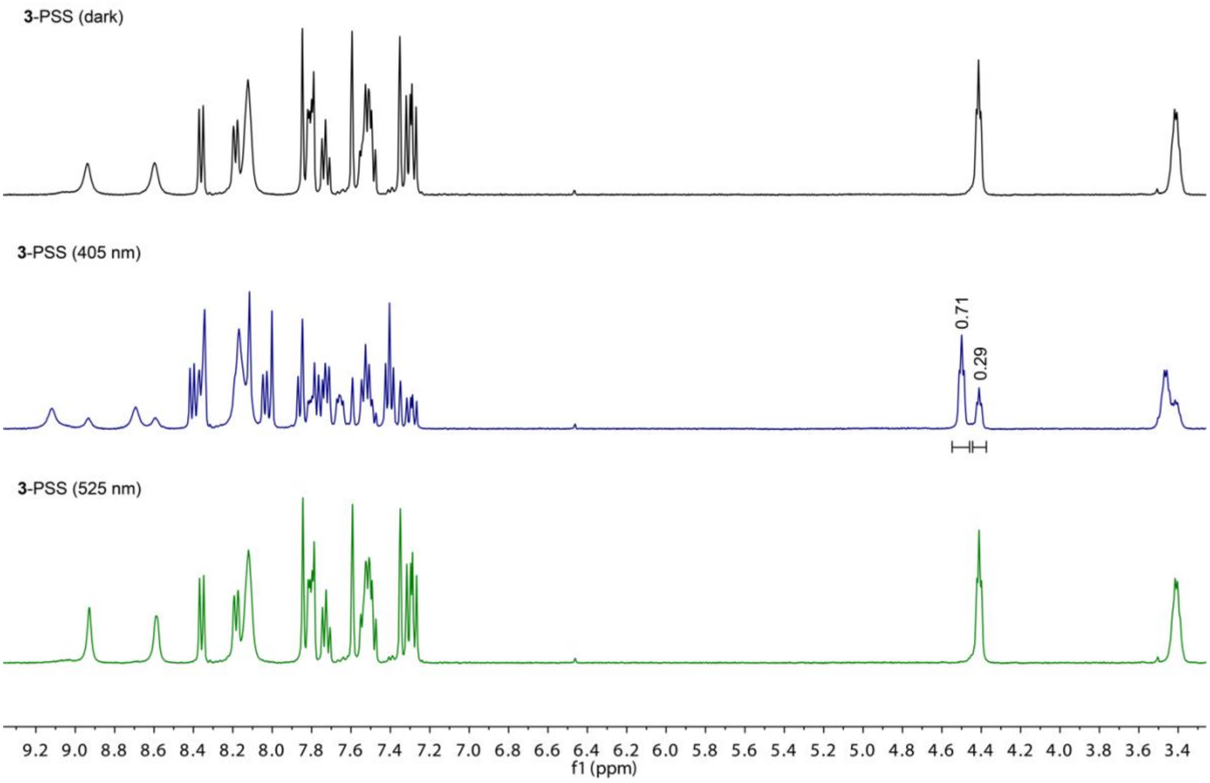

3-PSS (dark)
3-PSS (405 nm)
0.71
0.29
3-PSS (525 nm)
9.2 9.0 8.8 8.6 8.4 8.2 8.0 7.8 7.6 7.4 7.2 7.0 6.8 6.6 6.4 6.2 6.0 5.8 5.6 5.4 5.2 5.0 4.8 4.6 4.4 4.2 4.0 3.8 3.6 3.4
f1 (ppm)

d

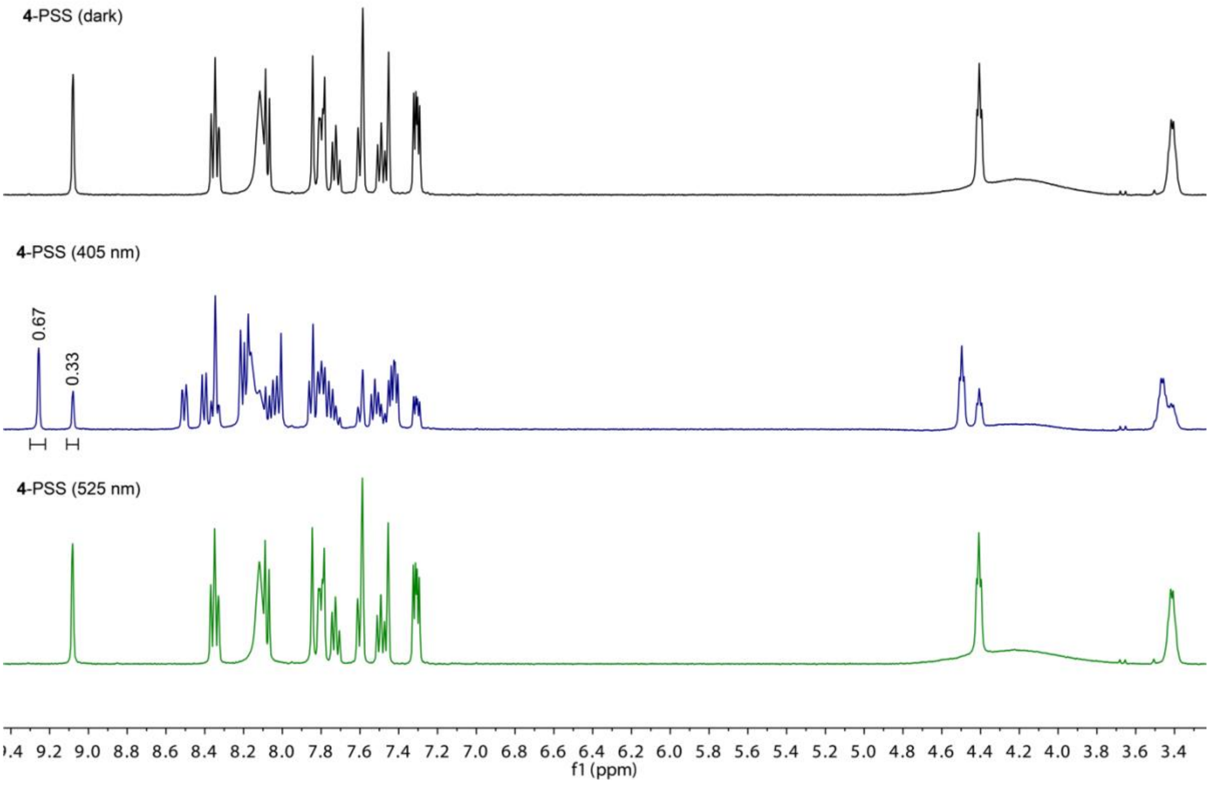

4-PSS (dark)
4-PSS (405 nm)
0.67
0.33
4-PSS (525 nm)
9.2 9.0 8.8 8.6 8.4 8.2 8.0 7.8 7.6 7.4 7.2 7.0 6.8 6.6 6.4 6.2 6.0 5.8 5.6 5.4 5.2 5.0 4.8 4.6 4.4 4.2 4.0 3.8 3.6 3.4
f1 (ppm)

e

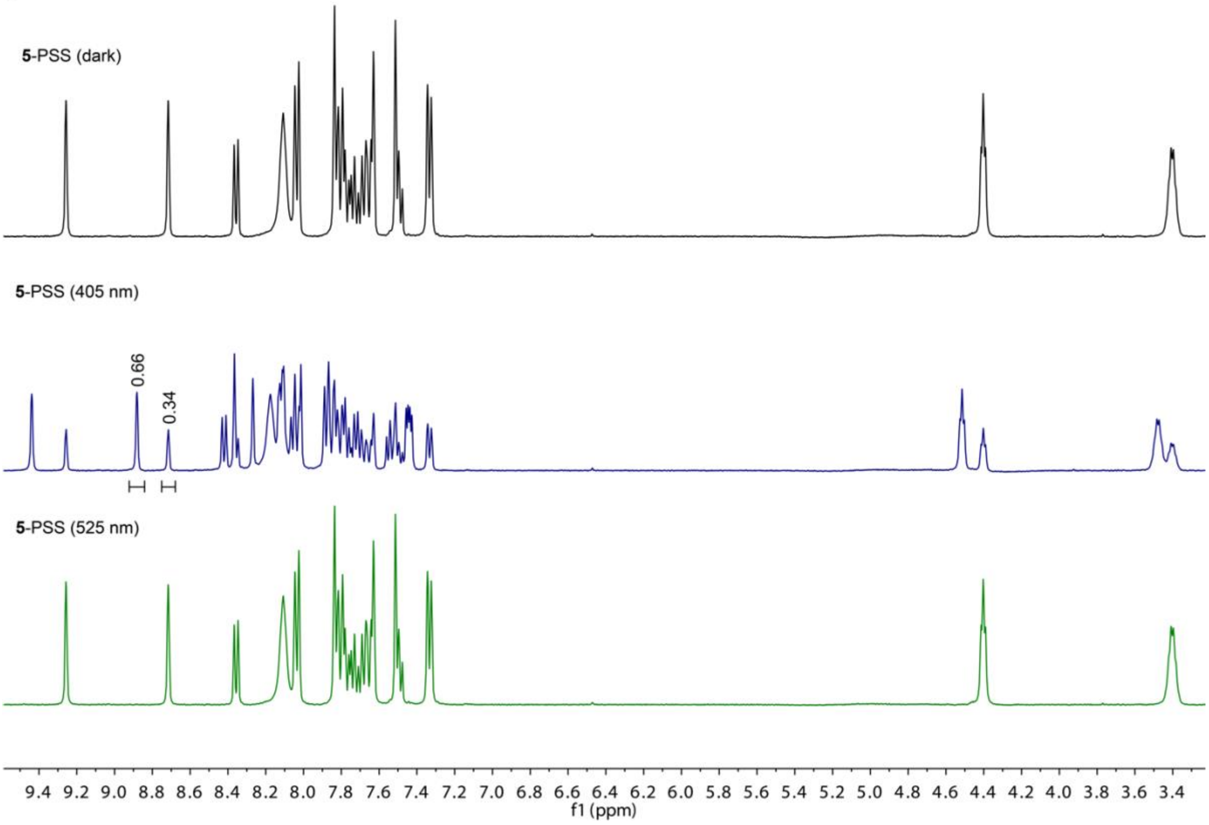

5-PSS (dark)
5-PSS (405 nm)
0.66
0.34
5-PSS (525 nm)
9.4 9.2 9.0 8.8 8.6 8.4 8.2 8.0 7.8 7.6 7.4 7.2 7.0 6.8 6.6 6.4 6.2 6.0 5.8 5.6 5.4 5.2 5.0 4.8 4.6 4.4 4.2 4.0 3.8 3.6 3.4
f1 (ppm)

f

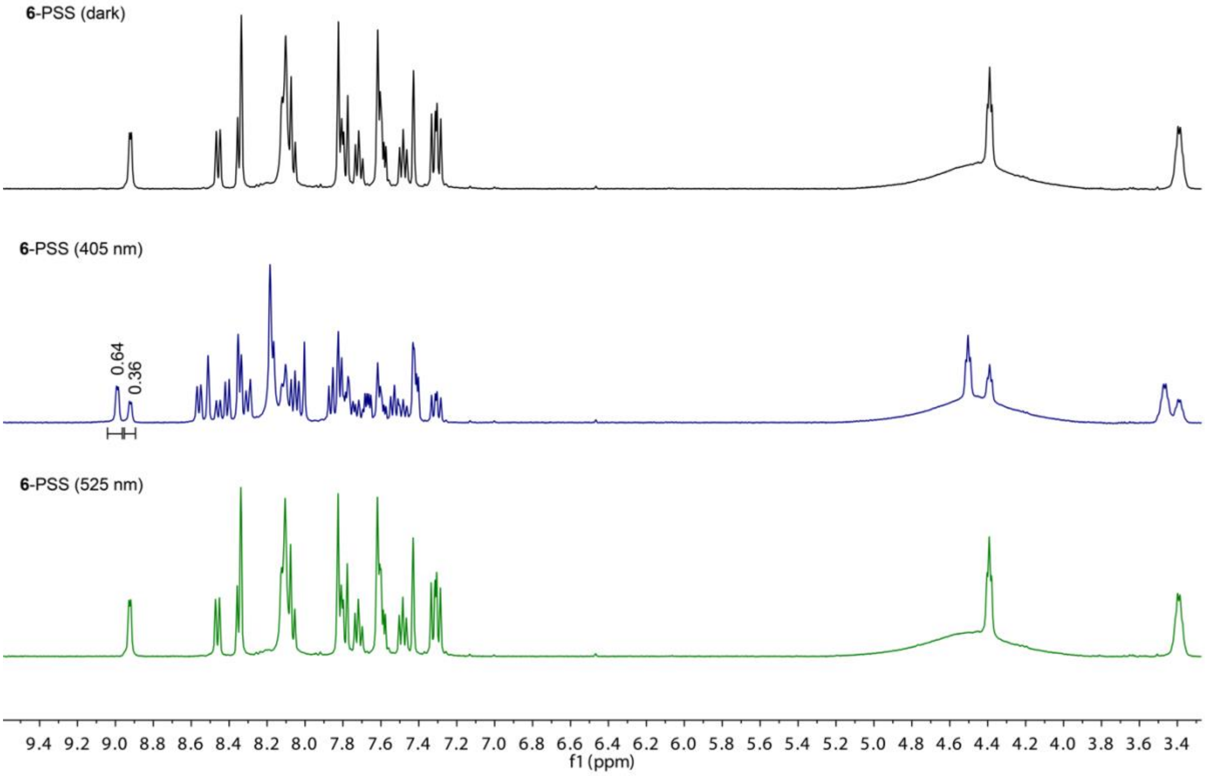

6-PSS (dark)
6-PSS (405 nm)
0.64
0.36
6-PSS (525 nm)
9.4 9.2 9.0 8.8 8.6 8.4 8.2 8.0 7.8 7.6 7.4 7.2 7.0 6.8 6.6 6.4 6.2 6.0 5.8 5.6 5.4 5.2 5.0 4.8 4.6 4.4 4.2 4.0 3.8 3.6 3.4
f1 (ppm)

g

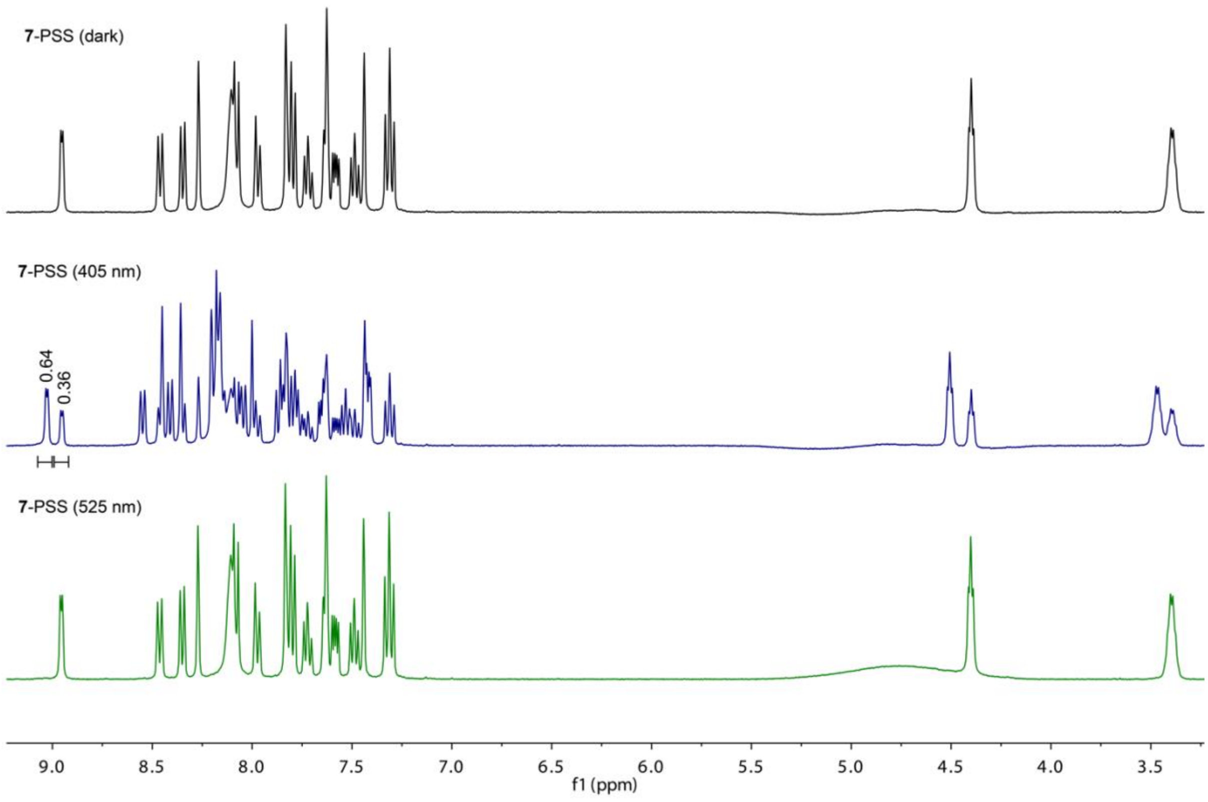
7-PSS (dark)
7-PSS (405 nm)
0.64
0.36
7-PSS (525 nm)
9.0 8.5 8.0 7.5 7.0 6.5 6.0 5.5 5.0 4.5 4.0 3.5
f1 (ppm)

h

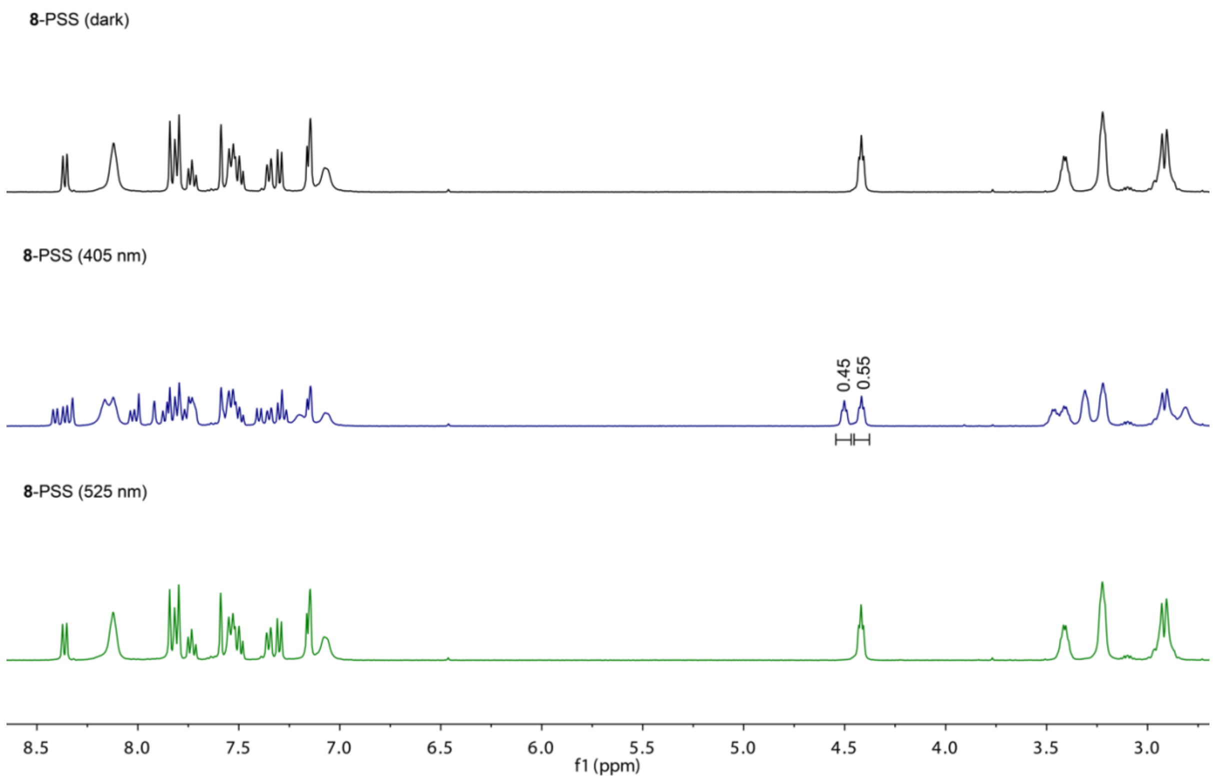
8-PSS (dark)
8-PSS (405 nm)
0.45
0.55
8-PSS (525 nm)
8.5 8.0 7.5 7.0 6.5 6.0 5.5 5.0 4.5 4.0 3.5 3.0
f1 (ppm)

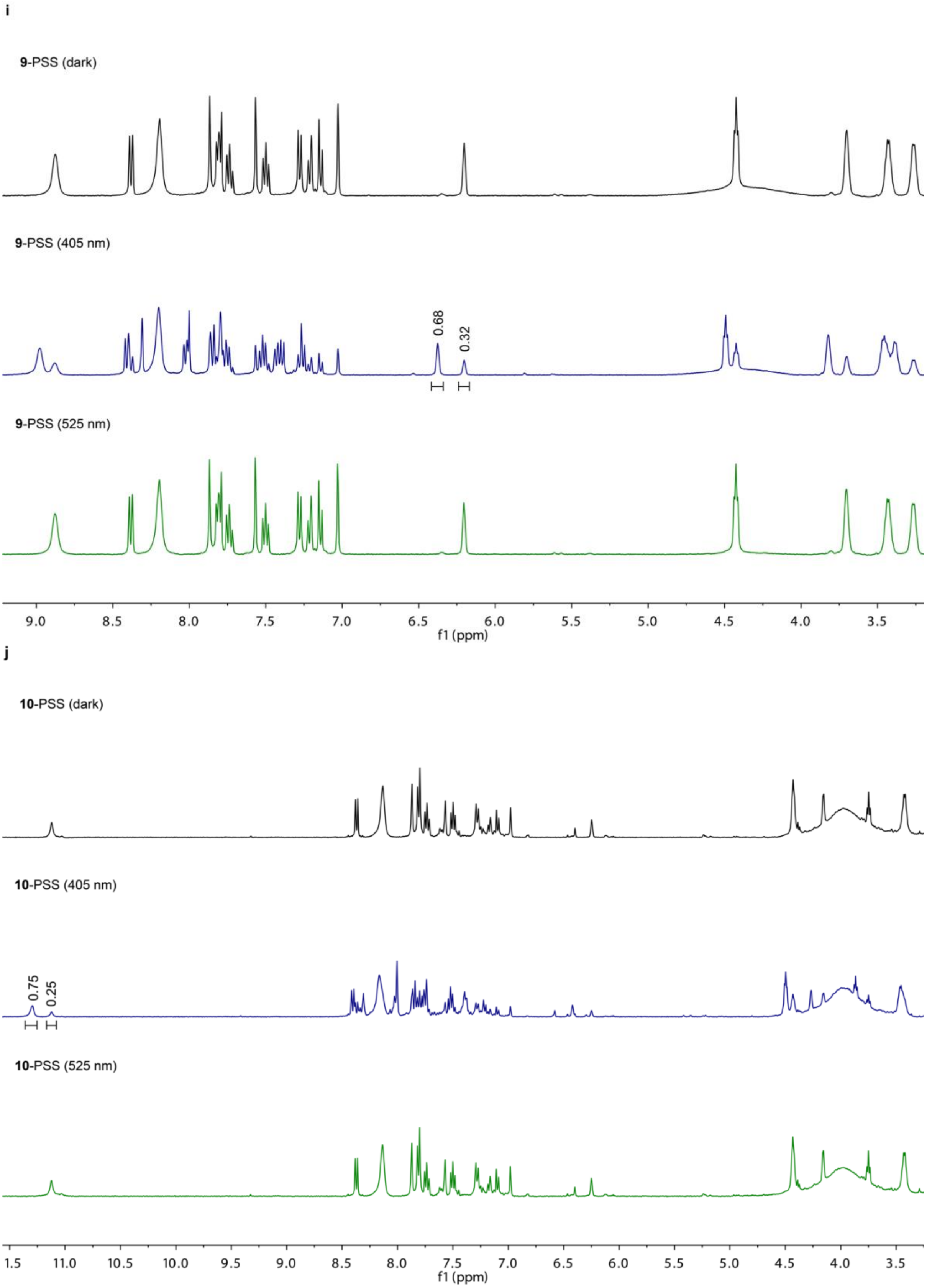

Supplementary Fig. 1 | Photoswitching of putative G4switches. a-k, ^{1}H NMR spectra of compounds **1-10** at ~10 mM in DMSO-d_6 before and after 405 nm illumination, and after subsequent 525 nm illumination. The photostationary states (PSS) of compounds are shown.

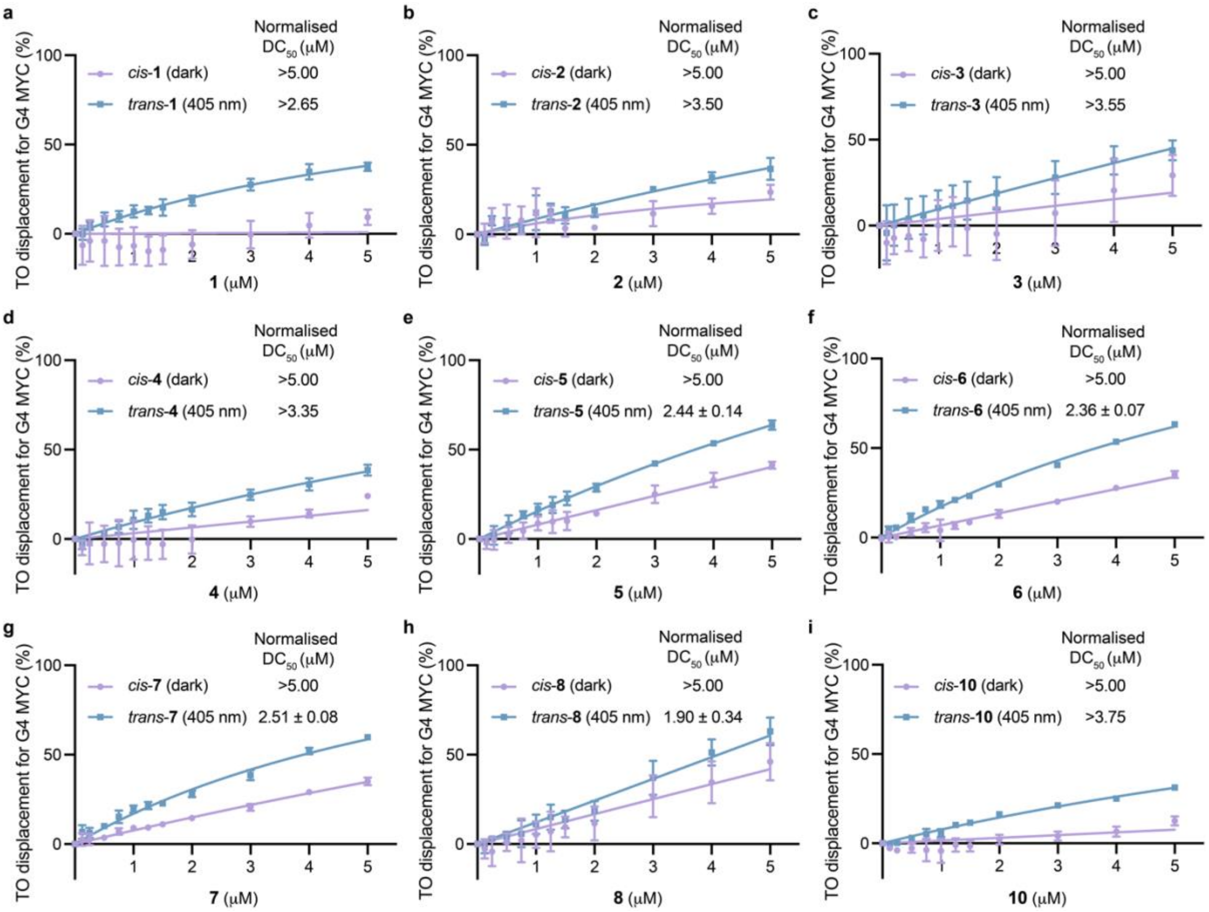

Supplementary Fig. 2 | Assessment of putative G4switches binding to a G4 structure. a-i, FID plots for compounds **1**-**8**, **10** bound to the G4 MYC structure to displace the G4 binder thiazole orange (TO) before and after 405 nm illumination. The DC_{50} values, normalised from the corresponding concentrations of isomers (see Supplementary Fig. 1), are shown. Mean ± s.d. are represented from four replicates (n = 4).

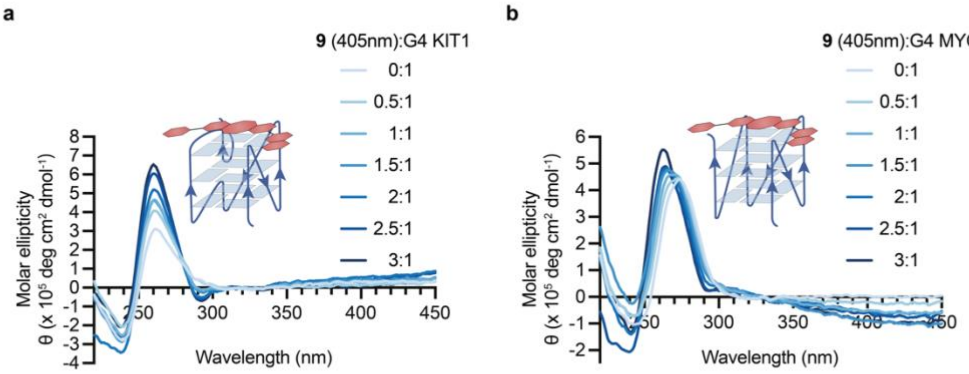

Supplementary Fig. 3 | In vitro G4 stabilisation by *tran*-9. CD spectra (220-450 nm) of 10 μM (a) G4 KIT1 and (b) G4 MYC recorded in the presence of 0-3 molar equivalents of 405 nm light-activated **9** in 10 mM Tris-HCl buffer, 100 mM LiCl, pH 7.4.

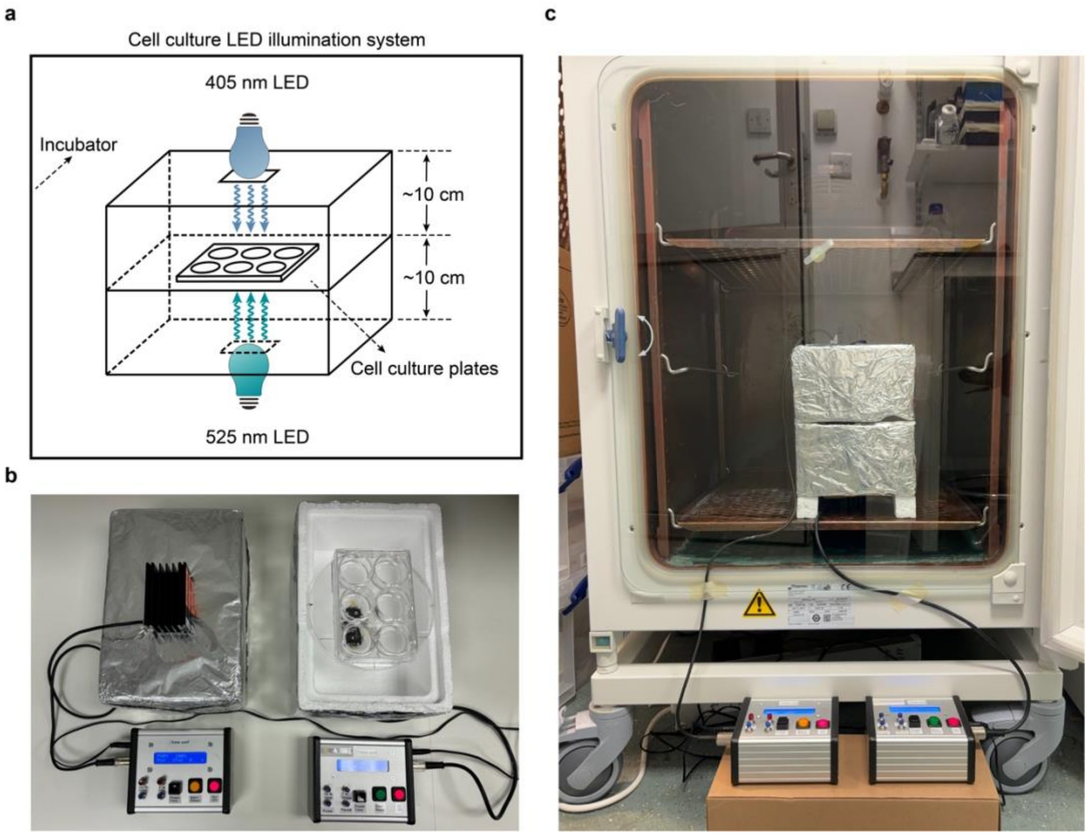

Supplementary Fig. 4 | LED illumination system for cell culture experiments. a, Schematic layout of the LED illumination system enabling single or alternating phases of pulsed 405 nm (top) and 525 nm (bottom) illuminations of cultured cells in plates within the incubator. **b,** Controllers, LED modules, and self-built light-proof boxes used for 405 nm (top) and 525 nm (bottom) illuminations. **c**, Practical setup of the LED illumination system: controllers are placed outside of the incubator, while power-supplied LEDs with leads fit onto rectangle holes on the top and bottom of box inside the incubator, illuminating the cultured cells in the middle of the box.

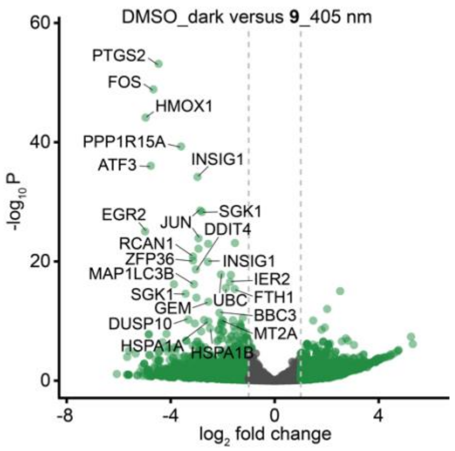

Supplementary Fig. 5 | Optical control of transcription using 9 in live cells. Volcano plot displaying genes with significantly altered expression (highlighted in green) (fold change >2, q ≤ 0.05) by **9** in the presence of pulsed 405 nm illumination, relative to a dark-adapted DMSO vehicle control.

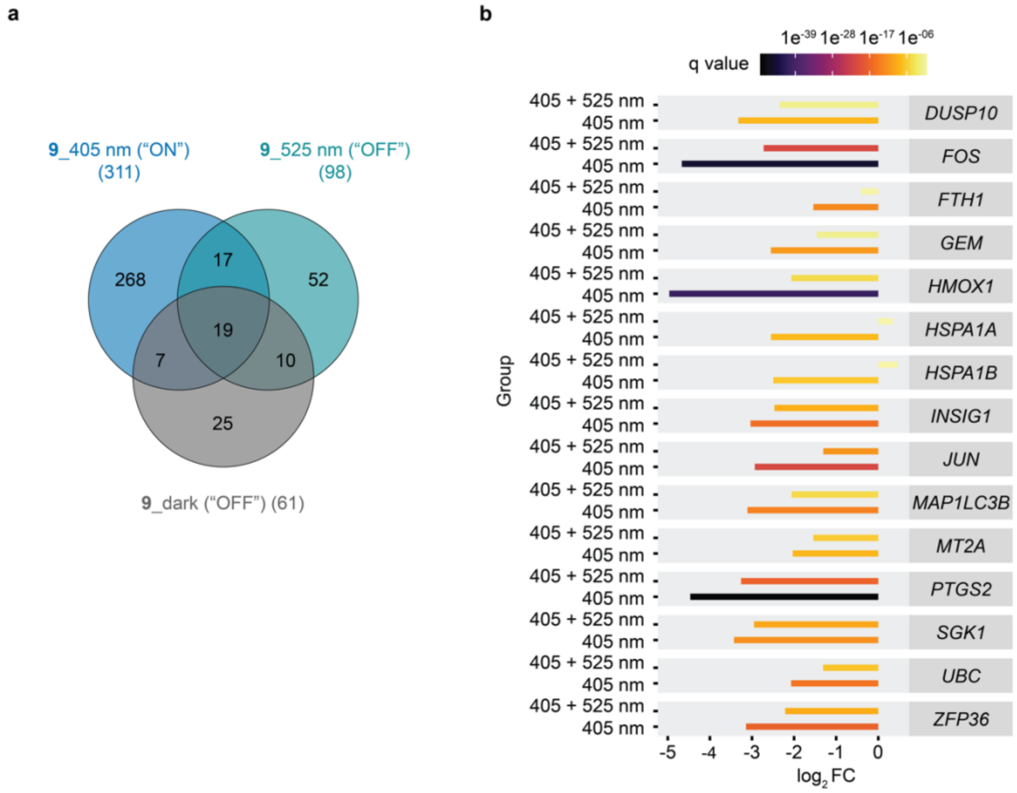

Supplementary Fig. 6 | a, Venn diagram displaying the overlap of down-regulated genes ($q \leq 0.05$) by **9** between dark treatment and 405 nm illumination, and 525 nm illumination (a green light control). **9** in dark treatment impacted as few genes as under the 525 nm illumination condition, with 61 and 98 genes, respectively. For the expression of all altered genes under all conditions, see Supplementary Data 1. **b**, Bar plot comparing gene expression of randomly selected down-regulated genes from Extended Data Fig. 4c with that under the 405 + 525 nm illumination, showing some degree of recovery upon 405 + 525 nm illumination (see Supplementary Table 7).

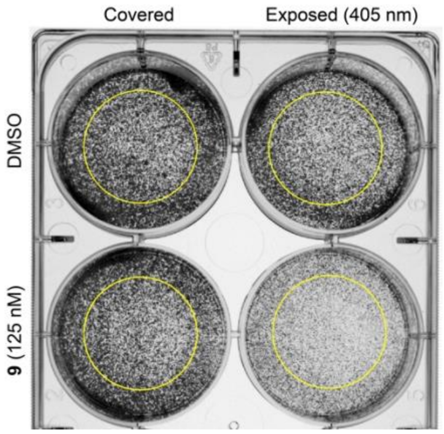

Supplementary Fig. 7 | G4switch-mediated spatial control of U2OS cell proliferation. Images showing spatial control of U2OS cell proliferation in the presence of **9** compared to vehicle DMSO within the same 6-well plate. The half-covered plate with a black mask was exposed to pulsed 405 nm LED illumination (60 s continuous illumination, followed by pulses of 75 ms every 22.5 s) for 72 h, followed by crystal violet staining. The areas highlighted with yellow circles (w = 0.95 (285), h = 0.95 (285) based on the 6-well plate image) were selected for the quantification of cell density. A representative image from one of three independent biological replicates with similar results is shown.

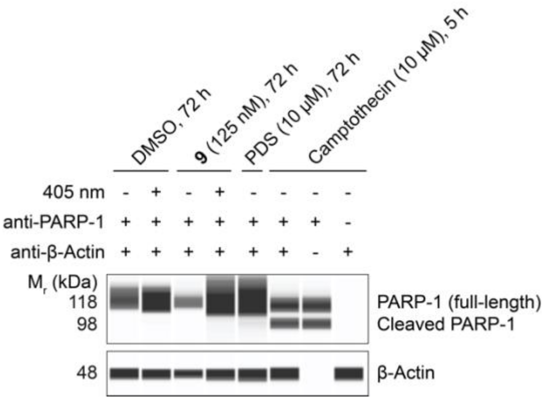

Supplementary Fig. 8 | Western blotting of full-length and cleaved PARP-1. Apoptosis marker cleaved PARP-1 was examined by Western blot after treating cells with compound **9** (125 nM) with or without 405 nm illumination (60 s continuous illumination, followed by pulses of 75 ms every 22.5 s) and its parent molecule PDS (10 μM) for 72 h, compared to the DMSO control in the dark. Cells treated with Camptothecin, a DNA topoisomerase I inhibitor, for 5 h served as a positive control. A representative image from one of three independent biological replicates with similar results is shown.

Supplementary Table 1. Read statistics of Chem-map of G4switch (**9**)

**Sample**	**Number of reads (million)**
SLX23282_CM_U2OS_G4switch400nM_rt_2h_405nm.B1.t1	27.7
SLX23282_CM_U2OS_G4switch400nM_rt_2h_405nm.B1.t2	25.1
SLX23282_CM_U2OS_G4switch400nM_rt_2h_405nm.B1.t3	27.4
SLX23282_CM_U2OS_G4switch400nM_rt_2h_405nm.B2.t1	41.3
SLX23282_CM_U2OS_G4switch400nM_rt_2h_405nm.B2.t2	23.6
SLX23282_CM_U2OS_G4switch400nM_rt_2h_405nm.B2.t3	29.2
SLX23282_CM_U2OS_G4switch400nM_rt_2h_405nm.B3.t1	32.9
SLX23282_CM_U2OS_G4switch400nM_rt_2h_405nm.B3.t2	17.5
SLX23282_CM_U2OS_G4switch400nM_rt_2h_405nm.B3.t3	33.7
SLX23282_CM_U2OS_G4switch400nM_rt_2h_dark.B1.t1	26.8
SLX23282_CM_U2OS_G4switch400nM_rt_2h_dark.B1.t2	33.3
SLX23282_CM_U2OS_G4switch400nM_rt_2h_dark.B1.t3	22.8
SLX23282_CM_U2OS_G4switch400nM_rt_2h_dark.B2.t1	26.5
SLX23282_CM_U2OS_G4switch400nM_rt_2h_dark.B2.t2	33.8
SLX23282_CM_U2OS_G4switch400nM_rt_2h_dark.B2.t3	27.5
SLX23282_CM_U2OS_G4switch400nM_rt_2h_dark.B3.t1	21.0
SLX23282_CM_U2OS_G4switch400nM_rt_2h_dark.B3.t2	35.0
SLX23282_CM_U2OS_G4switch400nM_rt_2h_dark.B3.t3	31.0
SLX23282_CM_U2OS_no1stAb_405nm.B1.t1	1.8
SLX23282_CM_U2OS_no1stAb_405nm.B2.t1	0.9
SLX23282_CM_U2OS_no1stAb_405nm.B3.t1	0.2

Supplementary Table 2. Read statistics of Chem-map competition

**Sample**	**Number of reads (million)**
SLX23283_CM_U2OS_DMSO_3h_G4switch400nM_2h_405nm.B1.t1	24.3
SLX23283_CM_U2OS_DMSO_3h_G4switch400nM_2h_405nm.B2.t1	29.9
SLX23283_CM_U2OS_DMSO_3h_G4switch400nM_2h_405nm.B3.t1	38.3
SLX23283_CM_U2OS_PDS_20uM_3h_G4switch400nM_2h_405nm.B1.t1	29.5
SLX23283_CM_U2OS_PDS_20uM_3h_G4switch400nM_2h_405nm.B2.t1	25.0
SLX23283_CM_U2OS_PDS_20uM_3h_G4switch400nM_2h_405nm.B3.t1	31.8
SLX23283_CM_U2OS_PDS_4uM_3h_G4switch400nM_2h_405nm.B1.t1	21.9
SLX23283_CM_U2OS_PDS_4uM_3h_G4switch400nM_2h_405nm.B2.t1	28.9
SLX23283_CM_U2OS_PDS_4uM_3h_G4switch400nM_2h_405nm.B3.t1	26.0

Supplementary Table 3. Read statistics of G4-CUT&Tag

Sample	Number of reads (million)
SLX22701_BG4_CnT_U2OS_DMSO_noUV.B1.t1	16.5
SLX22701_BG4_CnT_U2OS_DMSO_noUV.B1.t2	13.2
SLX22701_BG4_CnT_U2OS_DMSO_noUV.B1.t3	10.9
SLX22701_BG4_CnT_U2OS_DMSO_noUV.B1.t4	12.0
SLX22701_BG4_CnT_U2OS_DMSO_noUV.B1.t5	23.6

Supplementary Table 4. Individual Chem-map peak numbers of **9** for q-value < 0.01

**Sample**	**Number of reads (million)**
SLX23282_CM_U2OS_G4switch400nM_rt_2h_405nm.PCR-10.B1.t1.merged.min0.bed	15420
SLX23282_CM_U2OS_G4switch400nM_rt_2h_405nm.PCR-10.B1.t2.merged.min0.bed	15075
SLX23282_CM_U2OS_G4switch400nM_rt_2h_405nm.PCR-10.B1.t3.merged.min0.bed	15556
SLX23282_CM_U2OS_G4switch400nM_rt_2h_405nm.PCR-10.B2.t1.merged.min0.bed	18968
SLX23282_CM_U2OS_G4switch400nM_rt_2h_405nm.PCR-10.B2.t2.merged.min0.bed	16544
SLX23282_CM_U2OS_G4switch400nM_rt_2h_405nm.PCR-10.B2.t3.merged.min0.bed	16748
SLX23282_CM_U2OS_G4switch400nM_rt_2h_405nm.PCR-10.B3.t1.merged.min0.bed	15586
SLX23282_CM_U2OS_G4switch400nM_rt_2h_405nm.PCR-10.B3.t2.merged.min0.bed	14815
SLX23282_CM_U2OS_G4switch400nM_rt_2h_405nm.PCR-10.B3.t3.merged.min0.bed	20182
SLX23282_CM_U2OS_G4switch400nM_rt_2h_dark.PCR-10.B1.t1.merged.min0.bed	10084
SLX23282_CM_U2OS_G4switch400nM_rt_2h_dark.PCR-10.B1.t2.merged.min0.bed	9214
SLX23282_CM_U2OS_G4switch400nM_rt_2h_dark.PCR-10.B1.t3.merged.min0.bed	7790
SLX23282_CM_U2OS_G4switch400nM_rt_2h_dark.PCR-10.B2.t1.merged.min0.bed	6056
SLX23282_CM_U2OS_G4switch400nM_rt_2h_dark.PCR-10.B2.t2.merged.min0.bed	6505
SLX23282_CM_U2OS_G4switch400nM_rt_2h_dark.PCR-10.B2.t3.merged.min0.bed	5471
SLX23282_CM_U2OS_G4switch400nM_rt_2h_dark.PCR-10.B3.t1.merged.min0.bed	4152
SLX23282_CM_U2OS_G4switch400nM_rt_2h_dark.PCR-10.B3.t2.merged.min0.bed	7497
SLX23282_CM_U2OS_G4switch400nM_rt_2h_dark.PCR-10.B3.t3.merged.min0.bed	5560
SLX23282_CM_U2OS_no1stAb_405nm.PCR-10.B1.t1.merged.min0.bed	2692
SLX23282_CM_U2OS_no1stAb_405nm.PCR-10.B2.t1.merged.min0.bed	1193
SLX23282_CM_U2OS_no1stAb_405nm.PCR-10.B3.t1.merged.min0.bed	271

Supplementary Table 5. Individual G4-CUT&Tag peak numbers for q-value < 0.01

**Sample**	**Number of peaks**
SLX22701_bg4_CnT_U2OS_DMSO_noUV_BG4.PCR-10.B1.t1.sortName.1000.clean.fragments.bedgraph.0.01fdr.stringent.bed	19866
SLX22701_bg4_CnT_U2OS_DMSO_noUV_BG4.PCR-10.B1.t2.sortName.1000.clean.fragments.bedgraph.0.01fdr.stringent.bed	17752
SLX22701_bg4_CnT_U2OS_DMSO_noUV_BG4.PCR-10.B1.t3.sortName.1000.clean.fragments.bedgraph.0.01fdr.stringent.bed	15372
SLX22701_bg4_CnT_U2OS_DMSO_noUV_BG4.PCR-10.B1.t4.sortName.1000.clean.fragments.bedgraph.0.01fdr.stringent.bed	16377
SLX22701_bg4_CnT_U2OS_DMSO_noUV_BG4.PCR-10.B1.t5.sortName.1000.clean.fragments.bedgraph.0.01fdr.stringent.bed	24248

Supplementary Table 6. Read statistics of SLAM-Seq

Sample	Number of reads (million)
DMSO_dark_rep1	20.0
DMSO_dark_rep2	20.4
DMSO_dark_rep3	20.3
DMSO_dark_rep4	20.7
G4switch_dark_rep1	21.7
G4switch_dark_rep2	20.5
G4switch_dark_rep3	19.7
G4switch_dark_rep4	21.8
G4switch_405nm_rep1	22.0
G4switch_405nm_rep2	21.7
G4switch_405nm_rep3	21.0
G4switch_405nm_rep4	21.3
G4switch_405+525nm_rep1	19.8
G4switch_405+525nm_rep2	21.1
G4switch_405+525nm_rep3	21.3
G4switch_405+525nm_rep4	22.2
G4switch_525nm_rep1	19.7
G4switch_525nm_rep2	20.2
G4switch_525nm_rep3	22.4
G4switch_525nm_rep4	21.6

Supplementary Table 7. Differential gene expression analysis of several randomly selected, most significantly down-regulated (fold change >2, q ≤ 0.05, p < 10^{-8}) by **9** under 405nm illumination and 405 + 525nm illumination conditions, compared to the dark-adapted DMSO control.

**Gene**	**Group**	**log_2FC**	**p value**	**q value**
HMOX1	405 nm	-4.9637688	7.53E-45	1.32E-41
HMOX1	405 + 525 nm	-2.0616672	5.83E-08	2.17E-05
HSPA1A	405 nm	-2.5475758	1.16E-10	1.88E-08
HSPA1A	405 + 525 nm	0.34767601	0.15532852	0.99850264
HSPA1B	405 nm	-2.4932821	1.70E-09	2.37E-07
HSPA1B	405 + 525 nm	0.46415788	0.14147088	0.99850264
FTH1	405 nm	-1.5409914	4.16E-16	1.11E-13
FTH1	405 + 525 nm	-0.4064771	0.0555774	0.80070036
FOS	405 nm	-4.6664421	1.36E-49	5.59E-46
FOS	405 + 525 nm	-2.7215229	3.12E-25	7.62E-22
PTGS2	405 nm	-4.465427	7.19E-54	4.43E-50
PTGS2	405 + 525 nm	-3.2590585	2.20E-21	4.76E-18
JUN	405 nm	-2.9321298	1.31E-24	7.66E-22
JUN	405 + 525 nm	-1.3050695	1.87E-15	2.08E-12
DUSP10	405 nm	-3.3235362	5.72E-11	1.04E-08
DUSP10	405 + 525 nm	-2.3407642	0.00018837	0.02646106
MT2A	405 nm	-2.0314566	6.04E-11	1.06E-08
MT2A	405 + 525 nm	-1.5401364	1.36E-09	6.58E-07
ZFP36	405 nm	-3.1406259	7.08E-21	2.81E-18
ZFP36	405 + 525 nm	-2.2123322	1.18E-12	8.66E-10
MAP1LC3B	405 nm	-3.1073789	6.75E-17	1.93E-14
MAP1LC3B	405 + 525 nm	-2.0583198	4.50E-08	1.74E-05
GEM	405 nm	-2.5505189	5.73E-14	1.28E-11
GEM	405 + 525 nm	-1.4589405	0.00038382	0.04084232
UBC	405 nm	-2.0704807	1.54E-18	5.27E-16
UBC	405 + 525 nm	-1.3102272	2.35E-10	1.51E-07
SGK1	405 nm	-3.4273425	2.65E-15	6.15E-13
SGK1	405 + 525 nm	-2.9525523	3.17E-13	2.43E-10
INSIG1	405 nm	-3.0333591	2.17E-19	7.86E-17
INSIG1	405 + 525 nm	-2.4661347	2.15E-12	1.50E-09

Synthetic procedures
Synthesis of putative G4switches

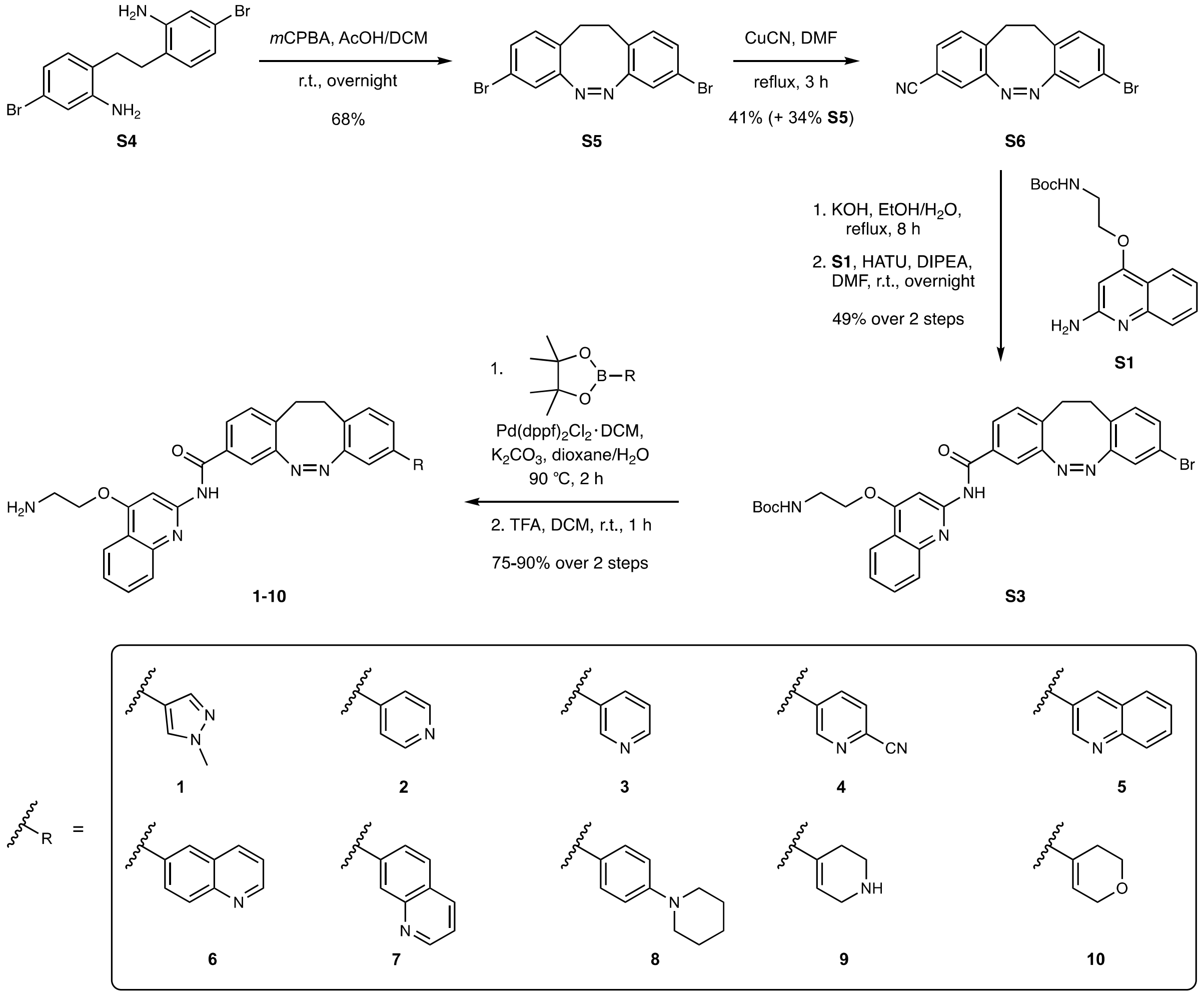

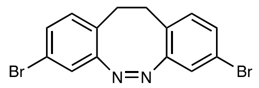

S5

Compound S5: A solution of *m*CPBA in acetic acid was freshly prepared and titrated as described previously[4]. 0.58 M *m*CPBA solution (6.00 mmol) was added via a syringe pump into a rapidly stirred solution of **S4** (1.11 g, 3.00 mmol) in acetic acid/dichloromethane = 1/3 (75 ml) over a period of 12 h. After the complete addition, the mixture was stirred for at least one more hour. The solvent was then removed under reduced pressure. The residue was taken up in ethyl acetate (50 ml) and washed with sat. $NaHCO_3$ (20 ml × 2) and brine (10 ml). The solution was dried over anhydrous $MgSO_4$ and filtered. The solvents were removed in vacuo and the residue was purified by flash silica column chromatography (0 ~ 3% EtOAc in petroleum ether) to give the title compound **S5** as a yellow solid (747 mg, 2.04 mmol, 68% yield): ^1H NMR (400 MHz $CDCl_3$): δ 7.17 (dd, *J* = 8.0 Hz, 2.0 Hz, 2H), 7.01 (d, *J* = 2.0 Hz, 2H), 6.86 (d, *J* = 8.4 Hz, 2H), 3.96 – 2.66 (m, 4H); HRMS (ESI-TOF): $[M+H]^+$ calculated for $C_{14}H_{11}Br_2N_2$: 364.9289, found: 364.9289. The spectroscopic data are in agreement with previously reported values[1].

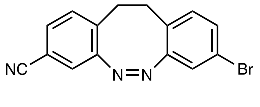

S6

Compound S6: To a solution of compound **S5** (747 mg, 2.04 mmol) in dry DMF (5 ml) was added CuCN (183 mg, 2.04 mmol). The mixture was stirred under reflux for 3 h. The solution was then allowed to cool down to room temperature, and a solution of 10% aqueous ethylenediamine (50 ml) was added. The resulting solution was extracted with dichloromethane (50 ml × 3), and the combined organic layers were washed with brine (10 ml), dried over anhydrous $MgSO_4$ and filtered. The solvents were removed in vacuo and the residue was purified by flash silica column chromatography (0 ~ 30% EtOAc in petroleum ether) to give the title compound **S6** as a yellow solid (261 mg, 836 μmol, 41% yield, 34% **S5** recovered): ^{1}H NMR (400 MHz $CDCl_3$): δ 7.34 (dd, *J* = 8.0, 1.6 Hz, 1H), 7.18 (dd, *J* = 8.0, 2.0 Hz, 1H), 7.13 (d, *J* = 1.6 Hz, 1H), 7.12 (d, *J* = 8.0 Hz, 1H), 7.02 (d, *J* = 2.0 Hz, 1H), 6.86 (d, *J* = 8.0 Hz, 1H), 3.07 – 2.70 (m, 4H); HRMS (ESI-TOF): $[M+H]^+$ calculated for $C_{15}H_{11}BrN_3$: 312.0136, found: 312.0132. The spectroscopic data are in agreement with previously reported values[1].

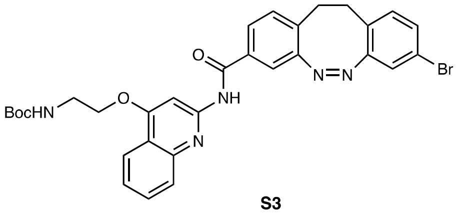

S3

Compound S3: A solution of KOH (4.20 g, 74.9 mmol) in H_2O (4.2 mL) was added into a solution of **S6** (208 mg, 670 μmol) in EtOH (5 ml). The mixture was stirred under reflux for 8 h. The reaction solution was then acidified to pH 4 with 2 N HCl, and the resulting solution was extracted with EtOAc (70 ml × 3). The combined organic layers were washed with brine (10 ml), dried over anhydrous $MgSO_4$ and filtered. The solvent was removed in vacuo to give the crude carboxylic acid product, the total amount of which was used directly without further purification. The crude product was dissolved in dry DMF (5 ml). *N*,*N*-diisopropylethylamine (232 µl, 1.33 mmol) and HATU (279 mg, 734 μmol) were added into the solution. The mixture was stirred at room temperature for 1 h, and the *tert*-butyl (2-((2-aminoquinolin-4-yl)oxy)ethyl)carbamate (**S1**) (212 mg, 700 μmol) was added. The reaction mixture was stirred at room temperature overnight. The DMF was removed in vacuo and the residue was taken up in EtOAc (50 ml). The solution was washed with H_2O (10 ml × 2) and brine (5 ml), dried over anhydrous $MgSO_4$ and filtered. The solvents were removed in vacuo and the residue was purified by flash silica column chromatography (10 ~ 40% EtOAc in petroleum ether) to give the title compound **S3** as a yellow solid (203 mg, 329 μmol, 49% yield over two steps): ^{1}H NMR (500 MHz, $CDCl_3$) δ 8.15 (d, *J* = 8.0 Hz, 1H), 7.95 (s, 1H), 7.78 (d, *J* = 8.0 Hz, 1H), 7.75 (d, *J* = 8.5 Hz, 1H), 7.69 (t, *J* = 7.0 Hz, 1H), 7.52 (d, *J* = 1.0 Hz, 1H), 7.44 (t, *J* = 7.5 Hz, 1H), 7.18 – 7.13 (m, 2H), 7.03 (d, *J* = 2.0 Hz, 1H), 6.87 (d, *J* = 8.0 Hz, 1H), 5.03 (s, 1H), 4.35 (t, *J* = 5.0 Hz, 2H), 3.77 – 3.63 (m, 2H), 3.07 – 2.72 (m, 4H), 1.45 (s, 9H); ^{13}C NMR (125 MHz $CDCl_3$) δ 165.7, 164.1, 155.9, 155.8, 155.3, 152.2, 144.3, 133.1, 132.4, 131.4, 131.2, 130.52, 130.46, 126.6, 126.4, 125.1, 124.9, 122.4, 121.8, 120.4, 119.2, 118.5, 93.9, 79.8, 68.7, 39.6, 31.5, 30.9, 28.3; HRMS (ESI-TOF): $[M+H]^+$ calculated for $C_{31}H_{31}BrN_5O_4$: 616.1559, found: 616.1573.

General procedure – Suzuki–Miyaura cross-coupling and *N*-Boc deprotection

To a solution of diazocine **S3** (30.0 μmol) and commercially available pinacol boronic ester substrate (33.0 μmol) in dioxane/H_2O (2 ml/0.4 ml) was added $Pd(dppf)Cl_2 \cdot CH_2Cl_2$ (2.5 mg, 3.00 μmol) and potassium carbonate (12.5 mg, 90.0 μmol). The mixture was stirred at 90 °C

under argon atmosphere for 2 h. The reaction was allowed to cool down to room temperature. The mixture was diluted with EtOAc (10 ml), and the resulting solution was filtered through a short pad of Celite. The filtrate was washed with H_2O (5 ml × 2) and brine (5 ml), dried over anhydrous $MgSO_4$ and filtered. The solvents were removed in vacuo to afford the crude product, the total amount of which was used without further purification. The crude product was dissolved in CH_2Cl_2 (1.6 ml), and TFA (0.4 ml) was then added. The mixture was stirred at room temperature for 1 h. The solvents were removed in vacuo and the residue was purified by reversed-phase flash chromatography with 5% ~ 100% acetonitrile (containing 0.1% TFA) in H_2O (containing 0.1% TFA) at a flow rate of 20 ml/min over ~30 min. The acetonitrile was removed in vacuo, and the remaining solvents were removed by freeze-drying to yield the TFA salt of the desired product.

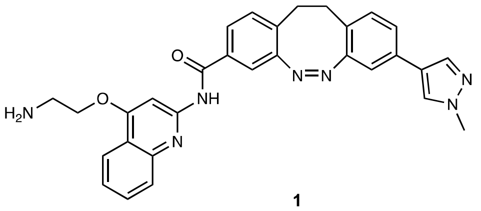

1

Compound 1: Prepared from diazocine **S3** (18.5 mg, 30.0 μmol) and 1-methylpyrazole-4-boronic acid pinacol ester (9.8 mg, 45.0 μmol) according to the general procedure. The TFA salt of the title compound **1** was obtained as a light yellow solid (**1**·2TFA, 19.5 mg, 26.2 μmol, 87% yield over two steps): ^{1}H NMR (500 MHz, Methanol-d_4) δ 8.45 (d, J = 8.0 Hz, 1H), 8.00 (d, J = 8.5 Hz, 1H), 7.92 (ddd, J = 8.5, 7.0, 1.5 Hz, 1H), 7.89 (s, 1H), 7.81 (dd, J = 8.0, 2.0 Hz, 1H), 7.73 (s, 1H), 7.70 – 7.65 (m, 1H), 7.59 (d, J = 2.0 Hz, 1H), 7.53 (s, 1H), 7.32 (d, J = 8.0 Hz, 1H), 7.23 (dd, J = 8.0, 2.0 Hz, 1H), 7.08 – 7.02 (m, 2H), 4.66 (t, J = 5.0 Hz, 2H), 3.86 (s, 3H), 3.62 (t, J = 5.0 Hz, 2H), 3.05 – 2.84 (m, 4H);^{13}C NMR (125 MHz, Methanol-d_4) δ 169.1, 167.7, 157.1, 157.0, 153.1, 139.8, 137.3, 136.5, 134.9, 133.0, 132.1, 131.8, 131.7, 129.3, 128.3, 128.1, 127.0, 125.4, 124.3, 123.2, 122.4, 119.9, 119.7, 116.1, 94.8, 68.1, 39.6, 38.9, 32.5, 31.9; HRMS (ESI-TOF): [M+H]$^+$ calculated for $C_{30}H_{28}N_7O_2$: 518.2304, found: 518.2328.

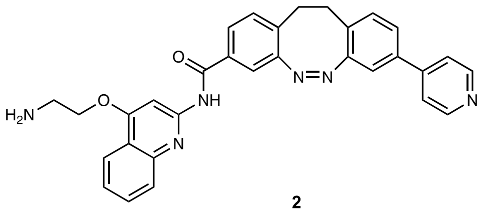

2

Compound 2: Prepared from diazocine **S3** (18.5 mg, 30.0 μmol) and 4-pyridineboronic acid pinacol (6.9 mg, 33.0 μmol) according to the general procedure. The TFA salt of the title compound **2** was obtained as a light yellow solid (**2**·3TFA, 21.0 mg, 24.5 μmol, 82% yield over two steps): ^{1}H NMR (500 MHz, Methanol-d_4) δ 8.70 (br, 2H), 8.41 (dd, J = 8.5, 1.5 Hz, 1H), 8.14 (br, 2H), 7.96 (d, J = 8.5 Hz, 1H), 7.88 (ddd, J = 8.5, 7.0, 1.5 Hz, 1H), 7.79 (dd, J = 8.0, 2.0 Hz, 1H), 7.65 – 7.58 (m, 3H), 7.50 (s, 1H), 7.44 (d, J = 2.0 Hz, 1H), 7.27 (t, J = 8.0 Hz, 2H), 4.63 (t, J = 5.0 Hz, 2H), 3.58 (t, J = 5.0 Hz, 2H), 3.04 – 2.93 (m, 4H); ^{13}C NMR (125 MHz, Methanol-d_4) δ 168.9, 167.7, 157.5, 156.8, 156.2, 153.1, 144.2, 139.9, 135.9, 135.2, 134.9, 133.5, 132.7, 132.3, 131.9, 128.5, 128.2, 127.9, 124.4, 122.3, 120.0, 119.7, 119.2, 94.9, 68.1, 39.6, 32.3, 32.1; HRMS (ESI-TOF): [M+H]$^+$ calculated for $C_{31}H_{27}N_6O_2$: 515.2195, found: 515.2205.

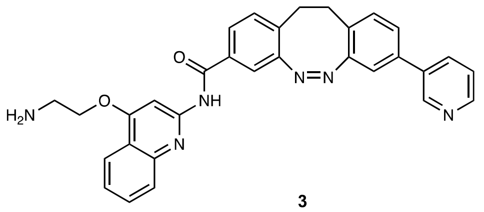

3

Compound 3: Prepared from diazocine **S3** (18.5 mg, 30.0 μmol) and 3-pyridineboronic acid pinacol (6.9 mg, 33.0 μmol) according to the general procedure. The TFA salt of the title compound **3** was obtained as a light yellow solid (**3**·3TFA, 23.2 mg, 27.1 μmol, 90% yield over two steps): ^{1}H NMR (500 MHz, Methanol-*d*4) δ 9.01 (br, 1H), 8.71 (br, 1H), 8.57 (d, *J* = 8.0 Hz, 1H), 8.47 (dd, *J* = 8.5, 0.5 Hz, 1H), 8.04 (d, *J* = 8.5 Hz, 1H), 7.98 – 7.93 (m, 1H), 7.90 (br, 1H), 7.85 (dd, *J* = 8.0, 2.0 Hz, 1H), 7.74 – 7.68 (m, 1H), 7.66 (d, *J* = 1.5 Hz, 1H), 7.51 – 7.47 (m, 2H), 7.36 – 7.31 (m, 2H), 7.29 (d, *J* = 8.0 Hz, 1H), 4.68 (t, *J* = 5.0 Hz, 2H), 3.62 (t, *J* = 5.0 Hz, 2H), 3.11 – 2.95 (m, 4H); ^{13}C NMR (125 MHz, Methanol-*d*4) δ 169.1, 167.9, 157.5, 156.9, 153.1, 143.9, 143.3, 142.4, 139.6, 136.2, 135.2, 135.1, 132.5, 132.2, 131.9, 131.1, 128.39, 128.38, 127.4, 124.4, 122.2, 120.1, 119.7, 118.6, 94.8, 68.2, 39.6, 32.4, 32.0; HRMS (ESI-TOF): $[M+H]^+$ calculated for $C_{31}H_{27}N_6O_2$: 515.2195, found: 515.2195.

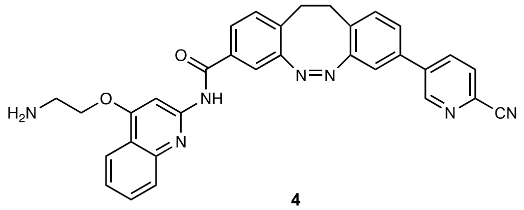

4

Compound 4: Prepared from diazocine **S3** (18.5 mg, 30.0 μmol) and 2-cyanopyridine-5-boronic acid pinacol ester (8.7 mg, 35.0 μmol) according to the general procedure. The TFA salt of the title compound **4** was obtained as a light yellow solid (**4**·2TFA, 19.2 mg, 25.0 μmol, 83% yield over two steps): ^{1}H NMR (500 MHz, DMSO-*d*6) δ 11.14 (br, 1H), 9.08 (dd, *J* = 2.5, 0.5 Hz, 1H), 8.36 (dd, *J* = 8.0, 1.0 Hz, 1H), 8.33 (dd, *J* = 8.0, 2.0 Hz, 1H), 8.20 – 8.10 (m, 3H), 8.07 (dd, *J* = 8.0, 0.5 Hz, 1H), 7.85 – 7.78 (m, 3H), 7.73 (ddd, *J* = 8.5, 7.0, 1.5 Hz, 1H), 7.62 – 7.57 (m, 2H), 7.50 (ddd, *J* = 8.0, 7.0, 1.5 Hz, 1H), 7.45 (d, *J* = 2.0 Hz, 1H), 7.32 (d, *J* = 3.0 Hz, 1H), 7.30 (d, *J* = 3.0 Hz, 1H), 4.41 (t, *J* = 5.0 Hz, 2H), 3.45 – 3.37 (m, 2H), 3.01 – 2.92 (m, 4H); ^{13}C NMR (125 MHz, DMSO-*d*6) δ 165.5, 161.6, 155.6, 154.7, 152.7, 149.2, 146.4, 137.6, 135.3, 133.8, 132.6, 132.4, 131.3, 131.1, 130.7, 130.2, 129.3, 129.1, 127.1, 126.4, 126.2, 124.4, 122.6, 118.8, 118.6, 117.6, 117.4, 95.1, 65.2, 38.2, 30.8, 30.5; HRMS (ESI-TOF): $[M+H]^+$ calculated for $C_{32}H_{26}N_7O_2$: 540.2148, found: 540.2164.

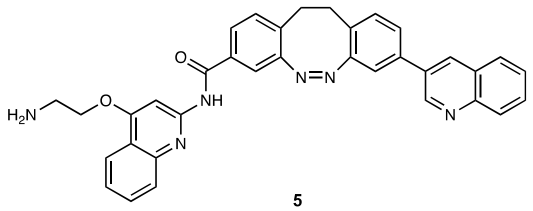

5

Compound 5: Prepared from diazocine **S3** (18.5 mg, 30.0 μmol) and quinoline-3-boronic acid pinacol ester (8.7 mg, 33.0 μmol) according to the general procedure. The TFA salt of the title compound **5** was obtained as a light yellow solid (**5**·3TFA, 23.5 mg, 25.9 μmol, 86% yield over two steps): ^{1}H NMR (500 MHz, Methanol-*d*4) δ 9.22 (d, *J* = 2.0 Hz, 1H), 8.84 (d, *J* = 2.0 Hz, 1H), 8.44 (dd, *J* = 8.5, 1.0 Hz, 1H), 8.12 – 8.06 (m, 2H), 8.00 (d, *J* = 8.5, 1H), 7.94 – 7.87 (m, 2H), 7.84 (dd, *J* = 8.0, 2.0 Hz, 1H), 7.73 (ddd, *J* = 8.5, 7.0, 1.5 Hz, 1H), 7.70 – 7.65 (m, 2H), 7.58 (dd, *J* = 8.0, 2.0 Hz, 1H), 7.48 (s, 1H), 7.42 (d, *J* = 2.0 Hz, 1H), 7.35 (d, *J* = 8.0 Hz, 1H), 7.29 (d, *J* = 8.0 Hz, 1H), 4.65 (t, *J* = 5.0 Hz, 2H), 3.60 (t, *J* = 5.0 Hz, 2H), 3.11 – 2.97 (m, 4H); ^{13}C NMR (125 MHz, Methanol-*d*4) δ 168.9, 167.3, 157.5, 157.0, 153.2, 148.3, 144.6, 140.6, 138.5, 136.5, 136.1, 134.7, 133.0, 132.42, 132.38, 131.9, 130.2, 130.0, 129.9, 129.7, 128.3, 128.1,

127.4, 126.3, 124.2, 122.9, 120.0, 119.7, 118.5, 94.9, 67.9, 39.6, 32.4, 32.0; HRMS (ESI-TOF): $[M+H]^+$ calculated for $C_{35}H_{29}N_6O_2$: 565.2352, found: 565.2345.

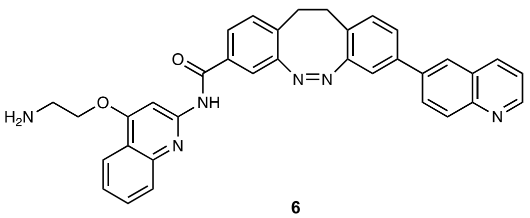

6

Compound 6: Prepared from diazocine **S3** (18.5 mg, 30.0 µmol) and 6-quinolineboronic acid pinacol ester (8.7 mg, 33.0 µmol) according to the general procedure. The TFA salt of the title compound **6** was obtained as a light orange solid (**6**·3TFA, 23.2 mg, 25.6 µmol, 85% yield over two steps): ^{1}H NMR (500 MHz, Methanol-*d*4) δ 9.01 (dd, *J* = 5.0, 2.0 Hz, 1H), 8.86 (d, *J* = 8.5 Hz, 1H), 8.44 (dd, *J* = 8.5, 1.0 Hz, 1H), 8.35 (d, *J* = 2.0 Hz, 1H), 8.23 (dd, *J* = 9.0, 2.0 Hz, 1H), 8.16 (d, *J* = 9.0 Hz, 1H), 8.00 (d, *J* = 8.5 Hz, 1H), 7.92 (ddd, *J* = 8.5, 7.0, 1.5 Hz, 1H), 7.87 – 7.81 (m, 2H), 7.71 – 7.65 (m, 2H), 7.54 (dd, *J* = 8.0, 2.0 Hz, 1H), 7.45 (s, 1H), 7.36 (d, *J* = 2.0 Hz, 1H), 7.34 (d, *J* = 8.0 Hz, 1H), 7.24 (d, *J* = 8.0 Hz, 1H), 4.65 (t, *J* = 5.0 Hz, 2H), 3.60 (t, *J* = 5.0 Hz, 2H), 3.09 – 2.94 (m, 4H); ^{13}C NMR (125 MHz, Methanol-*d*4) δ 169.0, 167.3, 157.4, 157.0, 153.2, 148.3, 144.2, 142.9, 141.1, 140.6, 139.2, 136.2, 134.7, 133.2, 132.4, 132.2, 131.9, 130.6, 129.9, 128.2, 128.1, 127.5, 127.2, 125.6, 124.2, 123.3, 122.9, 119.9, 119.8, 118.7, 94.9, 67.9, 39.6, 32.5, 32.0; HRMS (ESI-TOF): $[M+H]^+$ calculated for $C_{35}H_{29}N_6O_2$: 565.2352, found: 565.2357.

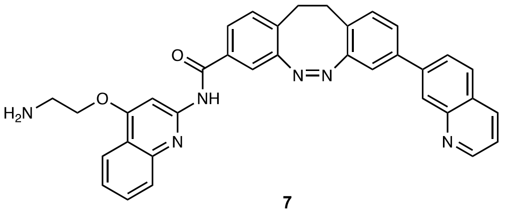

7

Compound 7: Prepared from diazocine **S3** (18.5 mg, 30.0 µmol) and quinoline-7-boronic acid pinacol ester (8.7 mg, 33.0 µmol) according to the general procedure. The TFA salt of the title compound **7** was obtained as a light yellow solid (**7**·3TFA, 22.2 mg, 24.5 µmol, 82% yield over two steps): ^{1}H NMR (500 MHz, Methanol-*d*4) δ 9.03 (dd, *J* = 5.0, 1.5 Hz, 1H), 8.79 (d, *J* = 8.0 Hz, 1H), 8.44 (dd, *J* = 8.5, 1.5 Hz, 1H), 8.26 (s, 1H), 8.19 (d, *J* = 8.5 Hz, 1H), 8.05 (dd, *J* = 9.0, 2.0 Hz, 1H), 7.99 (d, *J* = 8.5 Hz, 1H), 7.91 (ddd, *J* = 8.5, 7.0, 1.5 Hz, 1H), 7.84 (dd, *J* = 8.0, 1.9 Hz, 1H), 7.80 (dd, *J* = 8.5, 5.0 Hz, 1H), 7.69 – 7.64 (m, 2H), 7.58 (dd, *J* = 8.0, 2.0 Hz, 1H), 7.50 (s, 1H), 7.39 (d, *J* = 2.0 Hz, 1H), 7.37 (d, *J* = 8.0 Hz, 1H), 7.29 (d, *J* = 8.0 Hz, 1H), 4.65 (t, *J* = 5.0 Hz, 2H), 3.61 (t, *J* = 5.0 Hz, 2H), 3.13 – 2.97 (m, 4H); ^{13}C NMR (125 MHz, Methanol-*d*4) δ 168.9, 167.1, 157.4, 157.0, 153.2, 148.9, 145.2, 144.0, 143.6, 141.0, 139.1, 136.1, 134.6, 132.5, 132.3, 131.9, 130.7, 130.4, 129.6, 129.0, 128.2, 128.0, 127.6, 124.2, 123.1, 122.8, 122.1, 119.9, 119.8, 118.8, 94.9, 67.8, 39.6, 32.4, 32.0; HRMS (ESI-TOF): $[M+H]^+$ calculated for $C_{35}H_{29}N_6O_2$: 565.2352, found: 565.2346.

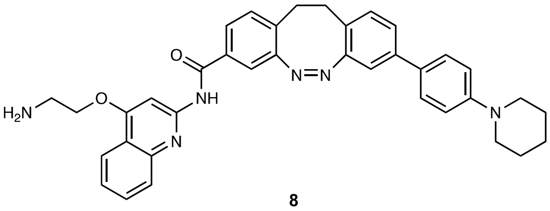

8

Compound 8: Prepared from diazocine **S3** (18.5 mg, 30.0 µmol) and 4-(1-piperidinyl)benzeneboronic acid pinacol ester (10.0 mg, 33.0 mmol) according to the general procedure. The TFA salt of the title compound **8** was obtained as a light orange solid (**8**·3TFA,

21.3 mg, 22.7 µmol, 76% yield over two steps): ^{1}H NMR (400 MHz, Methanol-d_4) δ 8.44 (d, J = 8.4 Hz, 1H), 8.00 (d, J = 8.4 Hz, 1H), 7.95 – 7.89 (m, 1H), 7.83 (dd, J = 8.0, 2.0 Hz, 1H), 7.74 – 7.65 (m, 3H), 7.63 (d, J = 2.0 Hz, 1H), 7.59 (d, J = 8.4 Hz, 2H), 7.50 (s, 1H), 7.37 (dd, J = 8.0, 2.0 Hz, 1H), 7.32 (d, J = 8.0 Hz, 1H), 7.20 – 7.15 (m, 2H), 4.66 (t, J = 4.8 Hz, 2H), 3.60 (t, J = 4.8 Hz, 2H), 3.55 (t, J = 5.6 Hz, 4H), 3.07 – 2.91 (m, 4H), 2.03 – 1.93 (m, 4H), 1.79 – 1.71 (m, 2H); ^{13}C NMR (100 MHz, Methanol-d_4) δ 169.0, 167.4, 157.2, 156.9, 153.2, 144.1, 141.7, 140.4, 139.4, 136.2, 134.8, 132.3, 132.0, 131.8, 129.7, 129.3, 128.2, 128.1, 127.1, 124.3, 122.7, 122.2, 120.0, 119.7, 118.1, 94.9, 68.0, 57.6, 39.6, 32.5, 32.0, 25.1, 22.4; HRMS (ESI-TOF): [M+H]$^+$ calculated for $C_{33}H_{37}N_6O_2$: 597.2978, found: 597.2997.

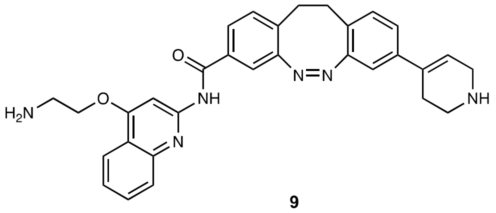

9

Compound 9: Prepared from diazocine **S3** (18.5 mg, 30.0 µmol) and *N*-Boc-1,2,3,6-tetrahydropyridine-4-boronic acid pinacol ester (10.8 mg, 33.0 µmol) according to the general procedure. The TFA salt of the title compound **9** was obtained as a light yellow solid (**9**·3TFA, 21.7 mg, 25.2 µmol, 84% yield over two steps): δ ^{1}H NMR (500 MHz, Methanol-d_4) δ 8.43 (dd, J = 8.5, 1.0 Hz, 1H), 7.97 (d, J = 8.5 Hz, 1H), 7.88 (ddd, J = 8.5, 7.0, 1.5 Hz, 1H), 7.81 (dd, J = 8.0, 2.0 Hz, 1H), 7.71 (s, 1H), 7.64 (ddd, J = 8.5, 7.0, 1.5 Hz, 1H), 7.58 (d, J = 2.0 Hz, 1H), 7.32 (d, J = 8.0 Hz, 1H), 7.21 (dd, J = 8.0, 2.0 Hz, 1H), 7.11 (d, J = 8.0 Hz, 1H), 7.01 (d, J = 2.0 Hz, 1H), 6.15 – 6.11 (m, 1H), 4.65 (t, J = 5.0 Hz, 2H), 3.82 – 3.77 (m, 2H), 3.63 (t, J = 5.0 Hz, 2H), 3.41 (t, J = 6.0 Hz, 2H), 3.08 – 2.90 (m, 4H), 2.75 – 2.69 (m, 2H); ^{13}C NMR (125 MHz, Methanol-d_4) δ 169.0, 167.7, 156.9, 156.7, 153.1, 140.1, 139.3, 136.3, 135.7, 134.9, 132.1, 131.8, 131.5, 129.2, 128.23, 128.21, 125.2, 124.4, 122.5, 120.0, 119.7, 117.9, 116.4, 94.9, 68.1, 43.3, 42.0, 39.6, 32.4, 31.9, 24.5; HRMS (ESI-TOF): [M+H]$^+$ calculated for $C_{31}H_{31}N_6O_2$: 519.2508, found: 519.2507.

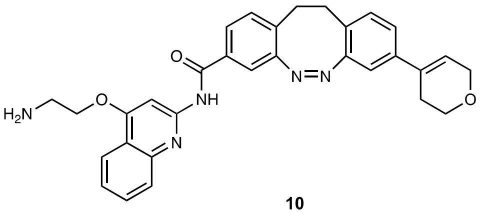

10

Compound 10: Prepared from diazocine **S3** (18.5 mg, 30.0 µmol) and 3,6-Dihydro-2*H*-pyran-4-boronic acid pinacol ester (7.0 mg, 33.0 µmol) according to the general procedure. The TFA salt of the title compound **10** was obtained as a light orange solid (**10**·2TFA, 16.7 mg, 22.3 µmol, 75% yield over two steps): ^{1}H NMR (500 MHz, Methanol-d_4) δ 8.47 (dd, J = 8.5, 1.0 Hz, 1H), 8.03 (d, J = 8.5 Hz, 1H), 7.95 (ddd, J = 8.5, 7.0, 1.0 Hz, 1H), 7.82 (dd, J = 8.0, 2.0 Hz, 1H), 7.70 (ddd, J = 8.5, 7.0, 1.0 Hz, 1H), 7.60 (d, J = 2.0 Hz, 1H), 7.50 (s, 1H), 7.31 (d, J = 8.0 Hz, 1H), 7.13 (dd, J = 8.0, 2.0 Hz, 1H), 7.03 (d, J = 8.0 Hz, 1H), 6.92 (d, J = 1.5 Hz, 1H), 6.15 – 6.10 (m, 1H), 4.69 (t, J = 5.0 Hz, 2H), 4.23 – 4.18 (m, 2H), 3.83 (t, J = 5.5 Hz, 2H), 3.63 (t, J = 5.0 Hz, 2H), 3.03 – 2.84 (m, 4H), 2.45 – 2.32 (m, 2H); ^{13}C NMR (100 MHz, Methanol-d_4) δ 169.3, 168.1, 157.0, 156.7, 153.1, 140.5, 139.1, 136.7, 135.3, 134.0, 131.9, 131.8, 131.3, 128.5, 128.2, 128.1, 124.8, 124.5, 124.2, 121.9, 119.9, 119.6, 115.8, 94.7, 68.3, 66.7, 65.4, 39.6, 32.5, 32.0, 27.8; HRMS (ESI-TOF): [M+H]$^+$ calculated for $C_{31}H_{30}N_5O_3$: 520.2349, found: 520.2344.

Synthesis of G4switch-biotin

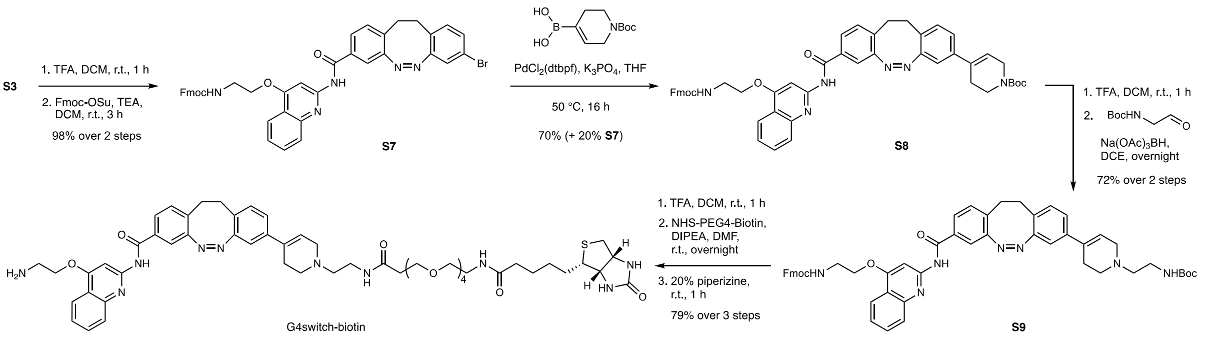

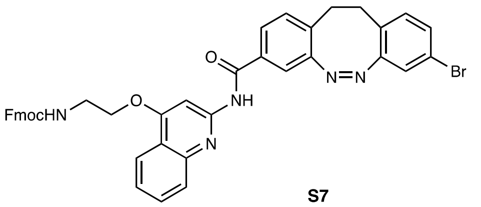

S7

Compound S7: To a solution of **S3** (40.8 mg, 66.2 μmol) in CH_2Cl_2 (2.0 ml) was added TFA (0.5 ml). The mixture was stirred at room temperature for 1 h. The solvents were removed in vacuo to afford the crude product, the total amount of which was used without further purification. The crude amine product was redissolved in CH_2Cl_2 (2.0 ml), and triethylamine (46.1 μl, 331 μmol) and Fmoc-OSu (67.0 mg, 199 μmol) were added into the solution. The reaction mixture was stirred at room temperature for 3 h. The reaction was purified by flash silica column chromatography (0 ~ 50% EtOAc in petroleum ether) to give the title compound **S7** as a yellow solid (47.8 mg, 64.7 μmol, 98% yield over two steps): ^1H NMR (700 MHz, DMSO-d_6) δ 11.05 (s, 1H), 8.18 (d, J = 8.4 Hz, 1H), 7.89 – 7.75 (m, 5H), 7.73 – 7.66 (m, 2H), 7.65 (d, J = 7.7 Hz, 2H), 7.59 (s, 1H), 7.45 (t, J = 7.7 Hz, 1H), 7.35 (t, J = 7.7 Hz, 2H), 7.29 – 7.20 (m, 4H), 7.15 (d, J = 1.4 Hz, 1H), 7.10 (d, J = 7.7 Hz, 1H), 4.35 (d, J = 7.0 Hz, 2H), 4.24 (t, J = 4.9 Hz, 2H), 4.21 (t, J = 7.0 Hz, 1H), 3.56 (q, J = 4.9 Hz, 2H), 2.96 – 2.77 (m, 4H); ^{13}C NMR (175 MHz, DMSO-d_6) δ 165.3, 161.9, 156.4, 156.0, 154.5, 152.9, 147.0, 143.8, 140.7, 132.6, 132.2, 131.9, 130.4, 130.2, 130.1, 127.6, 127.5, 127.1, 127.0, 126.8, 125.1, 124.3, 122.0, 121.0, 120.1, 119.4, 119.1, 118.5, 95.0, 67.3, 65.4, 46.7, 39.7, 30.8, 30.0; HRMS (ESI-TOF): $[M+H]^+$ calculated for $C_{41}H_{33}N_5O_4Br$: 738.1716, found: 738.1702.

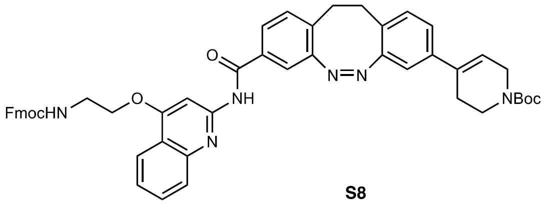

S8

Compound S8: To a solution of **S7** (27.3 mg, 37.0 μmol) and *N*-Boc-1,2,3,6-tetrahydropyridine-4-boronic acid (16.8 mg, 74.0 μmol) in anhydrous THF (3.0 ml) under argon atmosphere was added [1,1′-Bis(di-tert-butylphosphino)ferrocene]dichloropalladium(II) ($PdCl_2$(dtbpf)) (7.3 mg, 11.2 μmol) and anhydrous K_3PO_4 (23.6 mg, 111 μmol). The reaction mixture was degassed by bubbling argon into the flask and stirred at 50 °C under argon atmosphere for 24 h. After being cooled down, the reaction was diluted in 10 ml H_2O. The resulting solution was extracted with EtOAc (15 ml × 3), and the combined organic layers were washed with brine (5 ml), dried over anhydrous $MgSO_4$ and filtered. The solvents were removed in vacuo and the residue was purified by flash silica column chromatography (0 ~ 50% EtOAc in petroleum ether) to give the title compound **S8** as a yellow solid (21.8 mg, 25.9 μmol, 70% yield, 20% **S7** recovered): ^1H NMR (500 MHz, DMSO-d_6) δ 11.02 (s, 1H), 8.17 (d, J = 8.0 Hz, 1H), 7.88 – 7.75 (m, 5H), 7.73 – 7.61 (m, 4H),

7.56 (d, *J* = 1.0 Hz, 1H), 7.44 (ddd, *J* = 8.0, 7.0, 1.0 Hz, 1H), 7.35 (t, *J* = 7.5 Hz, 2H), 7.28 – 7.20 (m, 3H), 7.15 (dd, *J* = 8.0, 1.5 Hz, 1H), 7.08 (d, *J* = 8.0 Hz, 1H), 6.96 (d, *J* = 1.5 Hz, 1H), 6.14 (s, 1H), 4.34 (d, *J* = 7.0 Hz, 2H), 4.26 – 4.16 (m, 3H) 3.92 (s, 2H), 3.55 (q, *J* = 5.0 Hz, 2H), 3.45 (q, *J* = 6.0 Hz, 2H), 2.96 – 2.80 (m, 4H), 2.43 – 2.27 (m, 2H), 1.37 (s, 9H);^{13}C NMR (125 MHz, DMSO-d_6) δ 165.3, 161.8, 156.4, 155.0, 154.7, 153.8, 152.9, 147.0, 143.8, 140.7, 138.6, 133.1, 132.6, 132.5, 130.4, 130.0, 127.6, 127.0, 126.82, 126.76, 126.6, 125.1, 124.2, 123.5, 122.0, 121.8 (br), 120.1, 119.1, 118.5, 114.5, 95.1, 78.8, 67.3, 65.4, 46.7, 43.2 (br), 30.9, 30.4, 28.1, 26.2 (br); HRMS (ESI-TOF): $[M+H]^+$ calculated for $C_{51}H_{49}N_6O_6$: 841.3714, found: 841.3707.

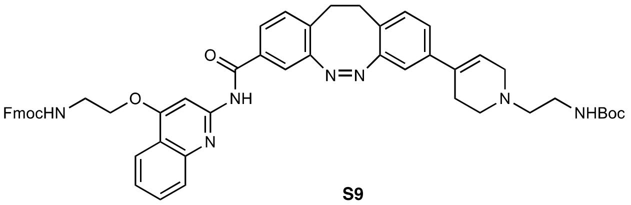

S9

Compound S9: To a solution of **S8** (21.8 mg, 25.9 μmol) in CH_2Cl_2 (2.0 ml) was added TFA (0.5 ml). The mixture was stirred at room temperature for 1 h. The solvents were removed in vacuo to afford the crude product, the total amount of which was used without further purification. The crude amine product was redissolved in 1,2-dichloroethane (3 ml), and *N*-Boc-2-aminoacetaldehyde (8.3 mg, 52.1 μmol) and sodium triacetoxyborohydride (16.5 mg, 77.9 μmol) were then added into the solution. The reaction mixture was stirred at room temperature overnight. The reaction was then quenched by 5% $NaHCO_3$ aqueous solution (5 ml), and the resulting solution was extracted with EtOAc (15 ml × 3). The combined organic layers were washed with H_2O (5 ml × 2) and brine (5 ml), dried over anhydrous $MgSO_4$ and filtered. The solvent was removed in vacuo and the residue was purified by flash silica column chromatography (0 ~ 5% methanol in CH_2Cl_2) to give the title compound **S9** as a yellow solid (16.6 mg, 18.8 μmol, 72% yield over two steps): ^{1}H NMR (500 MHz, DMSO-d_6) δ 11.01 (s, 1H), 8.17 (dd, *J* = 8.0, 1.5 Hz, 1H), 7.86 – 7.76 (m, 5H), 7.72 – 7.61 (m, 4H), 7.56 (d, *J* = 1.5 Hz, 1H), 7.44 (ddd, *J* = 8.0, 7.5, 1.0 Hz, 1H), 7.35 (t, *J* = 7.5 Hz, 2H), 7.27 – 7.20 (m, 3H), 7.13 (dd, *J* = 8.0, 1.5 Hz, 1H), 7.06 (d, *J* = 8.0 Hz, 1H), 6.93 (d, *J* = 1.5 Hz, 1H), 6.61 (t, *J* = 5.5 Hz, 1H), 6.13 (s, 1H), 4.34 (d, *J* = 6.5 Hz, 2H), 4.26 – 4.16 (m, 3H), 3.55 (q, *J* = 5.0 Hz, 2H), 3.06 – 2.98 (m, 4H), 2.93 – 2.81 (m, 4H), 2.55 (t, *J* = 5.5 Hz, 2H), 2.42 – 2.28 (m, 4H), 1.31 (s, 9H); ^{13}C NMR (125 MHz, DMSO-d_6) δ 165.3, 161.9, 156.4, 155.5, 155.0, 154.7, 152.9, 147.0, 143.8, 140.7, 138.7, 132.7, 132.6, 132.4, 130.4, 130.02, 129.98, 127.6, 127.0, 126.8, 126.3, 125.1, 124.3, 123.4, 122.9, 122.0, 120.1, 119.1, 118.5, 114.3, 95.1, 77.5, 67.3, 65.4, 56.9, 52.6, 49.7, 46.7, 39.7, 37.6, 30.9, 30.4, 28.2, 27.0; HRMS (ESI-TOF): $[M+H]^+$ calculated for $C_{53}H_{54}N_7O_6$: 884.4136, found: 884.4145.

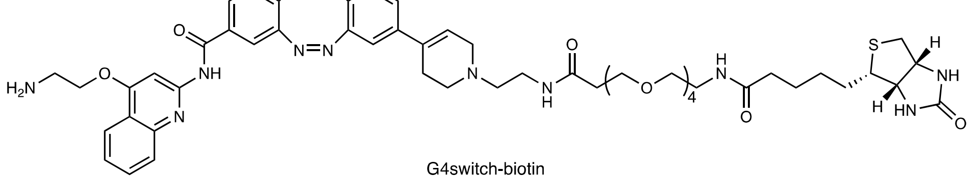

G4switch-biotin

G4switch-biotin: To a solution of **S9** (16.6 mg, 18.8 μmol) in CH_2Cl_2 (2.0 ml) was added TFA (0.5 ml). The mixture was stirred at room temperature for 1 h. The solvents were removed in vacuo to afford the crude product, the total amount of which was used without further purification. The crude amine product was redissolved in DMF (2.0 ml), followed by adding *N,N*-diisopropylethylamine (16.3 μl, 93.8 μmol) and Biotin-PEG4-

NHS (13.3 mg, 22.6 µmol) into the solution. The reaction mixture was stirred at room temperature overnight. 0.5 ml piperidine was then added into the solution. The mixture was stirred at room temperature for another 1 h. The solvents were removed in vacuo and the resulting residue was purified by reversed-phase flash chromatography with 5% ~ 100% acetonitrile (containing 0.1% TFA) in H_2O (containing 0.1% TFA) at a flow rate of 20 ml/min over 30 min. Most of the solvents were removed in vacuo, and the rest of solvents were removed by freeze-drying to give a TFA salt of the title product as a light yellow solid (**G4switch-biotin**·3TFA, 20.5 mg, 14.9 µmol, 79% yield over three steps): ^{1}H NMR (500 MHz, Methanol-d_4) δ 8.50 (dd, *J* = 8.5, 1.0 Hz, 1H), 8.09 (dd, *J* = 8.5, 1.0 Hz, 1H), 8.00 (ddd, *J* = 8.5, 7.0, 1.0 Hz, 1H), 7.86 (dd, *J* = 8.0, 2.0 Hz, 1H), 7.75 (ddd, *J* = 8.5, 7.0, 1.0 Hz, 1H), 7.64 (d, *J* = 2.0 Hz, 1H), 7.48 (s, 1H), 7.33 (d, *J* = 8.0 Hz, 1H), 7.20 (dd, *J* = 8.0, 2.0 Hz, 1H), 7.10 (d, *J* = 8.0 Hz, 1H), 7.01 (d, *J* = 2.0 Hz, 1H), 6.10 (s, 1H), 4.72 (t, *J* = 5.0 Hz, 2H), 4.45 (dd, *J* = 7.5, 5.0 Hz, 1H), 4.26 (dd, *J* = 8.0, 4.5 Hz, 1H), 4.11 (br, 1H), 3.82 (br, 2H), 3.69 (t, *J* = 6.0 Hz, 2H), 3.66 – 3.54 (m, 16H), 3.49 (t, *J* = 5.5 Hz, 2H), 3.37 – 3.31 (m, 4H), 3.19 – 3.14 (m, 1H), 3.04 – 2.86 (m, 5H), 2.79 (br, 2H), 2.66 (dd, *J* = 13.0, 2.5 Hz, 1H), 2.45 (t, *J* = 6.0 Hz, 2H), 2.17 (t, *J* = 7.5 Hz, 2H), 1.72 – 1.49 (m, 4H), 1.43 – 1.34 (m, 1H); ^{13}C NMR (175 MHz, Methanol-d_4) δ 176.1, 175.6, 169.3, 168.4, 166.1, 156.9, 156.8, 153.0, 138.7, 138.6, 136.6, 135.5, 131.9, 131.8, 131.6, 129.4, 128.7, 128.3, 125.3, 124.6, 121.6, 120.2, 119.6, 117.4, 116.5, 94.7, 71.51, 71.49, 71.43, 71.35, 71.3, 71.2, 70.5, 68.4, 67.9, 63.4, 61.6, 57.03, 57.00, 52.2, 50.5, 41.1, 40.3, 39.6, 37.3, 36.7, 35.7, 32.5, 32.0, 29.7, 29.5, 26.9, 25.2; HRMS (ESI-TOF): $[M+H]^+$ calculated for $C_{54}H_{71}N_{10}O_9S$: 1035.5126, found: 1035.5168.

NMR spectra

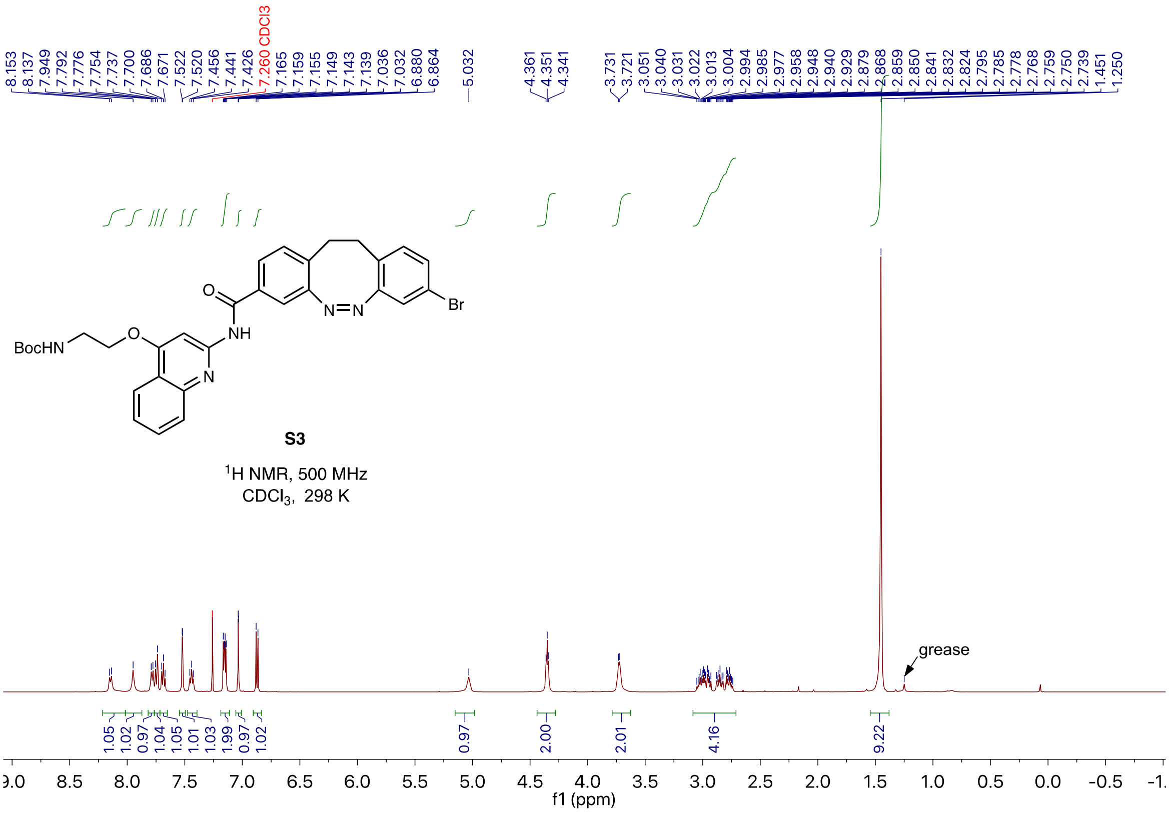

S3
1H NMR, 500 MHz
CDCl3, 298 K
grease
f1 (ppm)

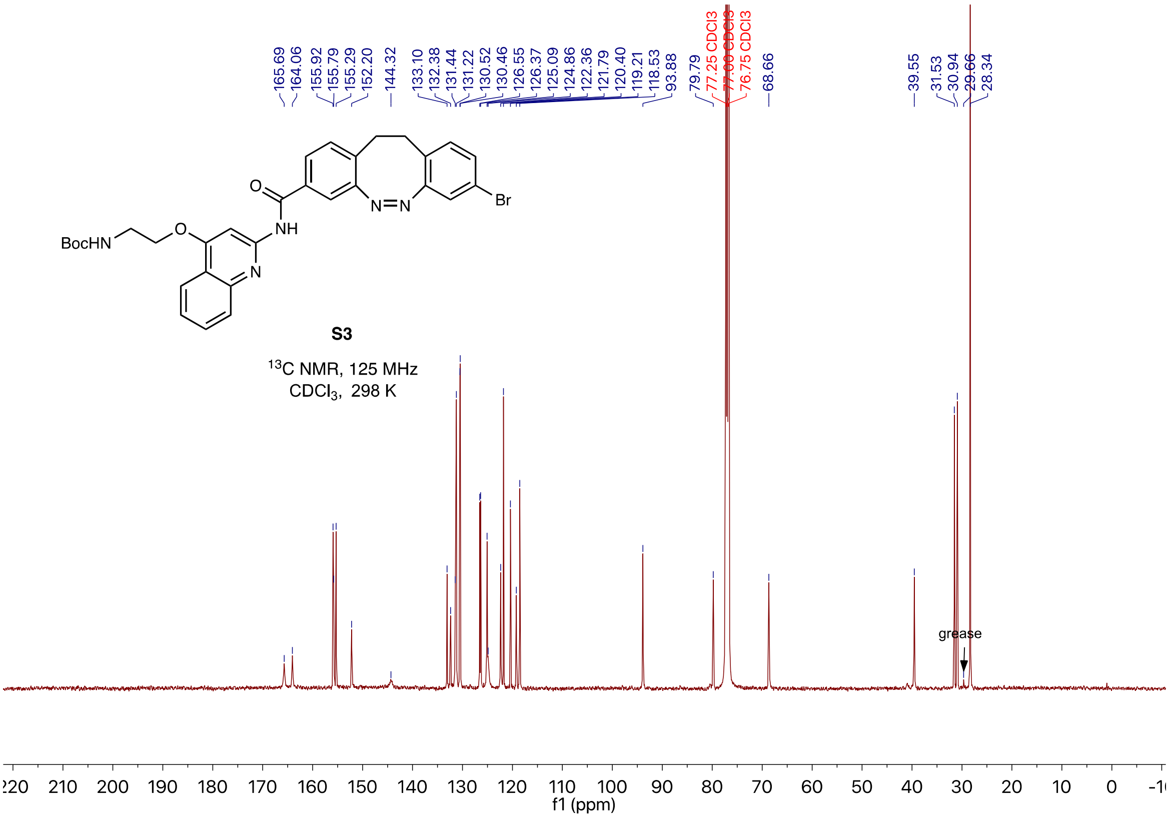

S3
13C NMR, 125 MHz
CDCl3, 298 K
grease
f1 (ppm)

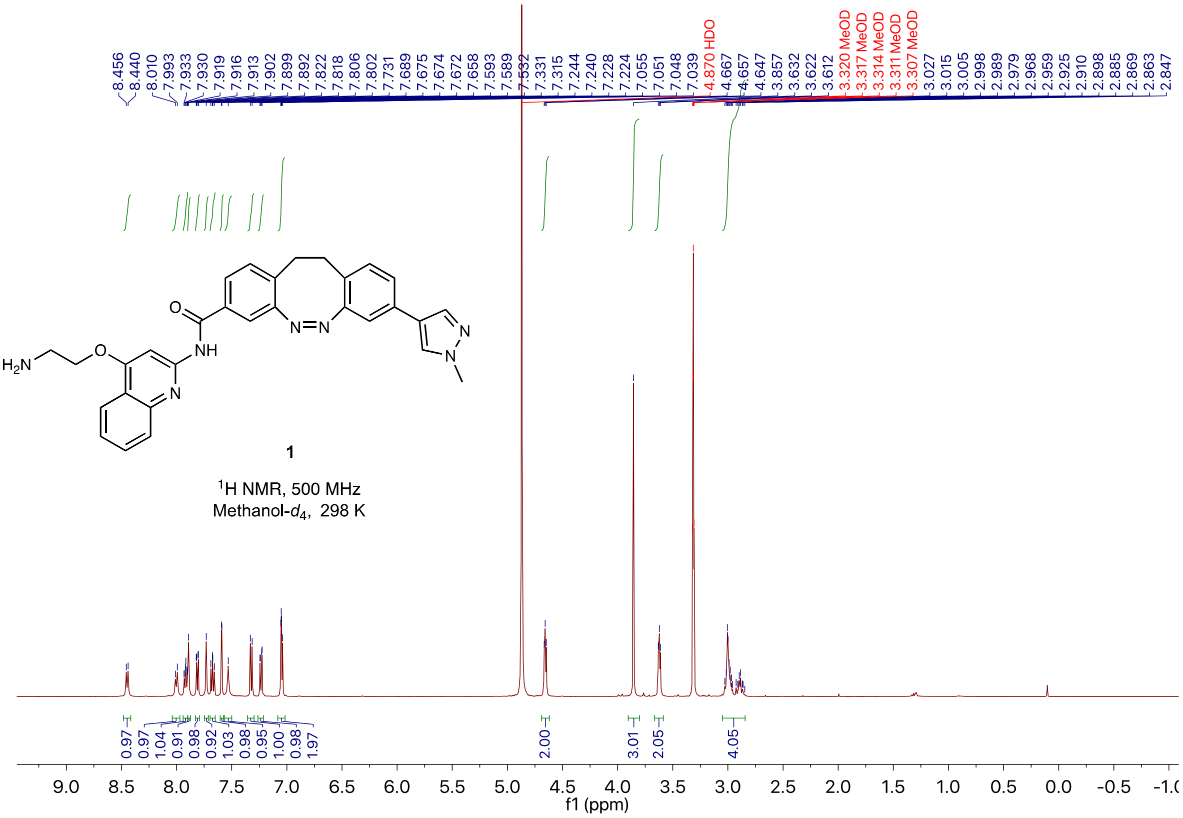
8.456
8.440
8.010
7.993
7.933
7.930
7.919
7.916
7.913
7.902
7.899
7.892
7.822
7.818
7.806
7.802
7.731
7.689
7.675
7.674
7.672
7.658
7.593
7.589
7.532
7.331
7.315
7.244
7.240
7.228
7.224
7.055
7.051
7.048
7.039
4.870 HDO
4.667
4.657
4.647
3.857
3.632
3.622
3.612
3.320 MeOD
3.317 MeOD
3.314 MeOD
3.311 MeOD
3.307 MeOD
3.027
3.015
3.005
2.998
2.989
2.979
2.968
2.959
2.925
2.910
2.898
2.885
2.869
2.863
2.847
O
NH
H2N
O
N
N=N
N
N
1
1H NMR, 500 MHz
Methanol-d4, 298 K
0.97
0.97
1.04
0.91
0.98
0.92
1.03
0.98
0.95
1.00
0.98
1.97
2.00
3.01
2.05
4.05
9.0 8.5 8.0 7.5 7.0 6.5 6.0 5.5 5.0 4.5 4.0 3.5 3.0 2.5 2.0 1.5 1.0 0.5 0.0 -0.5
f1 (ppm)

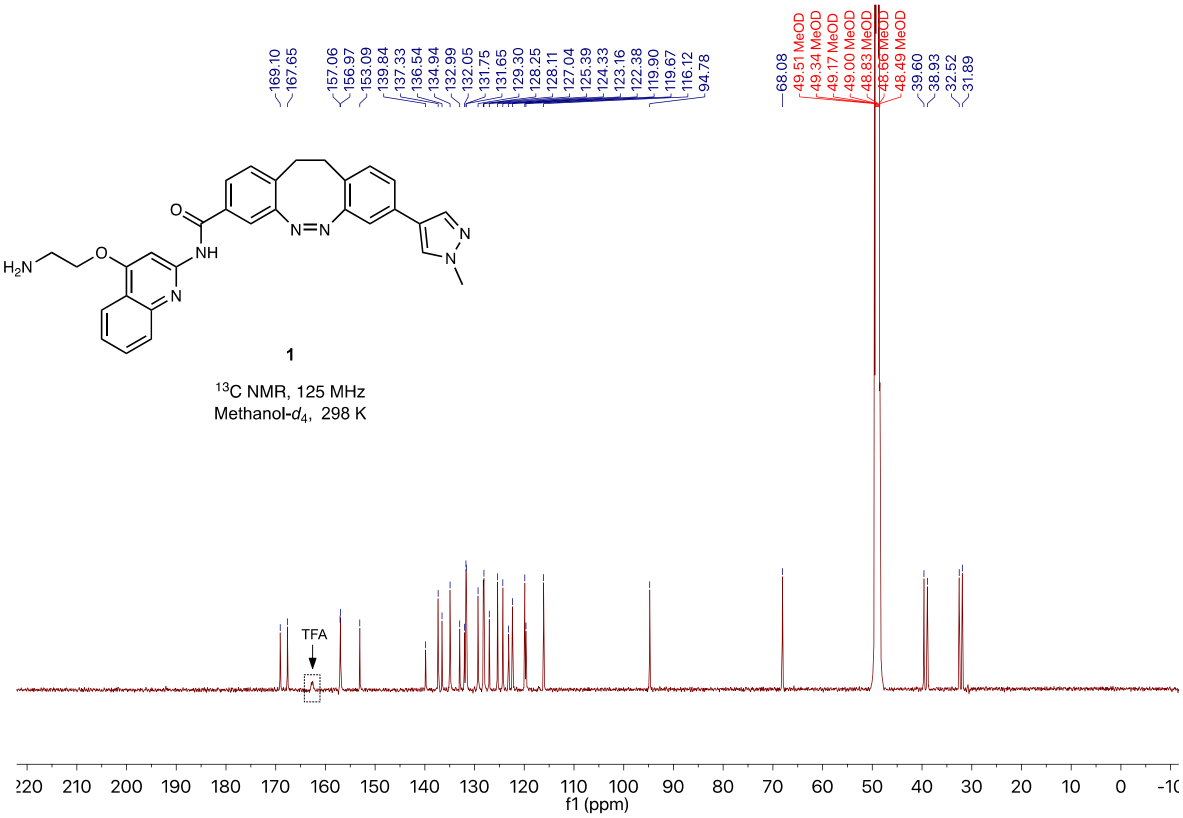
169.10
167.65
157.06
156.97
153.09
139.84
137.33
136.54
134.94
132.99
132.05
131.75
131.65
129.30
128.25
128.11
127.04
125.39
124.33
123.16
122.38
119.90
119.67
116.12
94.78
68.08
49.51 MeOD
49.34 MeOD
49.17 MeOD
49.00 MeOD
48.83 MeOD
48.66 MeOD
48.49 MeOD
39.60
38.93
32.52
31.89
O
NH
H2N
O
N
N=N
N
N
1
13C NMR, 125 MHz
Methanol-d4, 298 K
TFA
210 200 190 180 170 160 150 140 130 120 110 100 90 80 70 60 50 40 30 20 10 0
f1 (ppm)

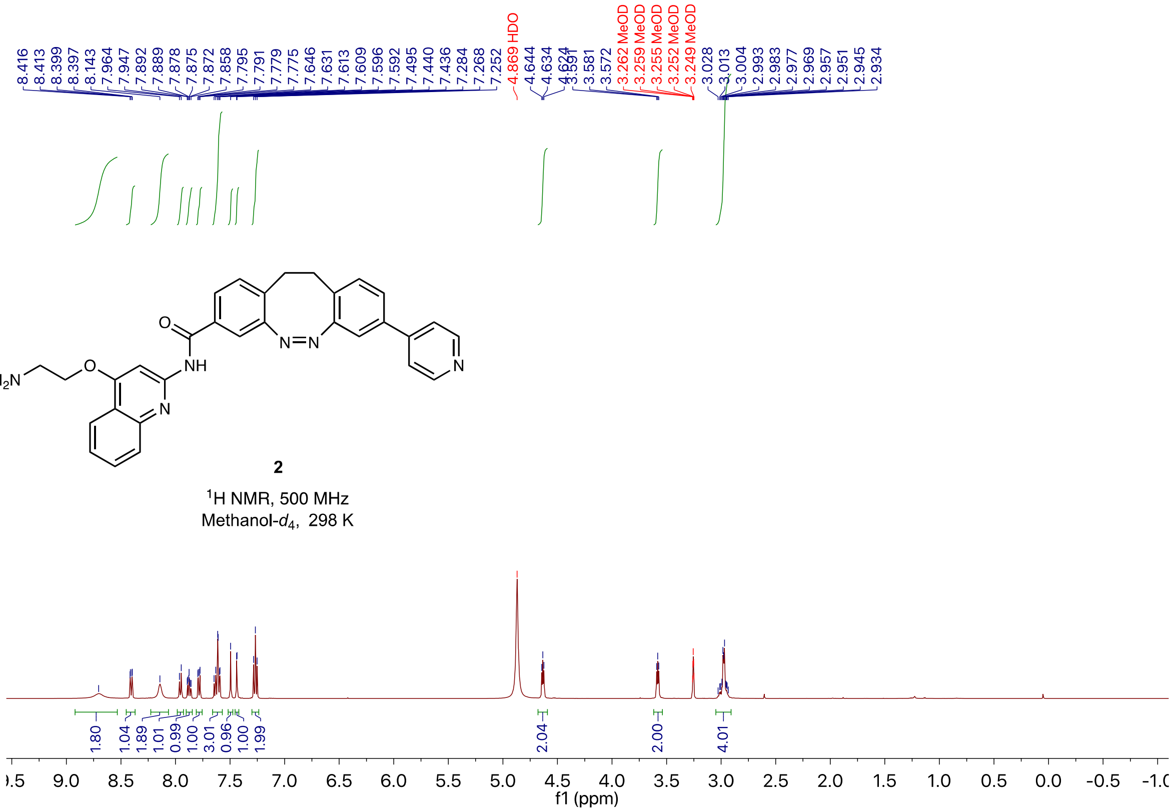
8.416 8.413 8.399 8.397 8.143 7.964 7.947 7.892 7.889 7.878 7.875 7.872 7.858 7.795 7.791 7.779 7.775 7.646 7.631 7.613 7.609 7.596 7.592 7.495 7.440 7.436 7.284 7.268 7.252
4.869 HDO
4.644 4.634 4.624
3.591 3.581 3.572
3.262 MeOD 3.259 MeOD 3.255 MeOD 3.252 MeOD 3.249 MeOD
3.028 3.013 3.004 2.993 2.983 2.977 2.969 2.957 2.951 2.945 2.934
O
NH
N=N
N
H2N
O
N
2
1H NMR, 500 MHz
Methanol-d4, 298 K
1.80 1.04 1.89 1.01 0.99 1.00 3.01 0.96 1.00 1.99
2.04
2.00
4.01
9.5 9.0 8.5 8.0 7.5 7.0 6.5 6.0 5.5 5.0 4.5 4.0 3.5 3.0 2.5 2.0 1.5 1.0 0.5 0.0 -0.5 -1.0
f1 (ppm)

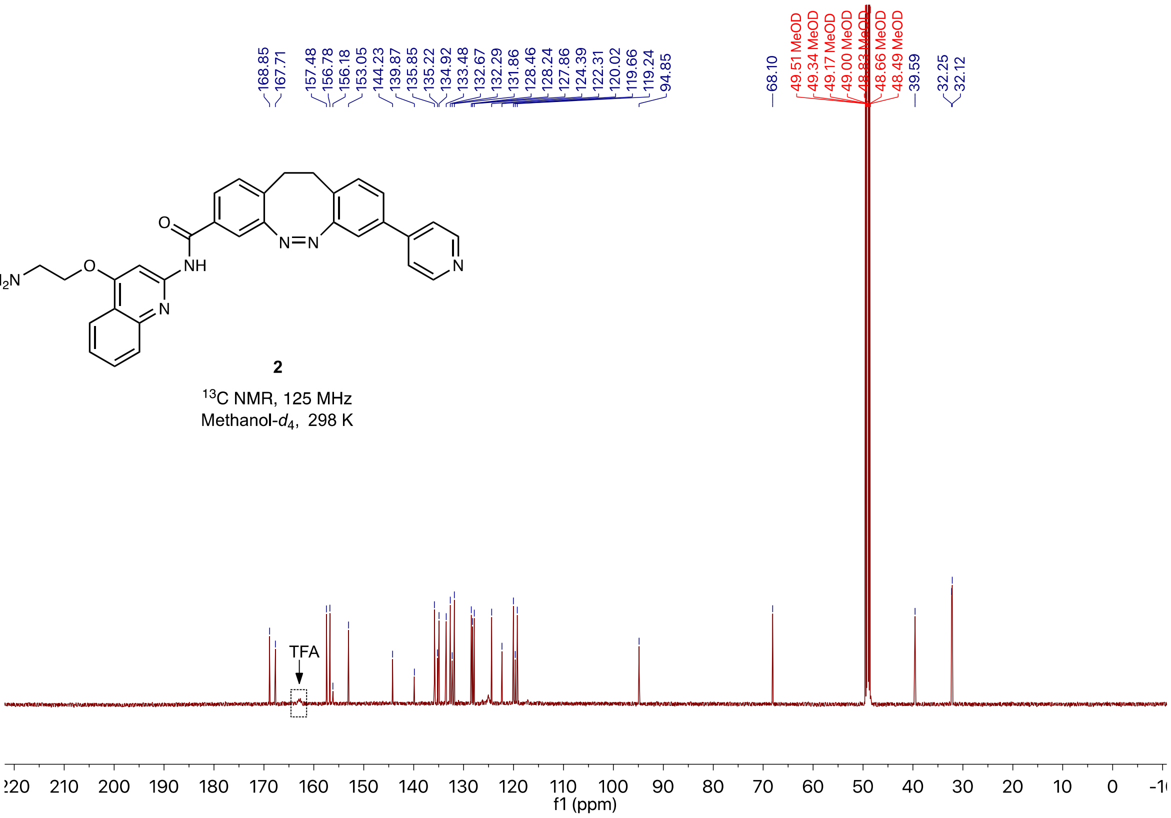
168.85 167.71 157.48 156.78 156.18 153.05 144.23 139.87 135.85 135.22 134.92 133.48 132.67 132.29 131.86 128.46 128.24 127.86 124.39 122.31 120.02 119.66 119.24 94.85
68.10
49.51 MeOD 49.34 MeOD 49.17 MeOD 49.00 MeOD 48.83 MeOD 48.66 MeOD 48.49 MeOD
39.59
32.25 32.12
O
NH
N=N
N
H2N
O
N
2
13C NMR, 125 MHz
Methanol-d4, 298 K
TFA
220 210 200 190 180 170 160 150 140 130 120 110 100 90 80 70 60 50 40 30 20 10 0 -10
f1 (ppm)

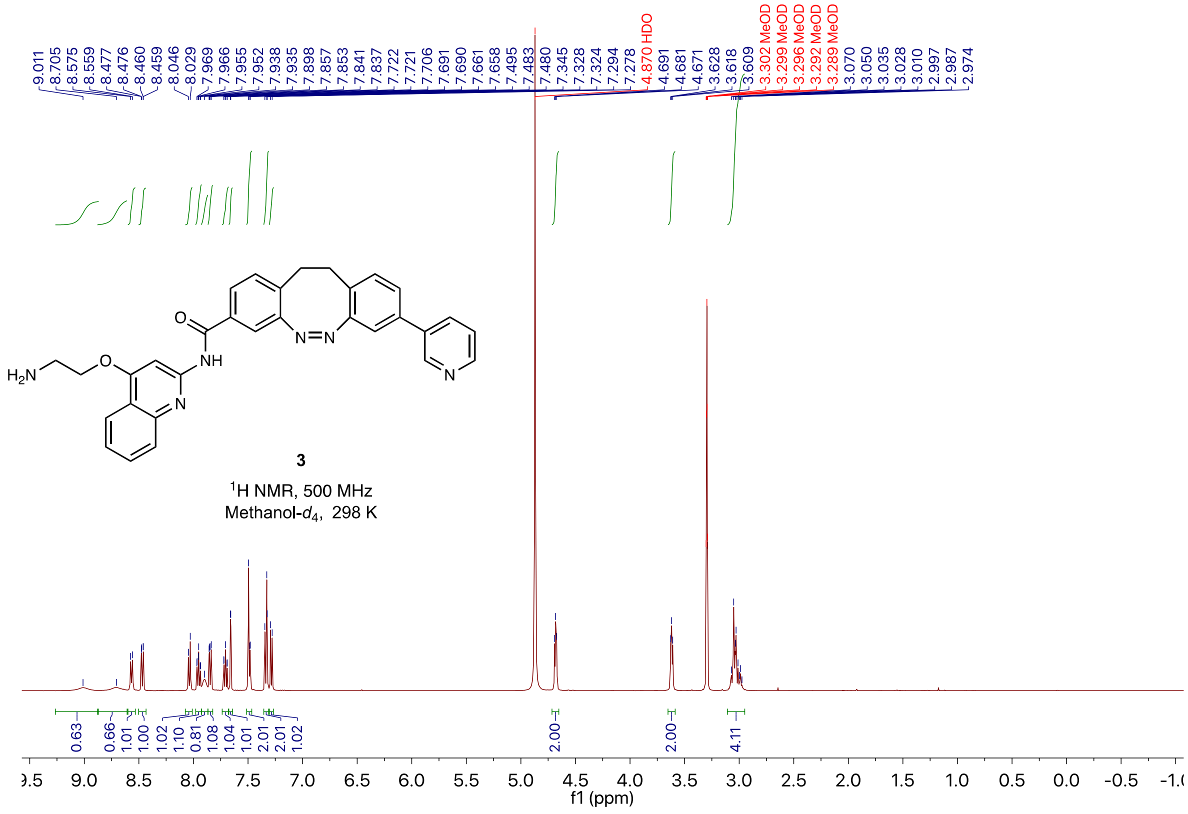

3
^{1}H NMR, 500 MHz
Methanol-d_4, 298 K
f1 (ppm)

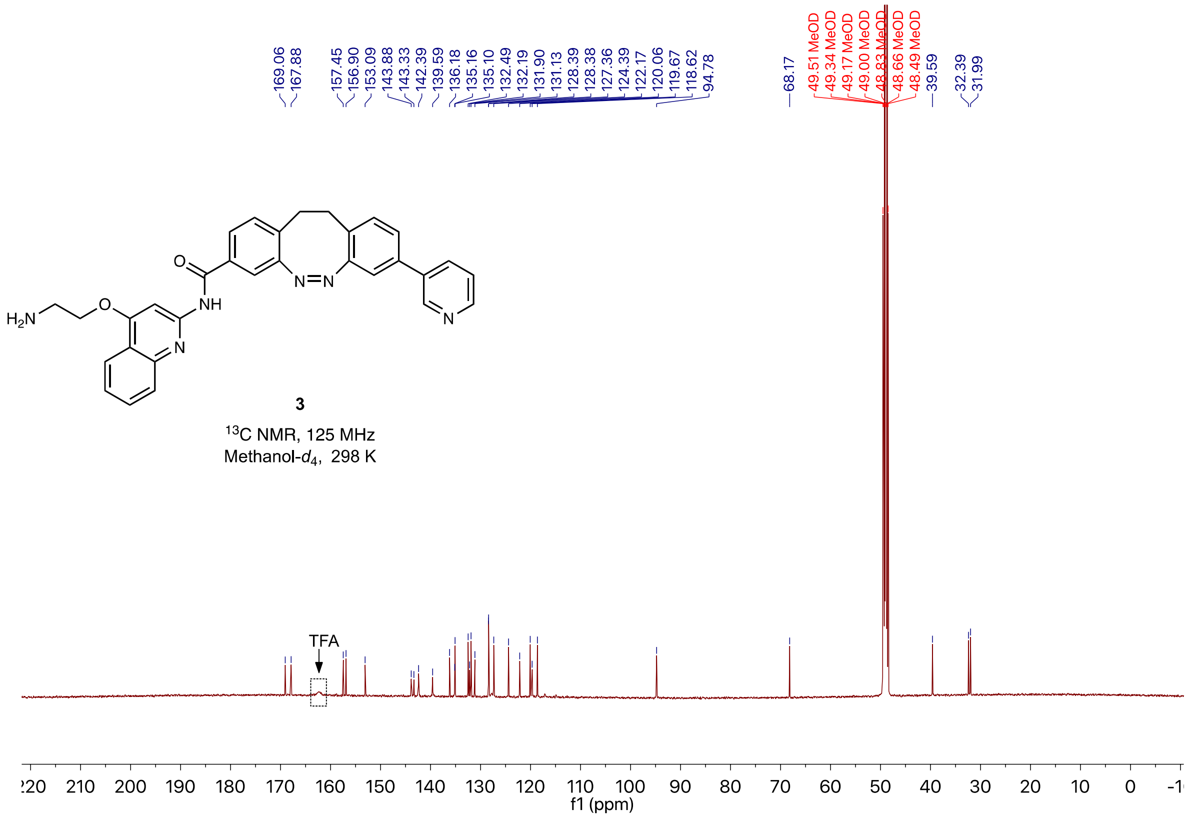

TFA
3
^{13}C NMR, 125 MHz
Methanol-d_4, 298 K
f1 (ppm)

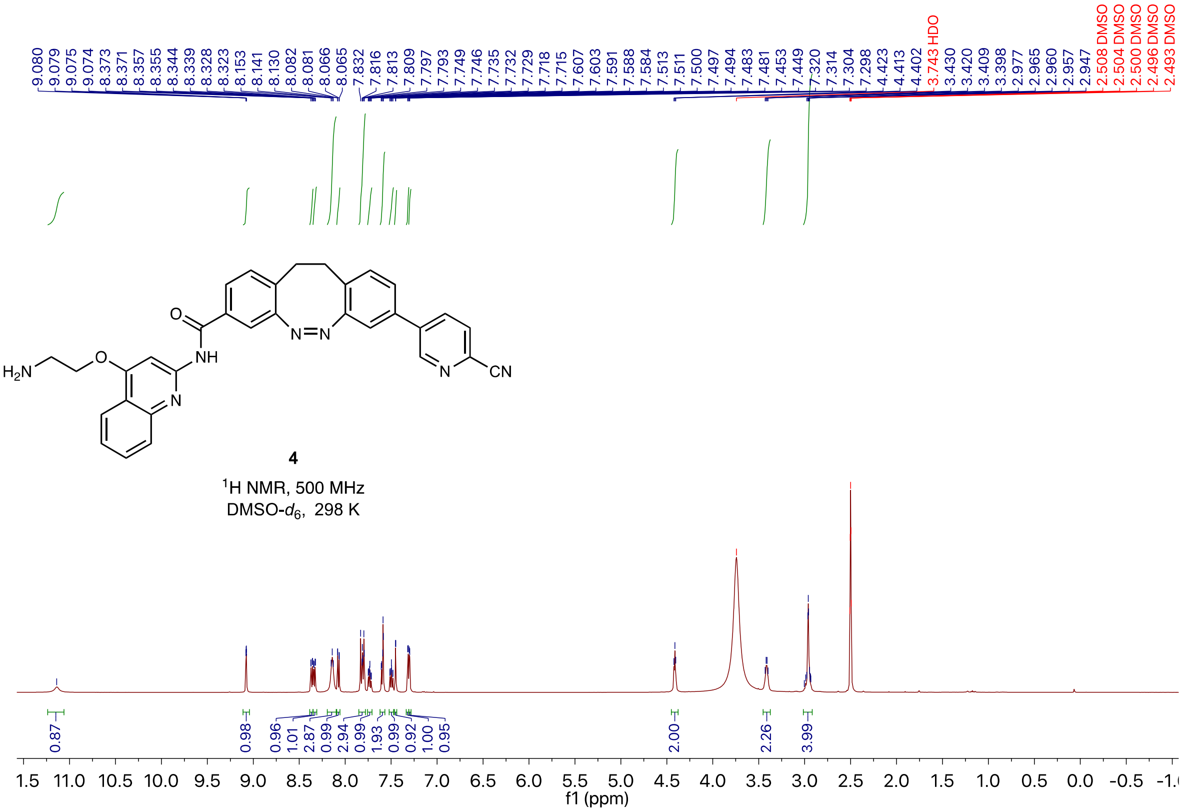
4
1H NMR, 500 MHz
DMSO-d6, 298 K
f1 (ppm)

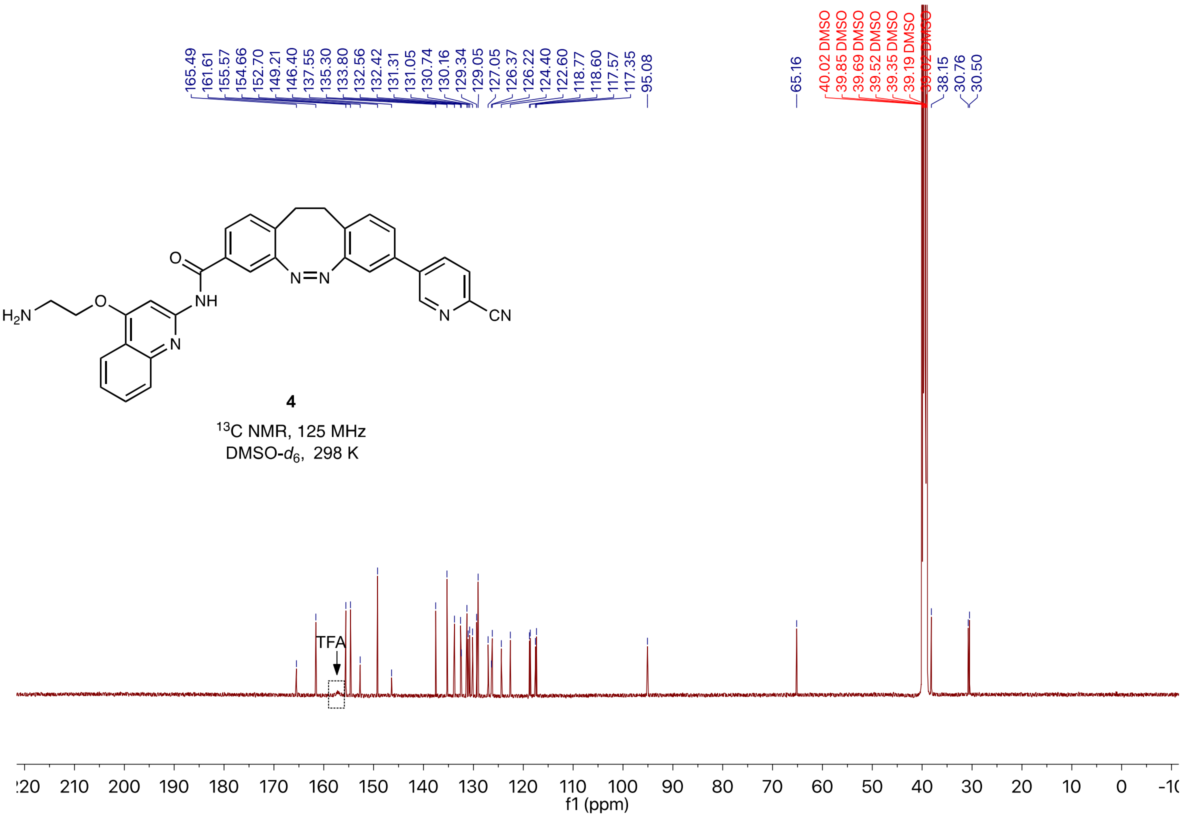
4
13C NMR, 125 MHz
DMSO-d6, 298 K
TFA
f1 (ppm)

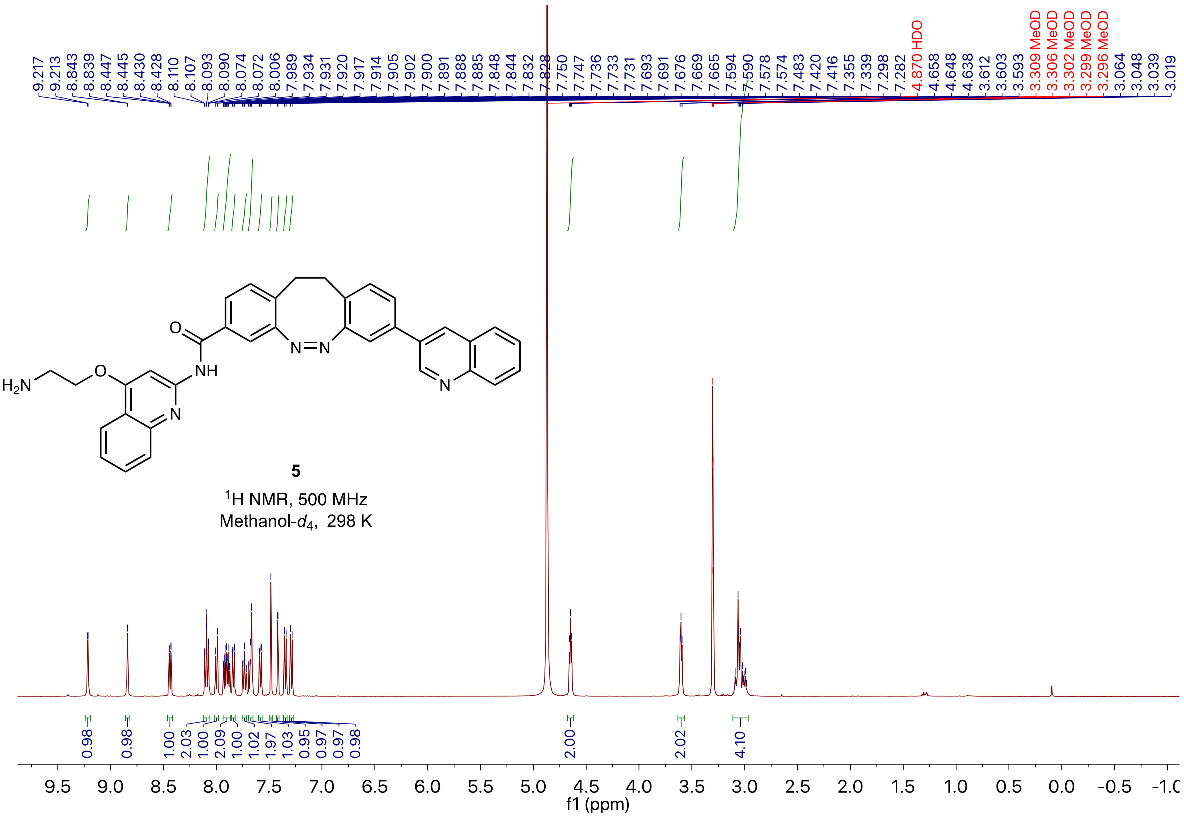

5

^{1}H NMR, 500 MHz

Methanol-d_4, 298 K

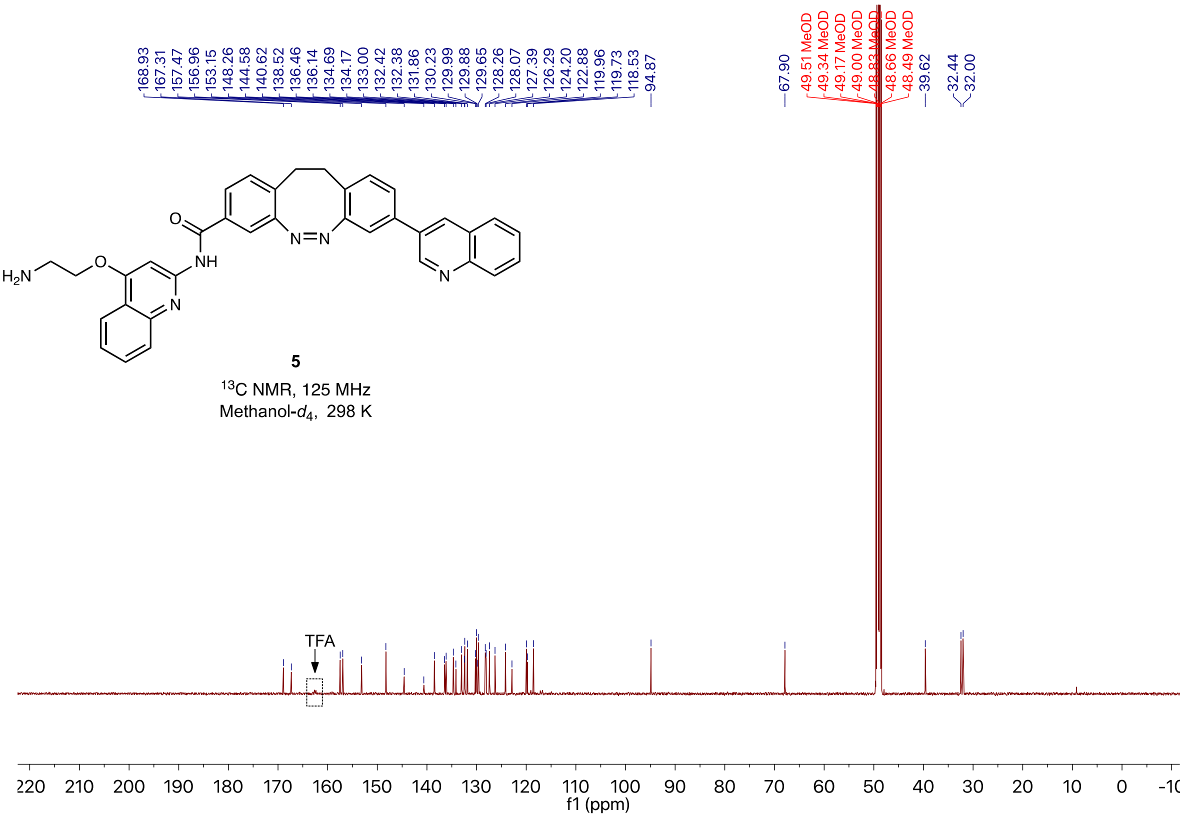

5

^{13}C NMR, 125 MHz

Methanol-d_4, 298 K

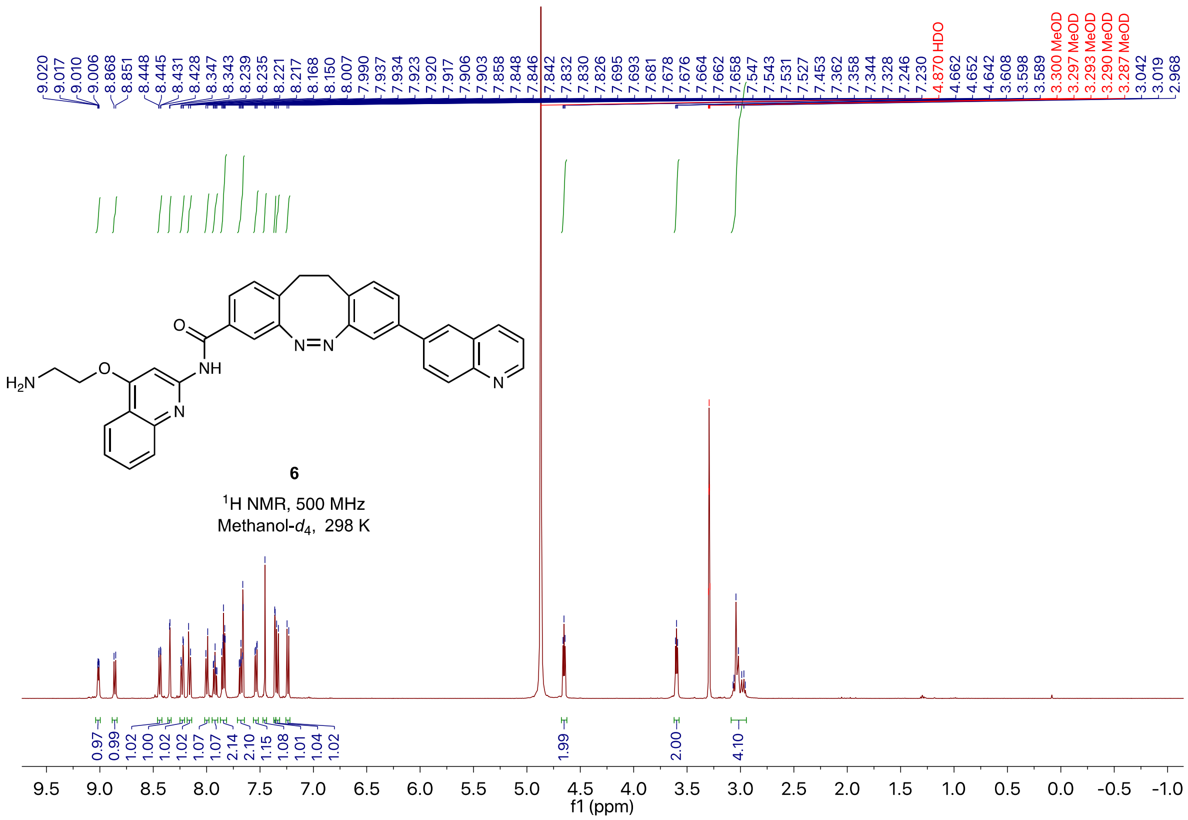

6
1H NMR, 500 MHz
Methanol-d4, 298 K
f1 (ppm)

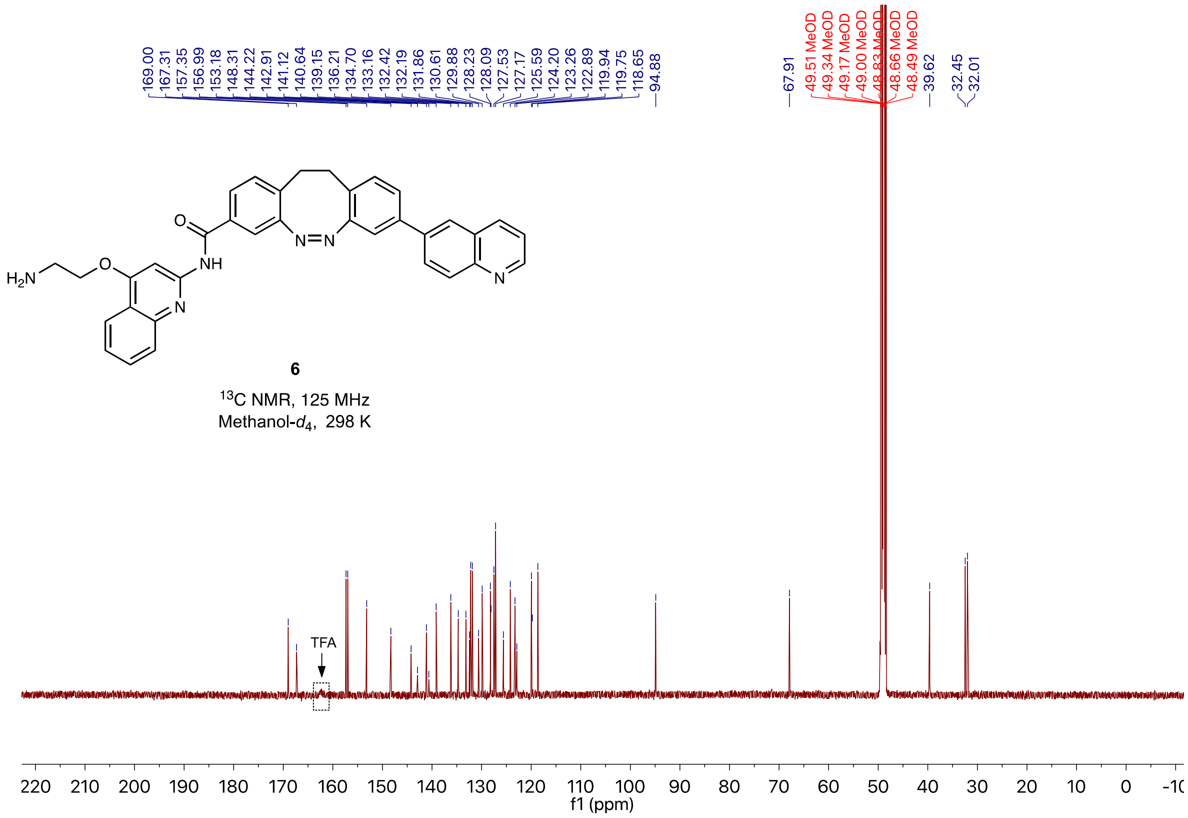

6
13C NMR, 125 MHz
Methanol-d4, 298 K
TFA
f1 (ppm)

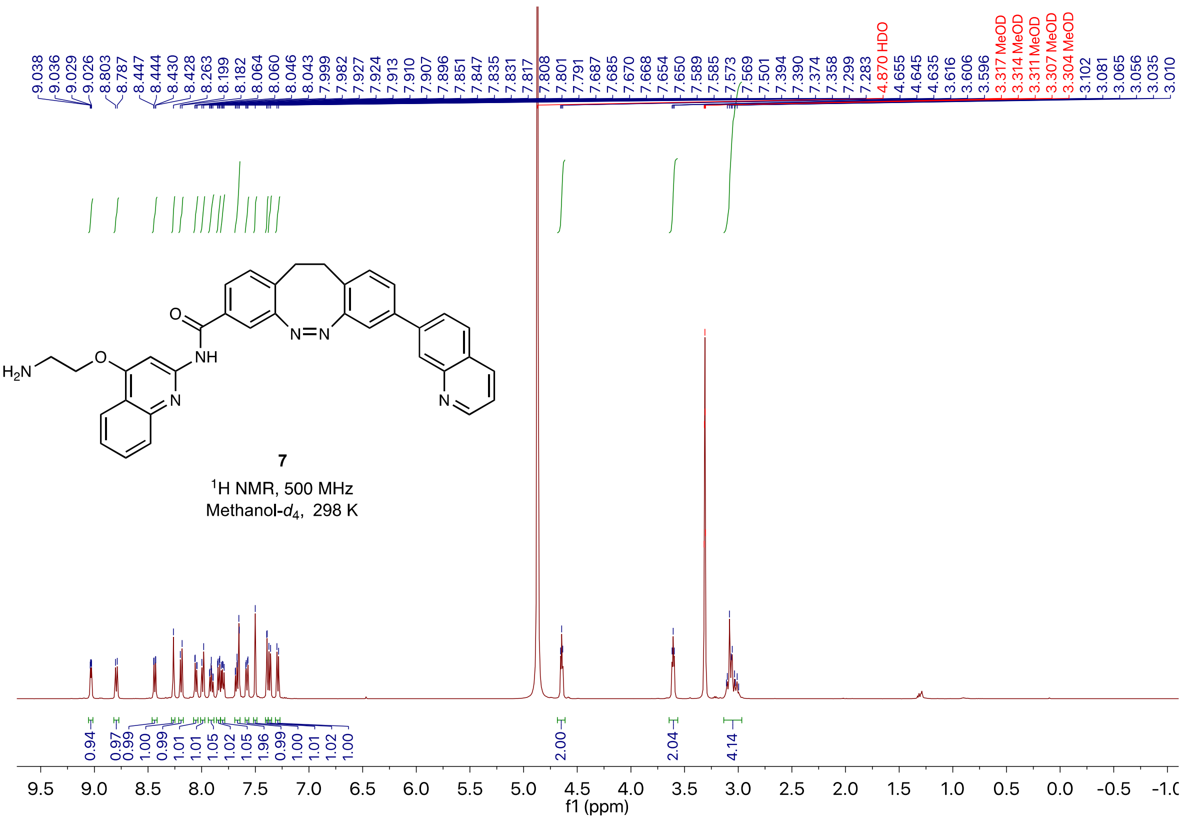
9.038
9.036
9.029
9.026
8.803
8.787
8.447
8.444
8.430
8.428
8.263
8.199
8.182
8.064
8.060
8.046
8.043
7.999
7.982
7.927
7.924
7.913
7.910
7.907
7.896
7.851
7.847
7.835
7.831
7.817
7.808
7.801
7.791
7.687
7.685
7.670
7.668
7.654
7.650
7.589
7.585
7.573
7.569
7.501
7.394
7.390
7.374
7.358
7.299
7.283
4.870 HDO
4.655
4.645
4.635
3.616
3.606
3.596
3.317 MeOD
3.314 MeOD
3.311 MeOD
3.307 MeOD
3.304 MeOD
3.102
3.081
3.065
3.056
3.035
3.010
O
NH
H2N
O
N
N=N
N
7
1H NMR, 500 MHz
Methanol-d4, 298 K
0.94
0.97
0.99
1.00
0.99
1.01
1.01
1.05
1.02
1.05
1.96
0.99
1.00
1.01
1.02
1.00
2.00
2.04
4.14
9.5
9.0
8.5
8.0
7.5
7.0
6.5
6.0
5.5
5.0
4.5
4.0
3.5
3.0
2.5
2.0
1.5
1.0
0.5
0.0
-0.5
f1 (ppm)

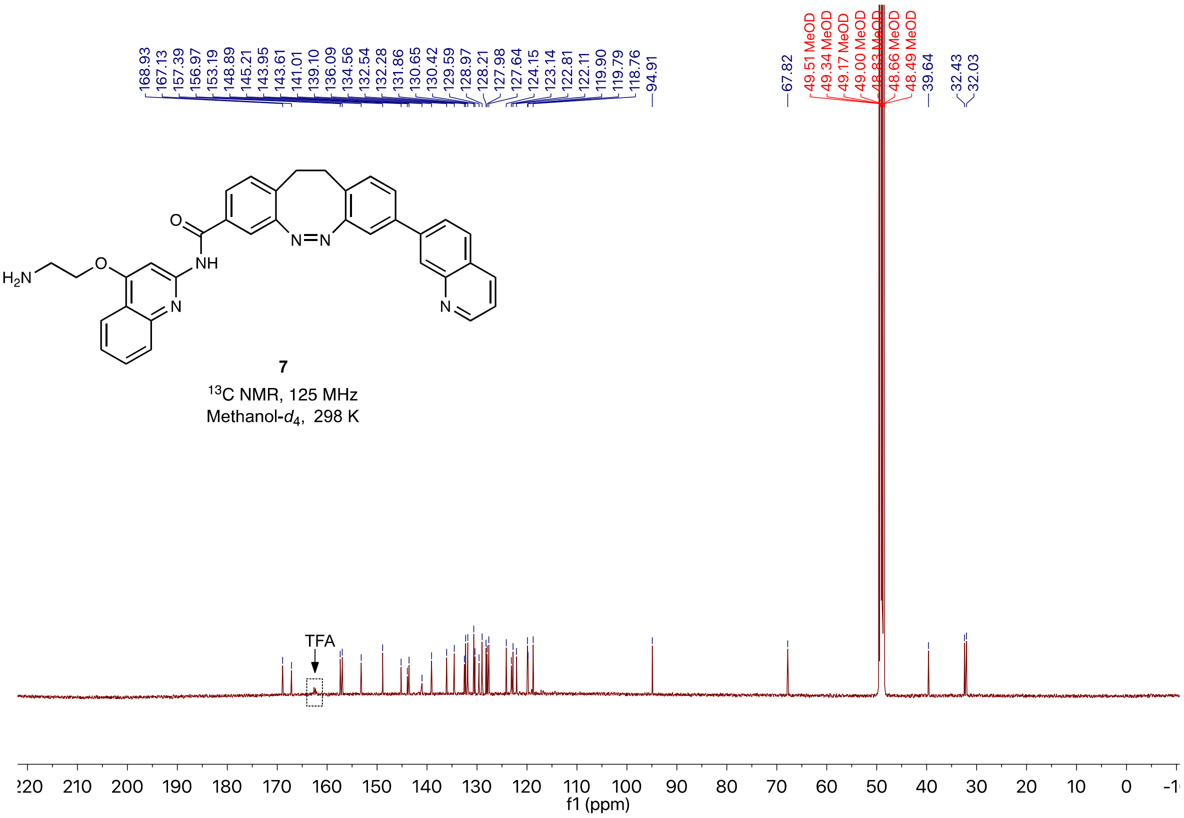
168.93
167.13
157.39
156.97
153.19
148.89
145.21
143.95
143.61
141.01
139.10
136.09
134.56
132.54
132.28
131.86
130.65
130.42
129.59
128.97
128.21
127.98
127.64
124.15
123.14
122.81
122.11
119.90
119.79
118.76
94.91
67.82
49.51 MeOD
49.34 MeOD
49.17 MeOD
49.00 MeOD
48.83 MeOD
48.66 MeOD
48.49 MeOD
39.64
32.43
32.03
O
NH
H2N
O
N
N=N
N
7
13C NMR, 125 MHz
Methanol-d4, 298 K
TFA
210
200
190
180
170
160
150
140
130
120
110
100
90
80
70
60
50
40
30
20
10
0
f1 (ppm)

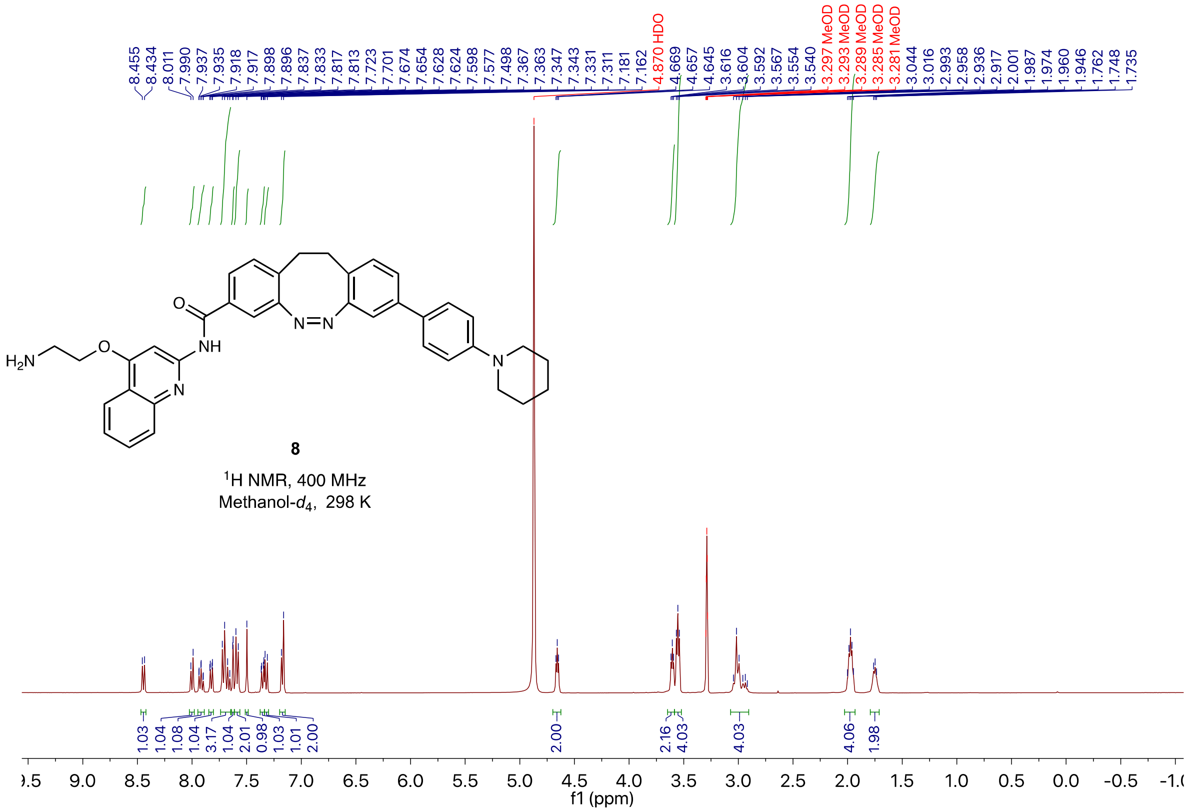
8.455
8.434
8.011
7.990
7.937
7.935
7.918
7.917
7.898
7.896
7.837
7.833
7.817
7.813
7.723
7.701
7.674
7.654
7.628
7.624
7.598
7.577
7.498
7.367
7.363
7.347
7.343
7.331
7.311
7.181
7.162
4.870 HDO
4.669
4.657
4.645
3.616
3.604
3.592
3.567
3.554
3.540
3.297 MeOD
3.293 MeOD
3.289 MeOD
3.285 MeOD
3.281 MeOD
3.044
3.016
2.993
2.958
2.936
2.917
2.001
1.987
1.974
1.960
1.946
1.762
1.748
1.735
O
NH
N=N
N
H2N
O
N
8
1H NMR, 400 MHz
Methanol-d4, 298 K
1.03
1.04
1.08
1.04
3.17
1.04
2.01
0.98
1.03
1.01
2.00
2.00
2.16
4.03
4.03
4.06
1.98
9.0
8.5
8.0
7.5
7.0
6.5
6.0
5.5
5.0
4.5
4.0
3.5
3.0
2.5
2.0
1.5
1.0
0.5
0.0
-0.5
f1 (ppm)

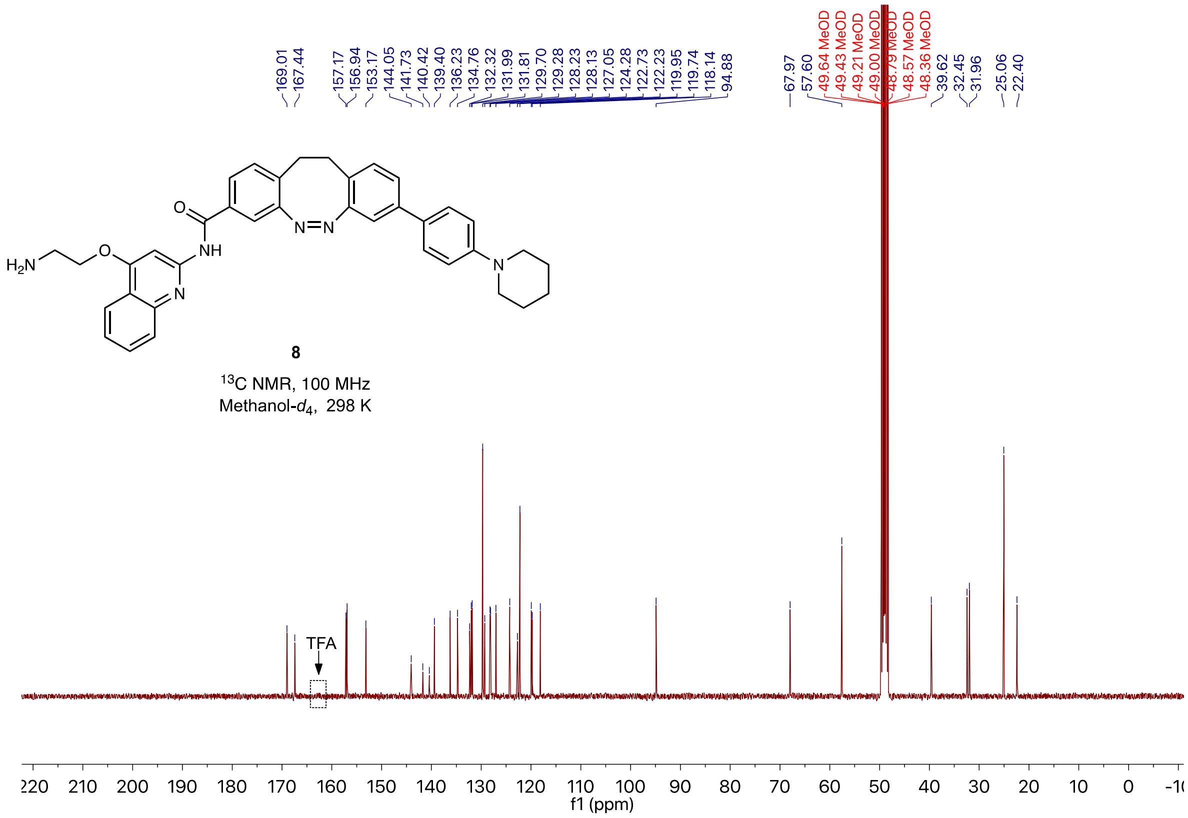
169.01
167.44
157.17
156.94
153.17
144.05
141.73
140.42
139.40
136.23
134.76
132.32
131.99
131.81
129.70
129.28
128.23
128.13
127.05
124.28
122.73
122.23
119.95
119.74
118.14
94.88
67.97
57.60
49.64 MeOD
49.43 MeOD
49.21 MeOD
49.00 MeOD
48.79 MeOD
48.57 MeOD
48.36 MeOD
39.62
32.45
31.96
25.06
22.40
O
NH
N=N
N
H2N
O
N
8
13C NMR, 100 MHz
Methanol-d4, 298 K
TFA
210
200
190
180
170
160
150
140
130
120
110
100
90
80
70
60
50
40
30
20
10
0
f1 (ppm)

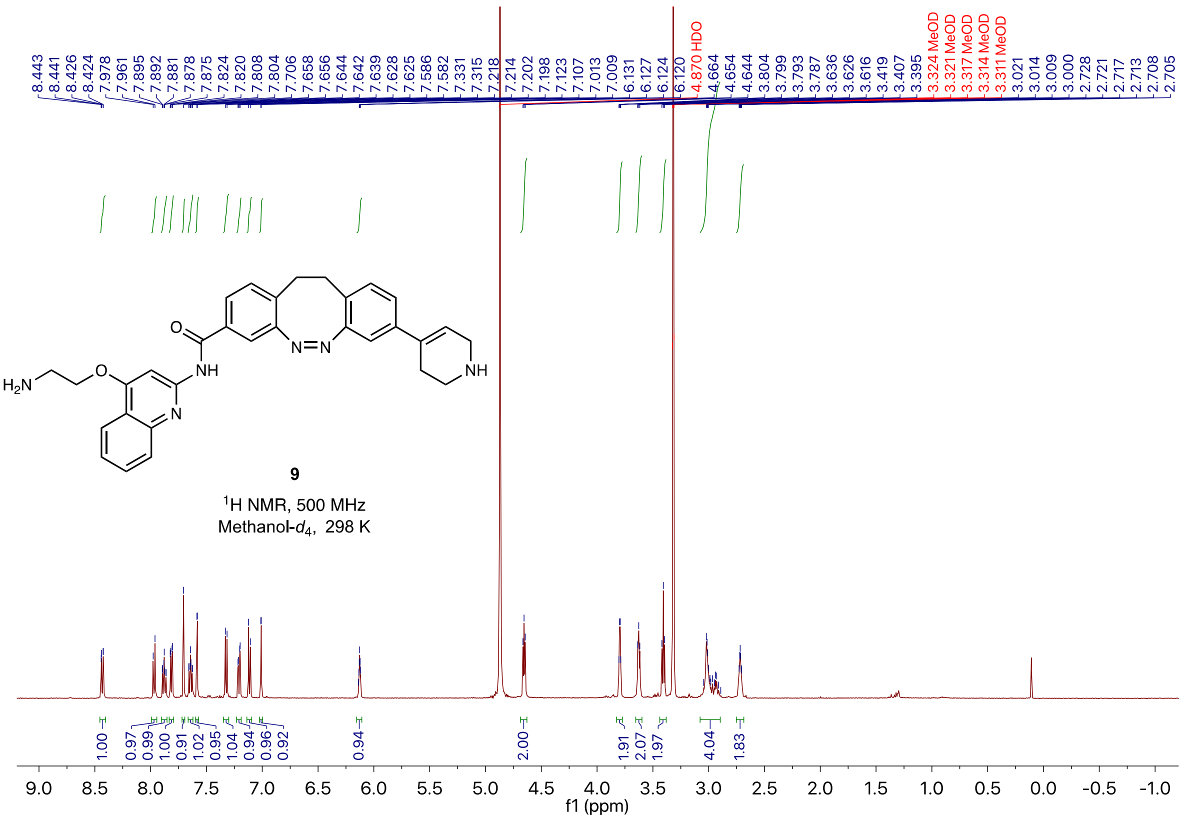
9
¹H NMR, 500 MHz
Methanol-d4, 298 K
f1 (ppm)

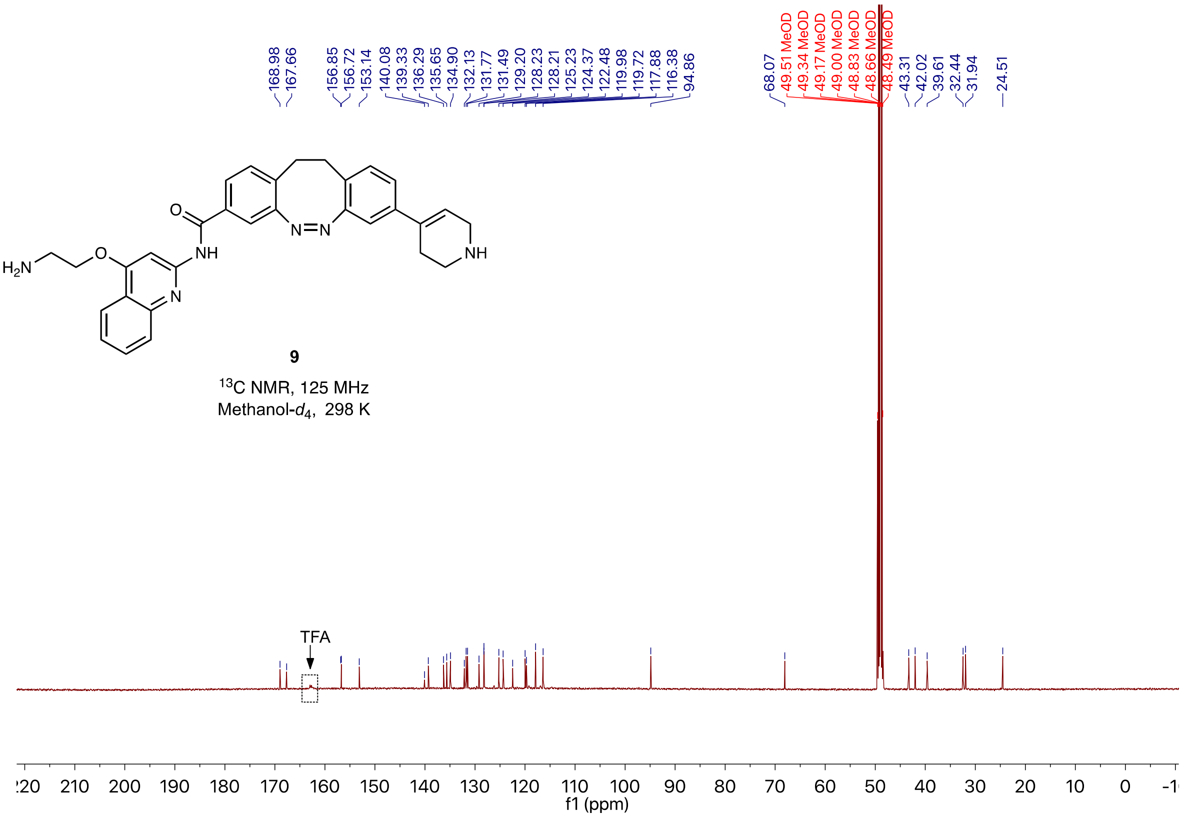
9
¹³C NMR, 125 MHz
Methanol-d4, 298 K
TFA
f1 (ppm)

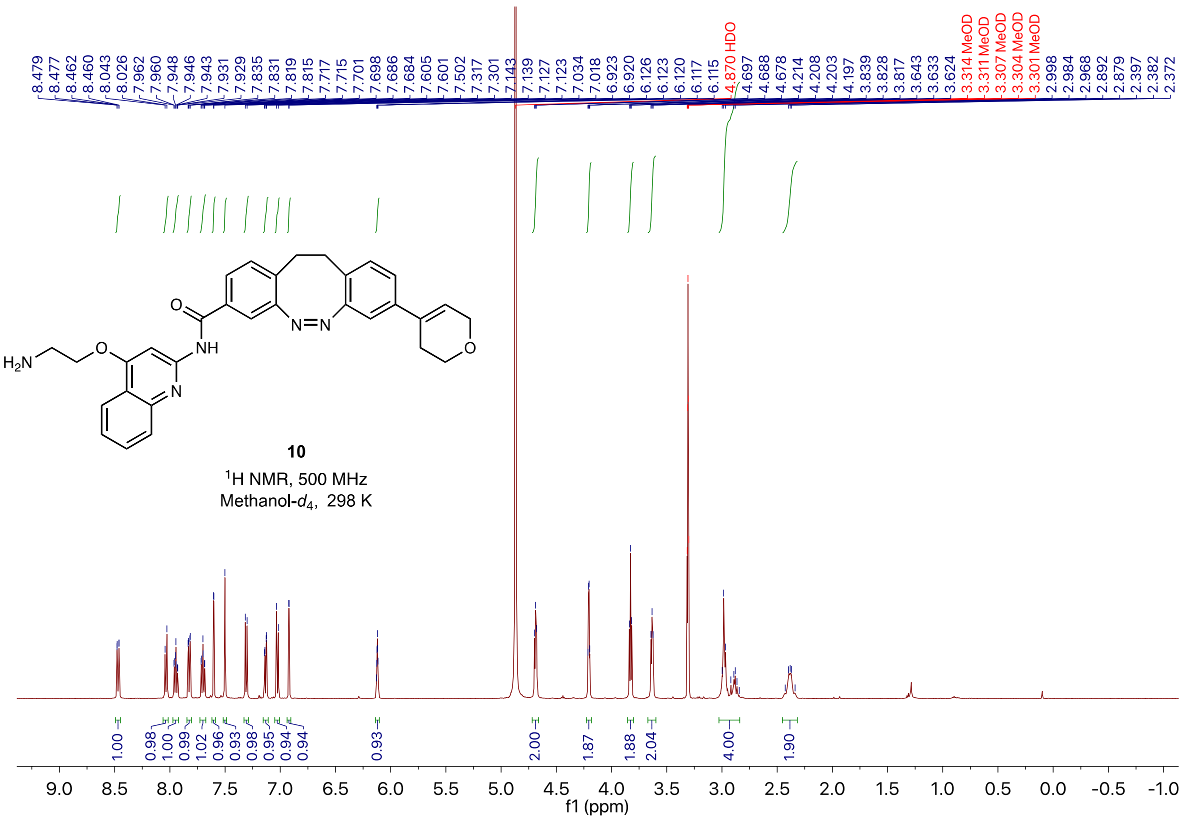
8.479 8.477 8.462 8.460 8.043 8.026 7.962 7.960 7.948 7.946 7.943 7.931 7.929 7.835 7.831 7.819 7.815 7.717 7.715 7.701 7.698 7.686 7.684 7.605 7.601 7.502 7.317 7.301 7.143 7.139 7.127 7.123 7.034 7.018 6.923 6.920 6.126 6.123 6.120 6.117 6.115
4.870 HDO
4.697 4.688 4.678 4.214 4.208 4.203 4.197 3.839 3.828 3.817 3.643 3.633 3.624
3.314 MeOD 3.311 MeOD 3.307 MeOD 3.304 MeOD 3.301 MeOD
2.998 2.984 2.968 2.892 2.879 2.397 2.382 2.372
O
N=N
NH
H_2N
O
N
O
10
^{1}H NMR, 500 MHz
Methanol-d_4, 298 K
1.00 0.98 1.00 0.99 1.02 0.96 0.93 0.98 0.95 0.94 0.94 0.93 2.00 1.87 1.88 2.04 4.00 1.90
9.0 8.5 8.0 7.5 7.0 6.5 6.0 5.5 5.0 4.5 4.0 3.5 3.0 2.5 2.0 1.5 1.0 0.5 0.0 -0.5 -1.0
f1 (ppm)

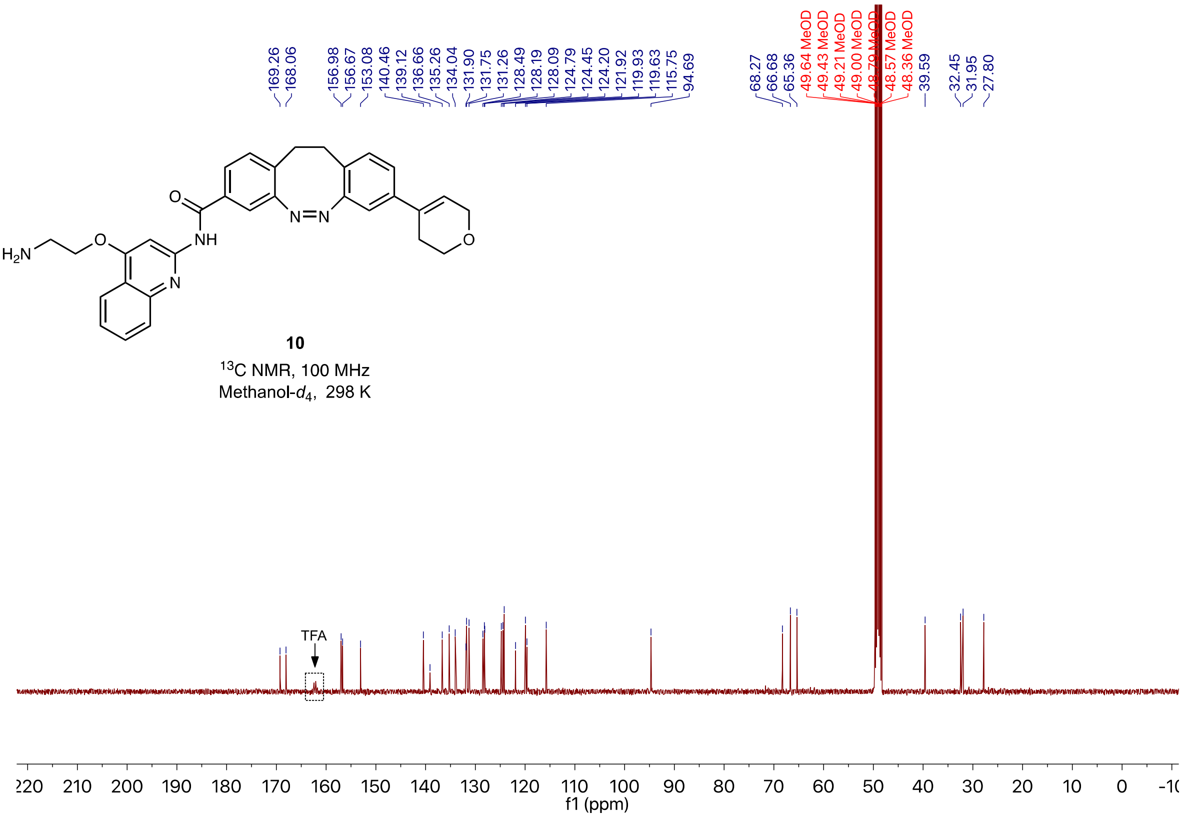
169.26 168.06 156.98 156.67 153.08 140.46 139.12 136.66 135.26 134.04 131.90 131.75 131.26 128.49 128.19 128.09 124.79 124.45 124.20 121.92 119.93 119.63 115.75 94.69
68.27 66.68 65.36
49.64 MeOD 49.43 MeOD 49.21 MeOD 49.00 MeOD 48.79 MeOD 48.57 MeOD 48.36 MeOD
39.59 32.45 31.95 27.80
O
N=N
NH
H_2N
O
N
O
10
^{13}C NMR, 100 MHz
Methanol-d_4, 298 K
TFA
220 210 200 190 180 170 160 150 140 130 120 110 100 90 80 70 60 50 40 30 20 10 0 -10
f1 (ppm)

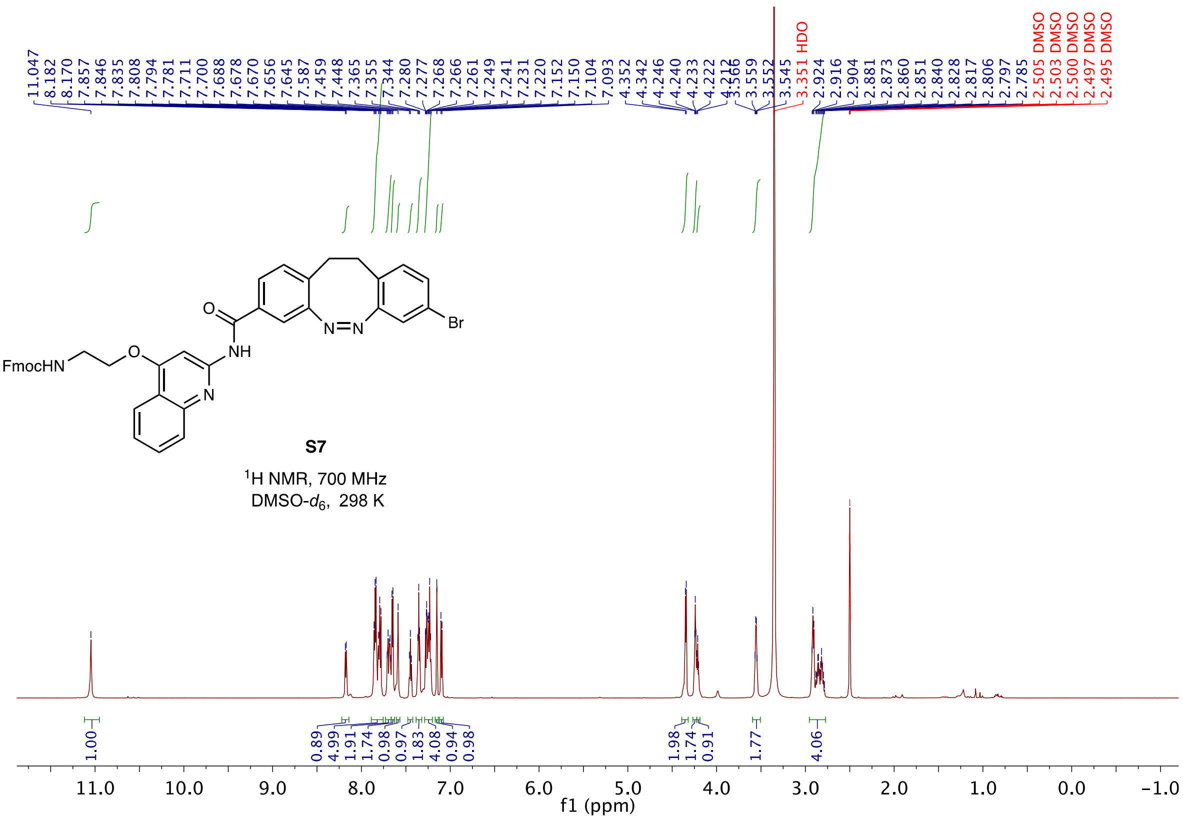

S7

^{1}H NMR, 700 MHz
DMSO-d_6, 298 K

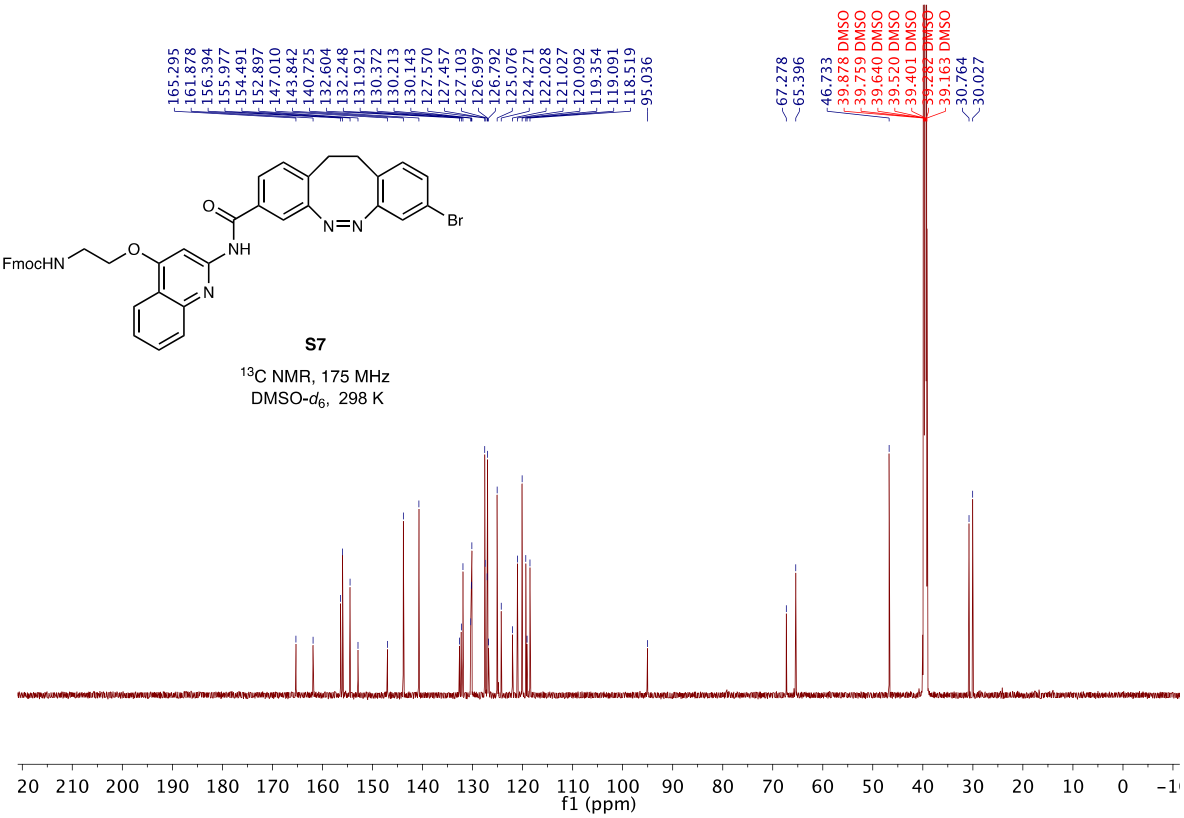

S7

^{13}C NMR, 175 MHz
DMSO-d_6, 298 K

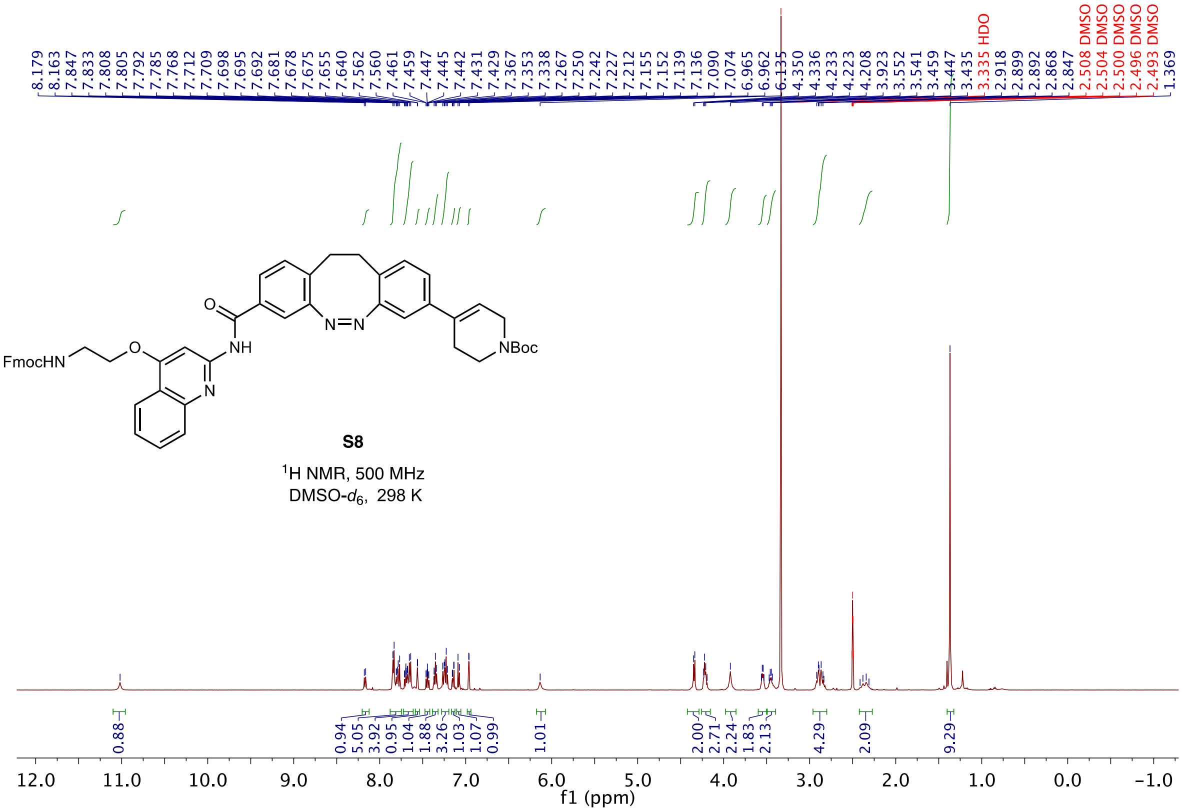
8.179 8.163 7.847 7.833 7.808 7.805 7.792 7.785 7.768 7.712 7.709 7.698 7.695 7.692 7.681 7.678 7.675 7.655 7.640 7.562 7.560 7.461 7.459 7.447 7.445 7.442 7.431 7.429 7.367 7.353 7.338 7.267 7.250 7.242 7.227 7.212 7.155 7.152 7.139 7.136 7.090 7.074 6.965 6.962 6.135 4.350 4.336 4.233 4.223 4.208 3.923 3.552 3.541 3.459 3.447 3.435
3.335 HDO
2.918 2.899 2.892 2.868 2.847
2.508 DMSO 2.504 DMSO 2.500 DMSO 2.496 DMSO 2.493 DMSO
1.369
O
NH
N=N
NBoc
FmocHN
O
N
S8
^{1}H NMR, 500 MHz
DMSO-d_6, 298 K
0.88 0.94 5.05 3.92 0.95 1.04 1.88 1.03 3.26 1.07 0.99 1.01 2.00 2.71 2.24 1.83 2.13 4.29 2.09 9.29
12.0 11.0 10.0 9.0 8.0 7.0 6.0 5.0 4.0 3.0 2.0 1.0 0.0 −1.0
f1 (ppm)

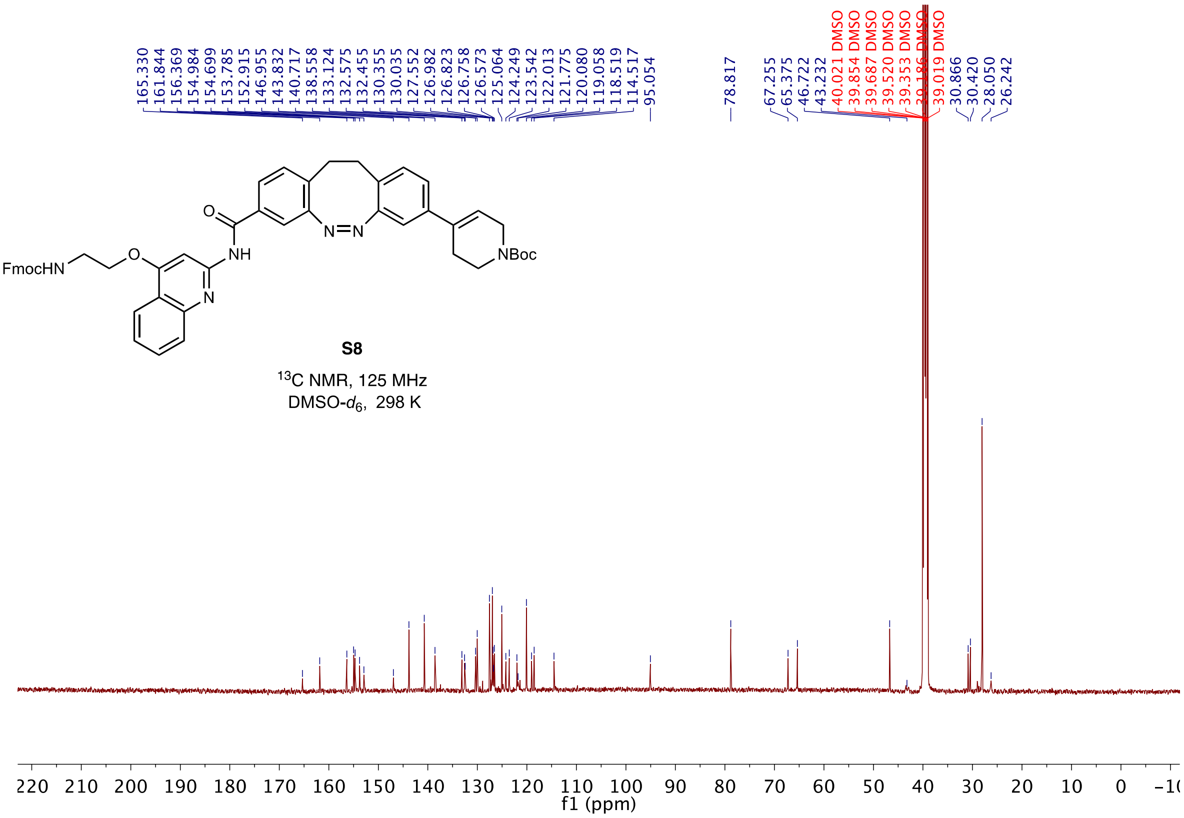
165.330 161.844 156.369 154.984 154.699 153.785 152.915 146.955 143.832 140.717 138.558 133.124 132.575 132.455 130.355 130.035 127.552 126.982 126.823 126.758 126.573 125.064 124.249 123.542 122.013 121.775 120.080 119.058 118.519 114.517 95.054
78.817
67.255 65.375 46.722 43.232
40.021 DMSO 39.854 DMSO 39.687 DMSO 39.520 DMSO 39.353 DMSO 39.186 DMSO 39.019 DMSO
30.866 30.420 28.050 26.242
O
NH
N=N
NBoc
FmocHN
O
N
S8
^{13}C NMR, 125 MHz
DMSO-d_6, 298 K
220 210 200 190 180 170 160 150 140 130 120 110 100 90 80 70 60 50 40 30 20 10 0 −10
f1 (ppm)

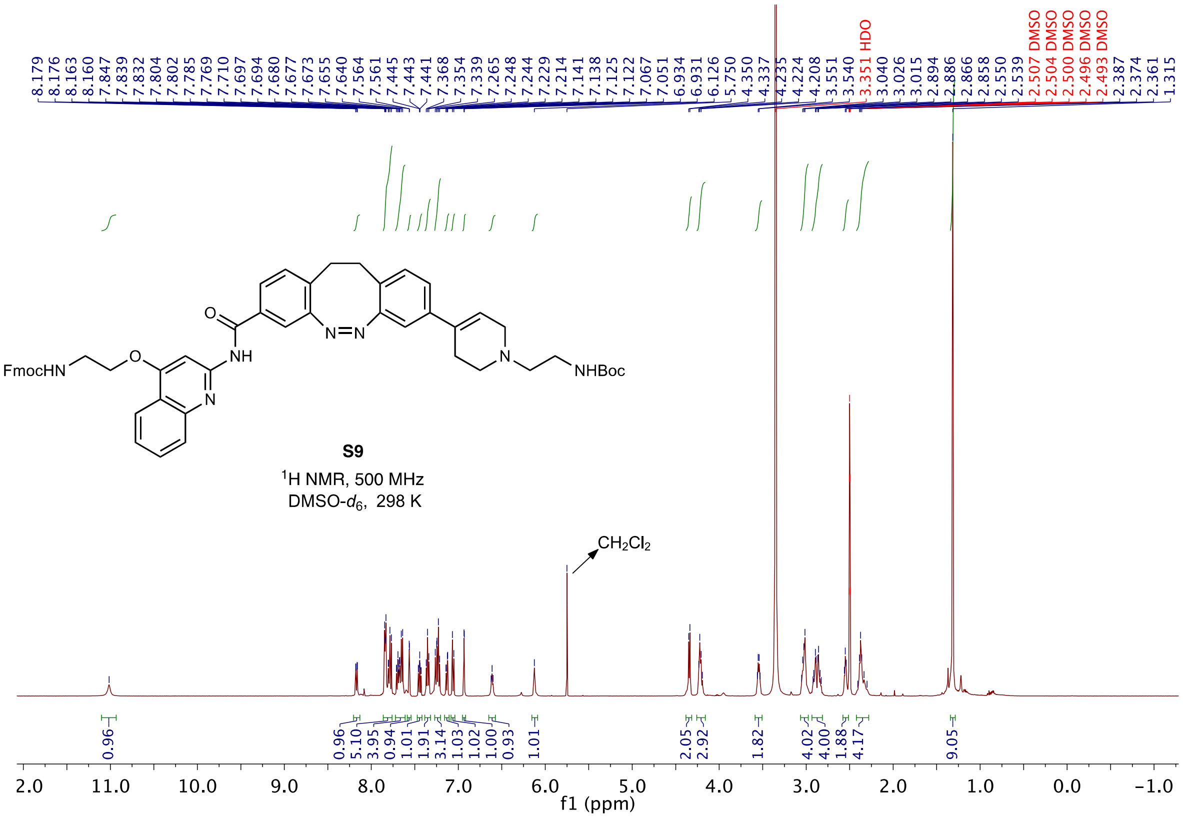
8.179 8.176 8.163 8.160 7.847 7.839 7.832 7.804 7.802 7.785 7.769 7.710 7.697 7.694 7.680 7.677 7.673 7.655 7.640 7.564 7.561 7.445 7.443 7.441 7.368 7.354 7.339 7.265 7.248 7.244 7.229 7.214 7.141 7.138 7.125 7.122 7.067 7.051 6.934 6.931 6.126 5.750 4.350 4.337 4.235 4.224 4.208 3.551 3.540
3.351 HDO
3.040 3.026 3.015 2.894 2.886 2.866 2.858 2.550 2.539
2.507 DMSO 2.504 DMSO 2.500 DMSO 2.496 DMSO 2.493 DMSO
2.387 2.374 2.361 1.315
FmocHN
O
NH
N
N=N
N
NHBoc
S9
1H NMR, 500 MHz
DMSO-d6, 298 K
CH2Cl2
0.96 0.96 5.10 3.95 0.94 1.01 1.91 1.03 3.14 1.02 1.00 0.93 1.01 2.05 2.92 1.82 4.02 4.00 1.88 4.17 9.05
2.0 11.0 10.0 9.0 8.0 7.0 6.0 5.0 4.0 3.0 2.0 1.0 0.0 -1.0
f1 (ppm)

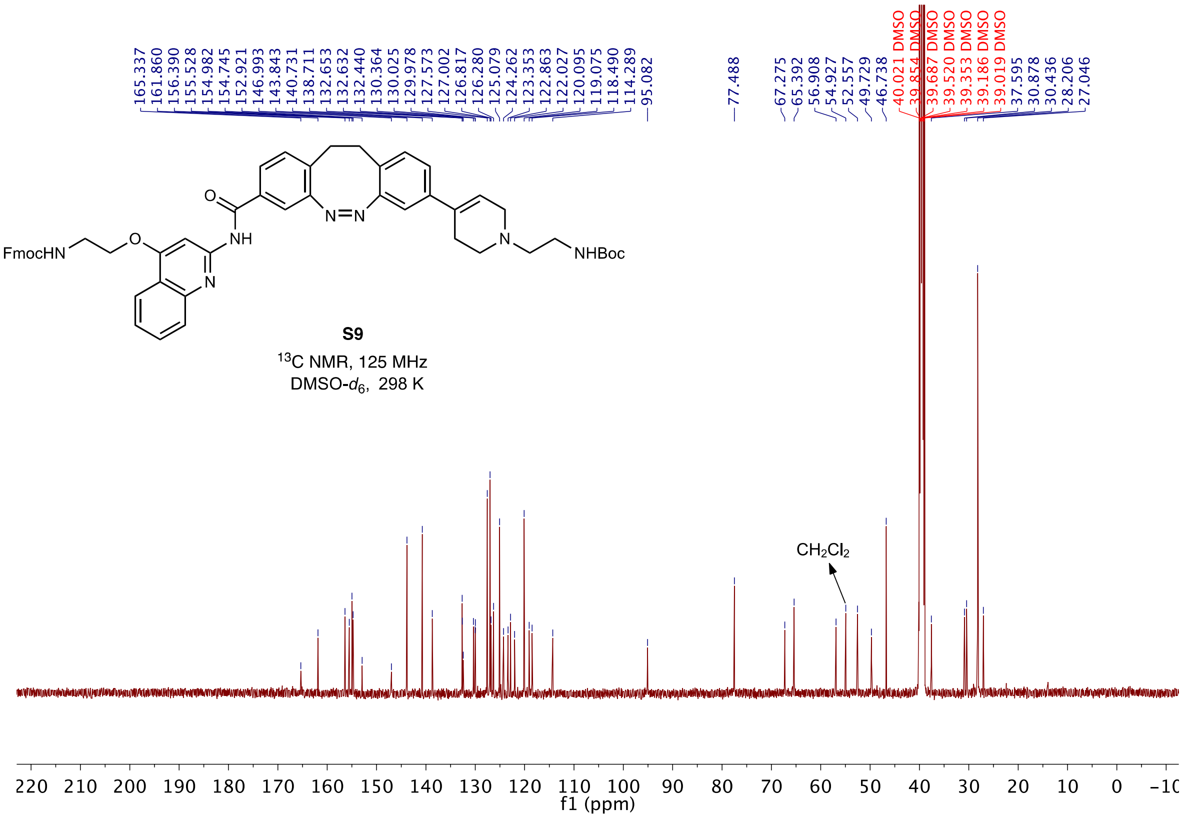
165.337 161.860 156.390 155.528 154.982 154.745 152.921 146.993 143.843 140.731 138.711 132.653 132.632 132.440 130.364 130.025 129.978 127.573 127.002 126.817 126.280 125.079 124.262 123.353 122.863 122.027 120.095 119.075 118.490 114.289 95.082
77.488
67.275 65.392 56.908 54.927 52.557 49.729 46.738
40.021 DMSO 39.854 DMSO 39.687 DMSO 39.520 DMSO 39.353 DMSO 39.186 DMSO 39.019 DMSO
37.595 30.878 30.436 28.206 27.046
FmocHN
O
NH
N
N=N
N
NHBoc
S9
13C NMR, 125 MHz
DMSO-d6, 298 K
CH2Cl2
220 210 200 190 180 170 160 150 140 130 120 110 100 90 80 70 60 50 40 30 20 10 0 -10
f1 (ppm)

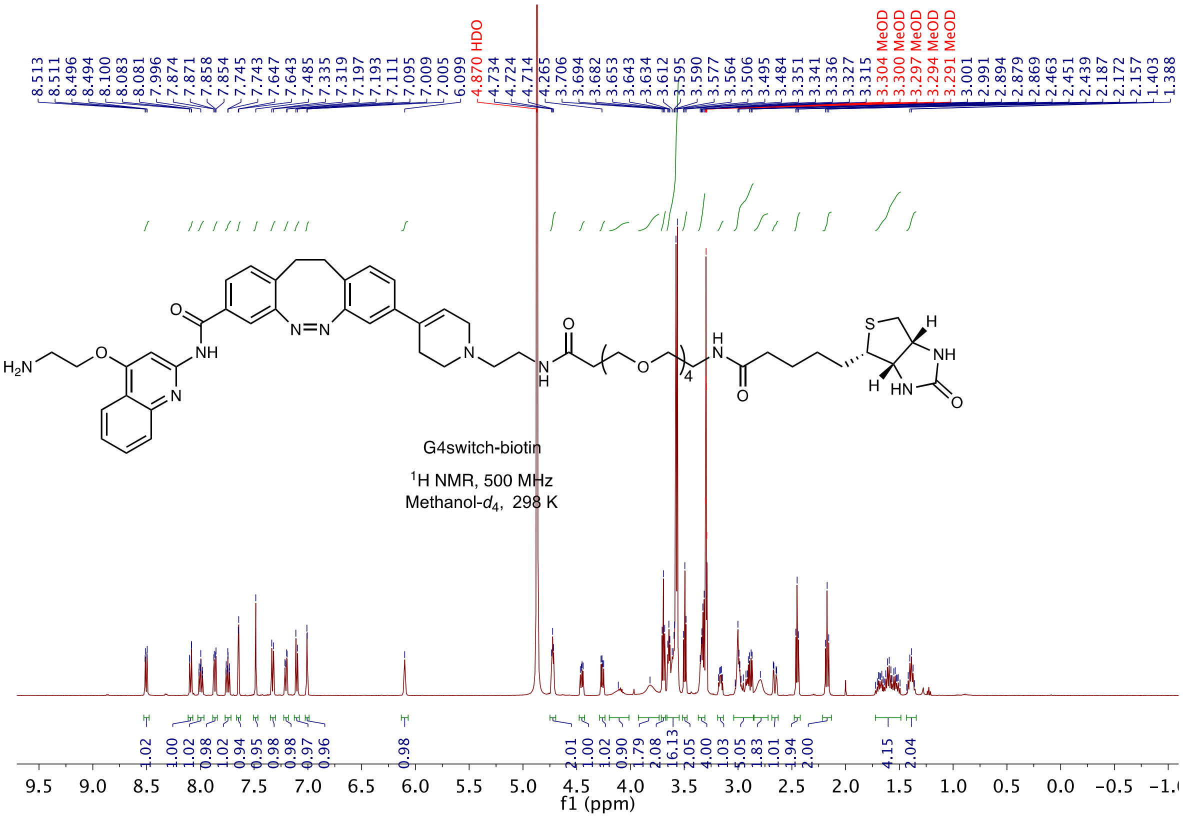
8.513 8.511 8.496 8.100 8.083 8.081 7.996 7.874 7.871 7.858 7.854 7.745 7.743 7.647 7.643 7.485 7.335 7.319 7.197 7.193 7.111 7.095 7.009 7.005 6.099
4.870 HDO
4.734 4.724 4.714 4.265 3.706 3.694 3.682 3.653 3.643 3.634 3.612 3.595 3.590 3.577 3.564 3.506 3.495 3.484 3.351 3.341 3.336 3.327 3.315
3.304 MeOD 3.300 MeOD 3.297 MeOD 3.294 MeOD 3.291 MeOD
3.001 2.991 2.894 2.879 2.869 2.463 2.451 2.439 2.187 2.172 2.157 1.403 1.388
G4switch-biotin
^{1}H NMR, 500 MHz
Methanol-d_4, 298 K
1.02 1.00 1.02 0.98 1.02 0.94 0.95 0.98 0.98 0.97 0.96 0.98 2.01 1.00 1.02 0.90 1.79 2.08 16.13 2.05 4.00 1.03 5.05 1.83 1.01 1.94 2.00 4.15 2.04
f1 (ppm)

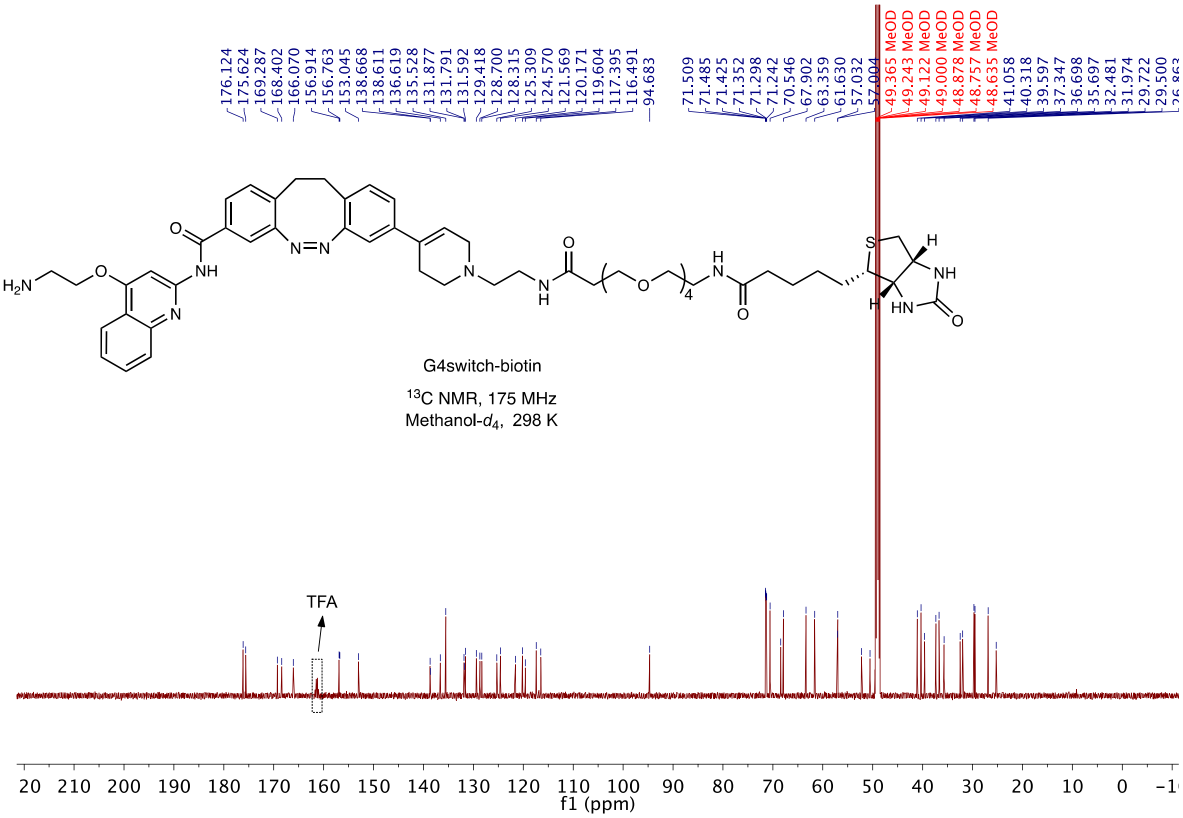
176.124 175.624 169.287 168.402 166.070 156.914 156.763 153.045 138.668 138.611 136.619 135.528 131.877 131.791 131.592 129.418 128.700 128.315 125.309 124.570 121.569 120.171 119.604 117.395 116.491 94.683
71.509 71.485 71.425 71.352 71.298 71.242 70.546 67.902 63.359 61.630 57.032 57.004
49.365 MeOD 49.243 MeOD 49.122 MeOD 49.000 MeOD 48.878 MeOD 48.757 MeOD 48.635 MeOD
41.058 40.318 39.597 37.347 36.698 35.697 32.481 31.974 29.722 29.500
G4switch-biotin
^{13}C NMR, 175 MHz
Methanol-d_4, 298 K
TFA
f1 (ppm)

Characterization of the emission spectra of 405 nm and 525 nm LED lights

Sahlmann Photochemical Solutions

LED module 3x 405 nm

LED type: NVSU233A-U405
rank: k.A.
typ. optical power: 3x 900 mW

emission characteristics:
spectrometer: Ocean Optics USB 4000
peak wavelength: 407 nm
FWHM: 13 nm

spectral characteristics:

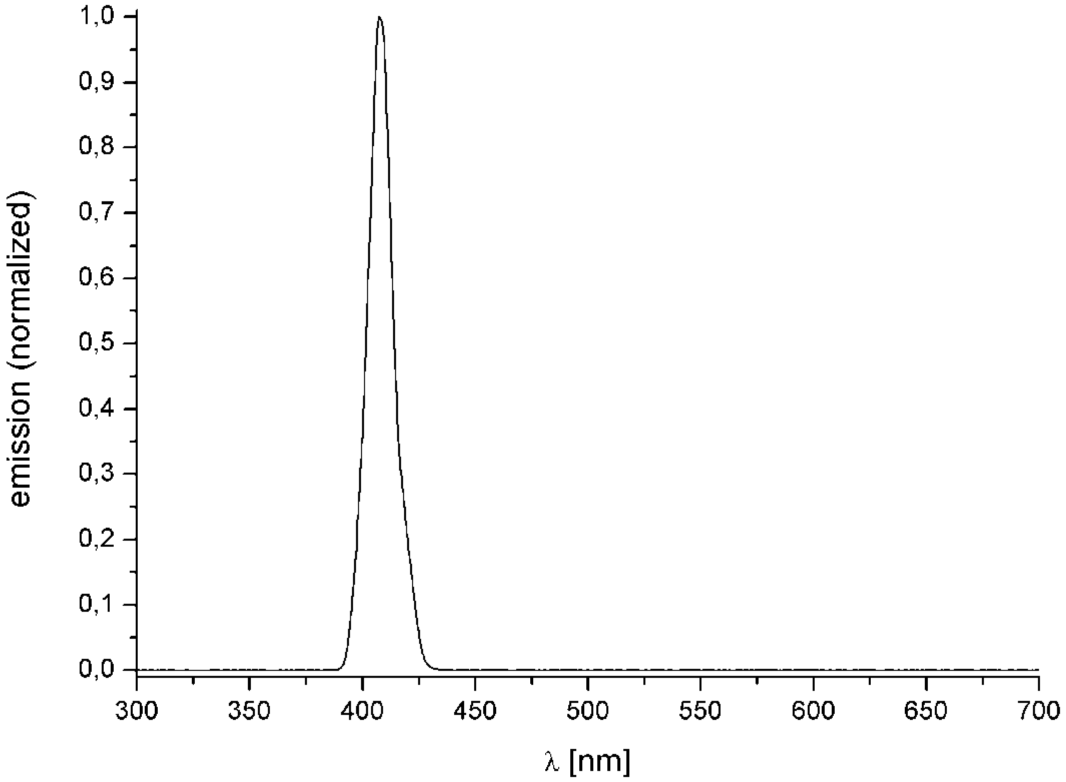

Sahlmann Photochemical Solutions

LED module 3x 525 nm

LED type: 3x Nichia NCSG219B-V1
rank: k.A.
typ. optical power: 3x 600 mW

emission characteristics:

spectrometer: Ocean Optics USB 4000
peak wavelength: 525nm
FWHM: 32 nm

spectral characteristics:

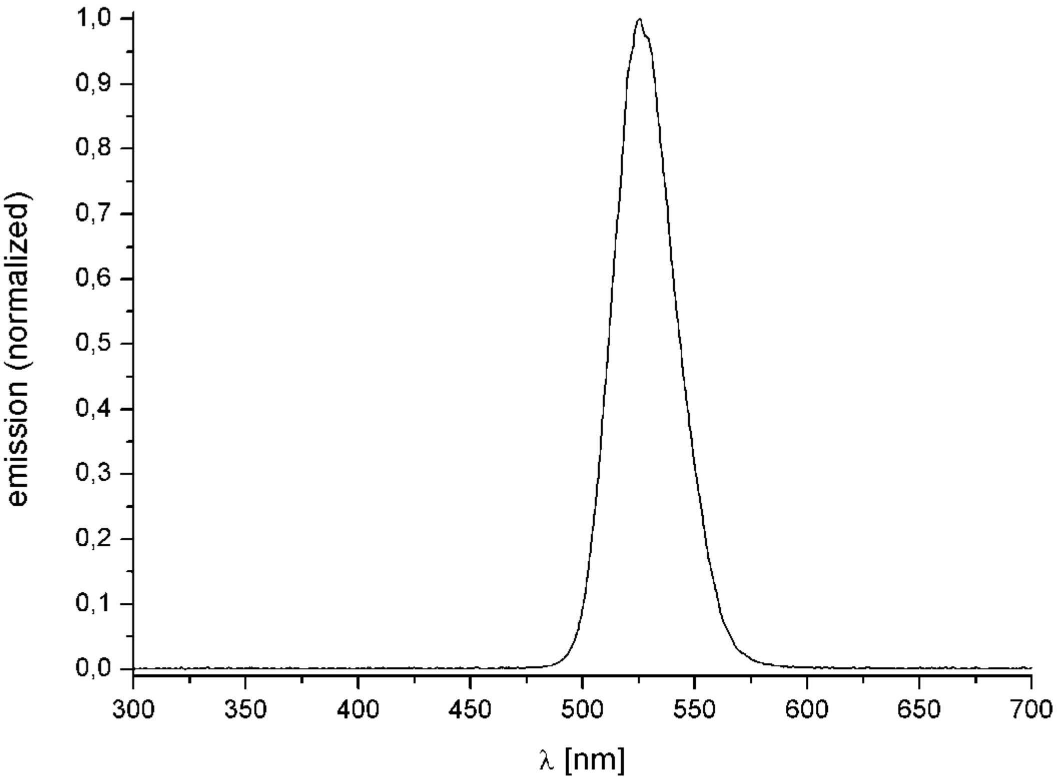

References

1. Cabré, G. *et al.* Synthetic photoswitchable neurotransmitters based on bridged azobenzenes. *Org. Lett.* **21**, 3780–3784 (2019).
2. Rodriguez, R. *et al.* A novel small molecule that alters shelterin integrity and triggers a DNA-damage response at telomeres. *J. Am. Chem. Soc.* **130**, 15758–15759 (2008).
3. Schindelin, J. *et al.* Fiji: an open-source platform for biological-image analysis. *Nat. Methods* **9**, 676–682 (2012).
4. Maier, M. S. *et al.* Oxidative approach enables efficient access to cyclic azobenzenes. *J. Am. Chem. Soc.* **141**, 17295–17304 (2019).

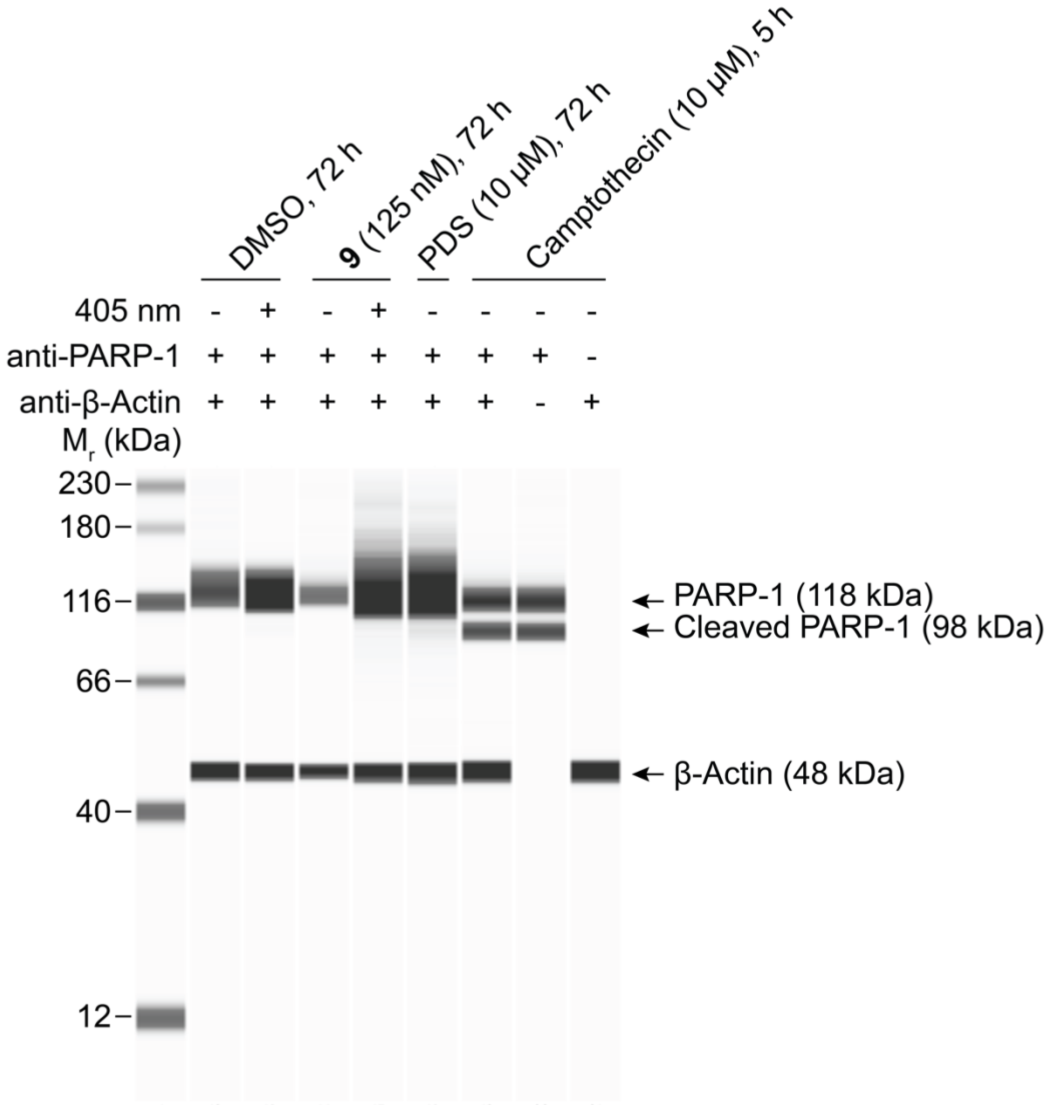

Source Data for Supplementary Fig. 8 | Unprocessed Western blot image of PARP-1.
