# Supplementary material for: Optical control of gene expression using a DNA G-quadruplex targeting reversible photoswitch
Source: Nat Chem. 2025 Apr 3;17(6):875–82. doi: 10.1038/s41557-025-01792-1 (PMC12141046; doi:10.1038/s41557-025-01792-1)
Supplement: Supplementary file 2 — Reporting Summary [file 41557_2025_1792_MOESM2_ESM.pdf]

nature portfolio

Corresponding author(s): Shankar Balasubramanian

Last updated by author(s): Jan 31, 2025

Reporting Summary

Nature Portfolio wishes to improve the reproducibility of the work that we publish. This form provides structure for consistency and transparency in reporting. For further information on Nature Portfolio policies, see our Editorial Policies and the Editorial Policy Checklist.

Statistics

For all statistical analyses, confirm that the following items are present in the figure legend, table legend, main text, or Methods section.

n/a	Confirmed	
☐	☒	The exact sample size (*n*) for each experimental group/condition, given as a discrete number and unit of measurement
☐	☒	A statement on whether measurements were taken from distinct samples or whether the same sample was measured repeatedly
☐	☒	The statistical test(s) used AND whether they are one- or two-sided<br>*Only common tests should be described solely by name; describe more complex techniques in the Methods section.*
☒	☐	A description of all covariates tested
☒	☐	A description of any assumptions or corrections, such as tests of normality and adjustment for multiple comparisons
☐	☒	A full description of the statistical parameters including central tendency (e.g. means) or other basic estimates (e.g. regression coefficient) AND variation (e.g. standard deviation) or associated estimates of uncertainty (e.g. confidence intervals)
☐	☒	For null hypothesis testing, the test statistic (e.g. *F*, *t*, *r*) with confidence intervals, effect sizes, degrees of freedom and *P* value noted<br>*Give P values as exact values whenever suitable.*
☒	☐	For Bayesian analysis, information on the choice of priors and Markov chain Monte Carlo settings
☒	☐	For hierarchical and complex designs, identification of the appropriate level for tests and full reporting of outcomes
☒	☐	Estimates of effect sizes (e.g. Cohen's *d*, Pearson's *r*), indicating how they were calculated

Our web collection on statistics for biologists contains articles on many of the points above.

Software and code

Policy information about availability of computer code

Data collection	NMR data were collected on a Bruker 400 MHz Avance III HD Spectrometer, a 500 MHz Avance III Smart Probe Spectrometer and a 700 MHz TXO Cryoprobe Spectrometer.<br>HRMS data were collected on a Waters LCT Premier (ESI) spectrometer.<br>UV-Vis and CD spectra were collected on a Agilent Cary 3500 UV-Vis spectrophotometer and a Applied Photophysics Chirascan Plus Spectrometer, respectively.<br>FID binding data were collected from a BMG PHERAstar Plus reader.<br>The cell staining images were collected on a Bio-Rad ChemiDoc MP system.<br>A Bio-Rad CFX96 Touch Real-Time PCR Detection System was used for concentration quantification of sequencing libraries.<br>Sequencing data were collected on a NextSeq 500 sequencer (Illumina) and a NextSeq 2000 sequencer (Illumina).
Data analysis	Physicochemical properties of compounds were calculated in ChemAxon MarvinSketch (version 21.4.0, http://www.chemaxon.com).<br>NMR data were processed in MestReNova (version 12.0.1).<br>UV-Vis, CD titration, and FID assay data were processed and analysed in GraphPad Prism 10 (version 10.2.2).<br>Cell staining images were processed in software Fiji (version 2.14.0/1.54f).<br>Bioinformatics data analyses and processing were performed using Bash, R (version 4.1) and Python (version >=3.4) programming languages.<br>The following tools were also used: demuxIllumina (version 3.0.9), FastQC (version 0.11.8), MultiQC (version 1.11), cutadapt (version 1.18), BWA (version 0.7.17-r1188), Picard (version 2.20.3), Seacr (version 1.3), EnhancedVolcano (v.1.16.0), deepTools (version 2.0), DiffBind (version 3.10.1), Gencode v37, SlamDunk package (v.0.3.4) and DESeq2 (version 1.2.10).<br>The analysis scripts are available on the GitHub page dedicated to this study: https://github.com/sblab-informatics/G4-switch

For manuscripts utilizing custom algorithms or software that are central to the research but not yet described in published literature, software must be made available to editors and reviewers. We strongly encourage code deposition in a community repository (e.g. GitHub). See the Nature Portfolio guidelines for submitting code & software for further information.

April 2023

Data

Policy information about availability of data

All manuscripts must include a data availability statement. This statement should provide the following information, where applicable:

- Accession codes, unique identifiers, or web links for publicly available datasets
- A description of any restrictions on data availability
- For clinical datasets or third party data, please ensure that the statement adheres to our policy

The sequencing data generated in this study are available at the Gene Expression Omnibus (GEO) repository under accession number GSE261837. The previously published GRCh38 (hg38) (https://www.ensembl.org/Homo_sapiens/Info/Index) and OQs (GSE110582) datasets were used.

Research involving human participants, their data, or biological material

Policy information about studies with human participants or human data. See also policy information about sex, gender (identity/presentation), and sexual orientation and race, ethnicity and racism.

Reporting on sex and gender	n/a
Reporting on race, ethnicity, or other socially relevant groupings	n/a
Population characteristics	n/a
Recruitment	n/a
Ethics oversight	n/a

Note that full information on the approval of the study protocol must also be provided in the manuscript.

Field-specific reporting

Please select the one below that is the best fit for your research. If you are not sure, read the appropriate sections before making your selection.

☒ Life sciences ☐ Behavioural & social sciences ☐ Ecological, evolutionary & environmental sciences

For a reference copy of the document with all sections, see nature.com/documents/nr-reporting-summary-flat.pdf

Life sciences study design

All studies must disclose on these points even when the disclosure is negative.

Sample size	According to a previous Chem-map study (Yu, Z. et al. Nat. Biotechnol. 41, 1265–1271 (2023)), 600,000 cells per sample were used for mapping G4 ligand binding sites; the same number was used in this study. For G4-CUT&Tag, a previous study (Hui, W. W. I. et al. Sci. Rep. 11, 1–7 (2021)) showed that the total number of G4 peaks mapped increased with cell input, ranging from 10,000 to 100,000 cells. To ensure comprehensive G4 peak capture and minimize underrepresentation, we increased the input to 300,000 cells per sample. For SLAM-seq, 210,000 cells per well were seeded in 6-well plates and grown to 70–80% confluence to ensure sufficient total RNA for iodoacetamide (IAA) alkylation (Lexogen, cat. no. 061) and preparation of 3′-end mRNA sequencing libraries (Lexogen, cat. no. 015) following the manufacturer's protocol (https://www.lexogen.com/). For crystal violet staining experiments, 35,000 cells per well in 6-well plates were seeded to ensure appropriate confluence for quantification. For Western blot experiments, 50,000 cells per well were seeded in 6-well plates to ensure adequate protein extraction for analysis using the Jess automated Western blot system (ProteinSimple) following the manufacturer's protocol (https://www.bio-techne.com/instruments/simple-western).
Data exclusions	No data were excluded from analyses in this study.
Replication	Chem-map experiments were performed in three independent biological replicates, each with three technical replicates, while Chem-map competition experiments were conducted in three independent biological replicates. G4-CUT&Tag was performed in five technical replicates. SLAM-seq was carried out in four biological replicates. Crystal violet staining experiments were conducted in three independent biological replicates (n = 3), with relative cell viability quantified and shown as mean ± standard deviation (s.d.). For Western blot experiments, similar results were obtained in three independent biological replicates. Cell viability assays were performed and are presented as mean ± s.d. from four replicates (n = 4). The FID assay results are shown as mean ± s.d., calculated from three or four technical replicates (n = 3 or 4), while CD spectra were recorded from three scans and presented as the average of the three scans. Similar results were consistently observed in at least two biological replicates for the FID assay and CD experiments.<br>All experiments were reliably reproducible.
Randomization	No randomization was performed, as control conditions were run in parallel to avoid systematic measurement bias.

Blinding	No blinding was performed, as control conditions were run in parallel to avoid systematic measurement bias.

Reporting for specific materials, systems and methods

We require information from authors about some types of materials, experimental systems and methods used in many studies. Here, indicate whether each material, system or method listed is relevant to your study. If you are not sure if a list item applies to your research, read the appropriate section before selecting a response.

Materials & experimental systems

n/a	Involved in the study
☐	☒ Antibodies
☐	☒ Eukaryotic cell lines
☒	☐ Palaeontology and archaeology
☒	☐ Animals and other organisms
☒	☐ Clinical data
☒	☐ Dual use research of concern
☒	☐ Plants

Methods

n/a	Involved in the study
☐	☒ ChIP-seq
☒	☐ Flow cytometry
☒	☐ MRI-based neuroimaging

Antibodies

Antibodies used	anti-biotin (D5A7) Rabbit mAb (Cell Signaling Technology, catalogue no. 5597S), Guinea Pig anti-Rabbit IgG secondary antibody (antibodies-online, catalogue no. ABIN101961), BG4 (scFv) was expressed as previously described (Biffi, G. et al. Nat. Chem. 5, 182–186 (2013); Hänsel-Hertsch, R. et al. Nat. Protoc. 13, 551–564 (2018)), rabbit anti-FLAG antibody (Cell Signaling Technology, catalogue no. 2368S), rabbit anti-γH2AX (Cell Signaling Technology, catalogue no. 2577), rabbit anti-PARP-1 (Cell Signaling Technology, catalogue no. 9532), rabbit anti-β-Actin (Cell Signaling Technology, catalogue no. 4970).
Validation	All commercial antibodies were validated by the manufacturers or ourselves according to a previous study. Anti-biotin (D5A7) Rabbit mAb (Cell Signaling Technology, catalogue no. 5597S) was validated by the manufacturer for Western blot and ELISA in species including human and mouse, with expected cross-reactivity across all species. Guinea Pig anti-Rabbit IgG secondary antibody (antibodies-online, catalogue no. ABIN101961) was tested for ELISA, immunohistochemistry, Western blot, CUT&RUN, and CUT&Tag in rabbit samples and preadsorbed to minimize cross-reactivity with other species. Rabbit anti-FLAG antibody (Cell Signaling Technology, catalogue no. 2368S) was validated by the manufacturer for Western Blot, immunoprecipitation, and immunofluorescence in human samples, with expected cross-reactivity across all species. Rabbit anti-γH2AX (Cell Signaling Technology, catalogue no. 2577) was validated for Western blot, immunofluorescence, and flow cytometry in species including human, mouse, rat, and monkey. Rabbit anti-PARP-1 (Cell Signaling Technology, catalogue no. 9532) was validated for Western blot, immunoprecipitation, and eCLIP in species including human, mouse, rat, and monkey. Rabbit anti-β-Actin (Cell Signaling Technology, catalogue no. 4970) was validated for Western blot, immunohistochemistry, immunofluorescence, and flow cytometry in species including human, mouse, rat, monkey, bovine, and pig, and predicted to react with hamster, chicken, dog, horse, and rabbit samples based on 100% sequence homology. We validated the binding specificity and affinity of BG4 (scFv) to folded G4 DNA oligonucleotides by ELISA, as previously described (Biffi, G. et al., Nat. Chem. 5, 182–186, 2013).

Eukaryotic cell lines

Policy information about cell lines and Sex and Gender in Research

Cell line source(s)	Human osteosarcoma U2OS cells (HTB-96) were purchased from ATCC.
Authentication	Short tandem repeat (STR) profiling was used to distinguish between individual human cell lines and rule out intra-species contamination. This was performed by the CRUK Cambridge Institute Biorepository Core Facility.
Mycoplasma contamination	Cells were confirmed mycoplasma-free by periodic tests based on RNA-capture ELISA performed by the CRUK Cambridge Institute Biorepository Core Facility.
Commonly misidentified lines (See ICLAC register)	No commonly misidentified cell lines were used.

Plants

Seed stocks

Report on the source of all seed stocks or other plant material used. If applicable, state the seed stock centre and catalogue number. If plant specimens were collected from the field, describe the collection location, date and sampling procedures.

Novel plant genotypes

Describe the methods by which all novel plant genotypes were produced. This includes those generated by transgenic approaches, gene editing, chemical/radiation-based mutagenesis and hybridization. For transgenic lines, describe the transformation method, the number of independent lines analyzed and the generation upon which experiments were performed. For gene-edited lines, describe the editor used, the endogenous sequence targeted for editing, the targeting guide RNA sequence (if applicable) and how the editor was applied.

Authentication

Describe any authentication procedures for each seed stock used or novel genotype generated. Describe any experiments used to assess the effect of a mutation and, where applicable, how potential secondary effects (e.g. second site T-DNA insertions, mosiacism, off-target gene editing) were examined.

ChIP-seq

Data deposition

☒ Confirm that both raw and final processed data have been deposited in a public database such as GEO.

☒ Confirm that you have deposited or provided access to graph files (e.g. BED files) for the called peaks.

Data access links
May remain private before publication.

As CUT&Tag approach displays substantially improved signal-to-noise and reduced backgrounds compared to CHIP-seq, so we performed G4-CUT&Tag and a derived method Chem-map to map endogenous folded G4 sites and binding sites of our small-molecule probe G4switch in the human genome. We also performed SLAM-seq experiments to profile the immediate effects of reversible G4 targeting by G4switch. The sequencing data generated in this study are available at the Gene Expression Omnibus (GEO) repository (https://www.ncbi.nlm.nih.gov/geo/) under the accession number GSE261837. The previously published GRCh38 (hg38) (https://www.ensembl.org/Homo_sapiens/Info/Index) and OQs (GSE110582) datasets were used.

Files in database submission

G4switch Chem-map data files:
GSM8152791_SLX23282_CM_U2OS_G4switch400nM_rt_2h_405nm.B1.t1.s350.cpm.bs3.bw
GSM8152792_SLX23282_CM_U2OS_G4switch400nM_rt_2h_405nm.B1.t2.s350.cpm.bs3.bw
GSM8152793_SLX23282_CM_U2OS_G4switch400nM_rt_2h_405nm.B1.t3.s350.cpm.bs3.bw
GSM8152794_SLX23282_CM_U2OS_G4switch400nM_rt_2h_405nm.B2.t1.s350.cpm.bs3.bw
GSM8152795_SLX23282_CM_U2OS_G4switch400nM_rt_2h_405nm.B2.t2.s350.cpm.bs3.bw
GSM8152796_SLX23282_CM_U2OS_G4switch400nM_rt_2h_405nm.B2.t3.s350.cpm.bs3.bw
GSM8152797_SLX23282_CM_U2OS_G4switch400nM_rt_2h_405nm.B3.t1.s350.cpm.bs3.bw
GSM8152798_SLX23282_CM_U2OS_G4switch400nM_rt_2h_405nm.B3.t2.s350.cpm.bs3.bw
GSM8152799_SLX23282_CM_U2OS_G4switch400nM_rt_2h_405nm.B3.t3.s350.cpm.bs3.bw
GSM8152800_SLX23282_CM_U2OS_G4switch400nM_rt_2h_dark.B1.t1.s350.cpm.bs3.bw
GSM8152801_SLX23282_CM_U2OS_G4switch400nM_rt_2h_dark.B1.t2.s350.cpm.bs3.bw
GSM8152802_SLX23282_CM_U2OS_G4switch400nM_rt_2h_dark.B1.t3.s350.cpm.bs3.bw
GSM8152803_SLX23282_CM_U2OS_G4switch400nM_rt_2h_dark.B2.t1.s350.cpm.bs3.bw
GSM8152804_SLX23282_CM_U2OS_G4switch400nM_rt_2h_dark.B2.t2.s350.cpm.bs3.bw
GSM8152805_SLX23282_CM_U2OS_G4switch400nM_rt_2h_dark.B2.t3.s350.cpm.bs3.bw
GSM8152806_SLX23282_CM_U2OS_G4switch400nM_rt_2h_dark.B3.t1.s350.cpm.bs3.bw
GSM8152807_SLX23282_CM_U2OS_G4switch400nM_rt_2h_dark.B3.t2.s350.cpm.bs3.bw
GSM8152808_SLX23282_CM_U2OS_G4switch400nM_rt_2h_dark.B3.t3.s350.cpm.bs3.bw
GSM8152809_SLX23282_CM_U2OS_no1stAb_405nm.B1.t1.s350.cpm.bs3.bw
GSM8152810_SLX23282_CM_U2OS_no1stAb_405nm.B2.t1.s350.cpm.bs3.bw
GSM8152811_SLX23282_CM_U2OS_no1stAb_405nm.B3.t1.s350.cpm.bs3.bw
Chem-map competition data files:
GSM8152812_SLX23283_CM_U2OS_DMSO_3h_G4switch400nM_2h_405nm.B1.t1.s350.cpm.bs3.bw
GSM8152813_SLX23283_CM_U2OS_DMSO_3h_G4switch400nM_2h_405nm.B2.t1.s350.cpm.bs3.bw
GSM8152814_SLX23283_CM_U2OS_DMSO_3h_G4switch400nM_2h_405nm.B3.t1.s350.cpm.bs3.bw
GSM8152815_SLX23283_CM_U2OS_PDS_20uM_3h_G4switch400nM_2h_405nm.B1.t1.s350.cpm.bs3.bw
GSM8152816_SLX23283_CM_U2OS_PDS_20uM_3h_G4switch400nM_2h_405nm.B2.t1.s350.cpm.bs3.bw
GSM8152817_SLX23283_CM_U2OS_PDS_20uM_3h_G4switch400nM_2h_405nm.B3.t1.s350.cpm.bs3.bw
GSM8152818_SLX23283_CM_U2OS_PDS_4uM_3h_G4switch400nM_2h_405nm.B1.t1.s350.cpm.bs3.bw
GSM8152819_SLX23283_CM_U2OS_PDS_4uM_3h_G4switch400nM_2h_405nm.B2.t1.s350.cpm.bs3.bw
GSM8152820_SLX23283_CM_U2OS_PDS_4uM_3h_G4switch400nM_2h_405nm.B3.t1.s350.cpm.bs
G4-CUT&Tag data files:
GSM8152786_SLX22701_BG4_CnT_U2OS_DMSO_noUV.B1.t1.s350.cpm.bs3.bw
GSM8152787_SLX22701_BG4_CnT_U2OS_DMSO_noUV.B1.t2.s350.cpm.bs3.bw
GSM8152788_SLX22701_BG4_CnT_U2OS_DMSO_noUV.B1.t3.s350.cpm.bs3.bw
GSM8152789_SLX22701_BG4_CnT_U2OS_DMSO_noUV.B1.t4.s350.cpm.bs3.bw
GSM8152790_SLX22701_BG4_CnT_U2OS_DMSO_noUV.B1.t5.s350.cpm.bs3.bw
SLAM-seq data files:
GSM8152766_DMSO_rep1_tcount.reduced.tsv.gz
GSM8152766_DMSO_rep1_tcount_minus.bedgraph.gz
GSM8152766_DMSO_rep1_tcount_plus.bedgraph.gz

GSM8152767_DMSO_rep2_tcount.reduced.tsv.gz
GSM8152767_DMSO_rep2_tcount_minus.bedgraph.gz
GSM8152767_DMSO_rep2_tcount_plus.bedgraph.gz
GSM8152768_DMSO_rep3_tcount.reduced.tsv.gz
GSM8152768_DMSO_rep3_tcount_minus.bedgraph.gz
GSM8152768_DMSO_rep3_tcount_plus.bedgraph.gz
GSM8152769_DMSO_rep4_tcount.reduced.tsv.gz
GSM8152769_DMSO_rep4_tcount_minus.bedgraph.gz
GSM8152769_DMSO_rep4_tcount_plus.bedgraph.gz
GSM8152770_G4switch_405nm+525nm_rep1_tcount.reduced.tsv.gz
GSM8152770_G4switch_405nm+525nm_rep1_tcount_minus.bedgraph.gz
GSM8152770_G4switch_405nm+525nm_rep1_tcount_plus.bedgraph.gz
GSM8152771_G4switch_405nm+525nm_rep2_tcount.reduced.tsv.gz
GSM8152771_G4switch_405nm+525nm_rep2_tcount_minus.bedgraph.gz
GSM8152771_G4switch_405nm+525nm_rep2_tcount_plus.bedgraph.gz
GSM8152772_G4switch_405nm+525nm_rep3_tcount.reduced.tsv.gz
GSM8152772_G4switch_405nm+525nm_rep3_tcount_minus.bedgraph.gz
GSM8152772_G4switch_405nm+525nm_rep3_tcount_plus.bedgraph.gz
GSM8152773_G4switch_405nm+525nm_rep4_tcount.reduced.tsv.gz
GSM8152773_G4switch_405nm+525nm_rep4_tcount_minus.bedgraph.gz
GSM8152773_G4switch_405nm+525nm_rep4_tcount_plus.bedgraph.gz
GSM8152774_G4switch_405nm_rep1_tcount.reduced.tsv.gz
GSM8152774_G4switch_405nm_rep1_tcount_minus.bedgraph.gz
GSM8152774_G4switch_405nm_rep1_tcount_plus.bedgraph.gz
GSM8152775_G4switch_405nm_rep2_tcount.reduced.tsv.gz
GSM8152775_G4switch_405nm_rep2_tcount_minus.bedgraph.gz
GSM8152775_G4switch_405nm_rep2_tcount_plus.bedgraph.gz
GSM8152776_G4switch_405nm_rep3_tcount.reduced.tsv.gz
GSM8152776_G4switch_405nm_rep3_tcount_minus.bedgraph.gz
GSM8152776_G4switch_405nm_rep3_tcount_plus.bedgraph.gz
GSM8152777_G4switch_405nm_rep4_tcount.reduced.tsv.gz
GSM8152777_G4switch_405nm_rep4_tcount_minus.bedgraph.gz
GSM8152777_G4switch_405nm_rep4_tcount_plus.bedgraph.gz
GSM8152778_G4switch_525nm_rep1_tcount.reduced.tsv.gz
GSM8152778_G4switch_525nm_rep1_tcount_minus.bedgraph.gz
GSM8152778_G4switch_525nm_rep1_tcount_plus.bedgraph.gz
GSM8152779_G4switch_525nm_rep2_tcount.reduced.tsv.gz
GSM8152779_G4switch_525nm_rep2_tcount_minus.bedgraph.gz
GSM8152779_G4switch_525nm_rep2_tcount_plus.bedgraph.gz
GSM8152780_G4switch_525nm_rep3_tcount.reduced.tsv.gz
GSM8152780_G4switch_525nm_rep3_tcount_minus.bedgraph.gz
GSM8152780_G4switch_525nm_rep3_tcount_plus.bedgraph.gz
GSM8152781_G4switch_525nm_rep4_tcount.reduced.tsv.gz
GSM8152781_G4switch_525nm_rep4_tcount_minus.bedgraph.gz
GSM8152781_G4switch_525nm_rep4_tcount_plus.bedgraph.gz
GSM8152782_G4switch_dark_rep1_tcount.reduced.tsv.gz
GSM8152782_G4switch_dark_rep1_tcount_minus.bedgraph.gz
GSM8152782_G4switch_dark_rep1_tcount_plus.bedgraph.gz
GSM8152783_G4switch_dark_rep2_tcount.reduced.tsv.gz
GSM8152783_G4switch_dark_rep2_tcount_minus.bedgraph.gz
GSM8152783_G4switch_dark_rep2_tcount_plus.bedgraph.gz
GSM8152784_G4switch_dark_rep3_tcount.reduced.tsv.gz
GSM8152784_G4switch_dark_rep3_tcount_minus.bedgraph.gz
GSM8152784_G4switch_dark_rep3_tcount_plus.bedgraph.gz
GSM8152785_G4switch_dark_rep4_tcount.reduced.tsv.gz
GSM8152785_G4switch_dark_rep4_tcount_minus.bedgraph.gz
GSM8152785_G4switch_dark_rep4_tcount_plus.bedgraph.gz

Genome browser session
(e.g. UCSC)

no longer applicable

Methodology

Replicates

The standard Chem-map experiments were performed in three independent biological replicates each with three technical replicates (consensus peaks are defined by the overlap of the technical replicates for each biological and 2 out of 3 biological replicates for each feature).
The Chem-map competition experiments were performed in three independent biological replicates (consensus peaks are defined by the overlap of 2 out of 3 biological replicates for each feature).
The G4-CUT&Tag experiments were performed in five replicates (consensus peaks are defined by the overlap of the 3 out of 5 replicates for each feature).
The SLAM-seq experiments were performed in four independent biological replicates.

Sequencing depth

Numbers of paired-end sequencing reads for Chem-map (61 bp read length) and G4-CUT&Tag (38 bp read length), and single-end reads for SLAM-seq (75 bp read length) are shown below in the unit of million (M):
G4switch Chem-map:
SLX23282_CM_U2OS_G4switch400nM_rt_2h_405nm.B1.t1, 27.7 M

SLX23282_CM_U2OS_G4switch400nM_rt_2h_405nm.B1.t2, 25.1 M
SLX23282_CM_U2OS_G4switch400nM_rt_2h_405nm.B1.t3, 27.4 M
SLX23282_CM_U2OS_G4switch400nM_rt_2h_405nm.B2.t1, 41.3 M
SLX23282_CM_U2OS_G4switch400nM_rt_2h_405nm.B2.t2, 23.6 M
SLX23282_CM_U2OS_G4switch400nM_rt_2h_405nm.B2.t3, 29.2 M
SLX23282_CM_U2OS_G4switch400nM_rt_2h_405nm.B3.t1, 32.9 M
SLX23282_CM_U2OS_G4switch400nM_rt_2h_405nm.B3.t2, 17.5 M
SLX23282_CM_U2OS_G4switch400nM_rt_2h_405nm.B3.t3, 33.7 M
SLX23282_CM_U2OS_G4switch400nM_rt_2h_dark.B1.t1, 26.8 M
SLX23282_CM_U2OS_G4switch400nM_rt_2h_dark.B1.t2, 33.3 M
SLX23282_CM_U2OS_G4switch400nM_rt_2h_dark.B1.t3, 22.8 M
SLX23282_CM_U2OS_G4switch400nM_rt_2h_dark.B2.t1, 26.5 M
SLX23282_CM_U2OS_G4switch400nM_rt_2h_dark.B2.t2, 33.8 M
SLX23282_CM_U2OS_G4switch400nM_rt_2h_dark.B2.t3, 27.5 M
SLX23282_CM_U2OS_G4switch400nM_rt_2h_dark.B3.t1, 21.0 M
SLX23282_CM_U2OS_G4switch400nM_rt_2h_dark.B3.t2, 35.0 M
SLX23282_CM_U2OS_G4switch400nM_rt_2h_dark.B3.t3, 31.0 M
SLX23282_CM_U2OS_no1stAb_405nm.B1.t1, 1.8 M
SLX23282_CM_U2OS_no1stAb_405nm.B2.t1, 0.9 M
SLX23282_CM_U2OS_no1stAb_405nm.B3.t1, 0.2 M
Chem-map competition:
SLX23283_CM_U2OS_DMSO_3h_G4switch400nM_2h_405nm.B1.t1, 24.3 M
SLX23283_CM_U2OS_DMSO_3h_G4switch400nM_2h_405nm.B2.t1, 29.9 M
SLX23283_CM_U2OS_DMSO_3h_G4switch400nM_2h_405nm.B3.t1, 38.3 M
SLX23283_CM_U2OS_PDS_20uM_3h_G4switch400nM_2h_405nm.B1.t1, 29.5 M
SLX23283_CM_U2OS_PDS_20uM_3h_G4switch400nM_2h_405nm.B2.t1, 25.0 M
SLX23283_CM_U2OS_PDS_20uM_3h_G4switch400nM_2h_405nm.B3.t1, 31.8 M
SLX23283_CM_U2OS_PDS_4uM_3h_G4switch400nM_2h_405nm.B1.t1, 21.9 M
SLX23283_CM_U2OS_PDS_4uM_3h_G4switch400nM_2h_405nm.B2.t1, 28.9 M
SLX23283_CM_U2OS_PDS_4uM_3h_G4switch400nM_2h_405nm.B3.t1, 26.0 M
G4-CUT&Tag:
SLX22701_BG4_CnT_U2OS_DMSO_noUV.B1.t1, 16.5 M
SLX22701_BG4_CnT_U2OS_DMSO_noUV.B1.t2, 13.2 M
SLX22701_BG4_CnT_U2OS_DMSO_noUV.B1.t3, 10.9 M
SLX22701_BG4_CnT_U2OS_DMSO_noUV.B1.t4, 12.0 M
SLX22701_BG4_CnT_U2OS_DMSO_noUV.B1.t5, 23.6 M
SLAM-seq:
DMSO_dark_rep1, 20.0 M
DMSO_dark_rep2, 20.4 M
DMSO_dark_rep3, 20.3 M
DMSO_dark_rep4, 20.7 M
G4switch_dark_rep1, 21.7 M
G4switch_dark_rep2, 20.5 M
G4switch_dark_rep3, 19.7 M
G4switch_dark_rep4, 21.8 M
G4switch_405nm_rep1, 22.0 M
G4switch_405nm_rep2, 21.7 M
G4switch_405nm_rep3, 21.0 M
G4switch_405nm_rep4, 21.3 M
G4switch_405+525nm_rep1, 19.8 M
G4switch_405+525nm_rep2, 21.1 M
G4switch_405+525nm_rep3, 21.3 M
G4switch_405+525nm_rep4, 22.2 M
G4switch_525nm_rep1, 19.7 M
G4switch_525nm_rep2, 20.2 M
G4switch_525nm_rep3, 22.4 M
G4switch_525nm_rep4, 21.6 M

Antibodies

For each replicate, we employed:
3.4 µl of anti-biotin (D5A7) Rabbit mAb in ~1:30 dilution against G4switch-biotin (Cell Signaling Technology, catalogue no. 5597S);
1 µl of Guinea Pig anti-Rabbit IgG secondary antibody (antibodies-online, catalogue no. ABIN101961) in 1:100 dilution against anti-biotin (D5A7) Rabbit mAb;
~600 nM BG4 (scFv) in 50 µl assay buffer against endogenous folded G4 sites;
2 µl of rabbit anti-FLAG antibody (Cell Signaling Technology, catalogue no. 2368S) in 1:25 dilution against BG4.

Peak calling parameters

Peak calling parameters for Chem-map and G4-CUT&Tag experiments: ~/applications/SEACR_1.3.sh *bedgraph 0.01 non stringent.
'Slamdunk all' command parameters for SLAM-seq read mapping: : -t 5 -5 12 -n 100 -m -mv 0.2 -c 2 -rl 100

Data quality

Individual peak numbers for q-value <0.01:
for Chem-map:
SLX23282_CM_U2OS_G4switch400nM_rt_2h_405nm.PCR-10.B1.t1.merged.min0.bed 15420
SLX23282_CM_U2OS_G4switch400nM_rt_2h_405nm.PCR-10.B1.t2.merged.min0.bed 15075
SLX23282_CM_U2OS_G4switch400nM_rt_2h_405nm.PCR-10.B1.t3.merged.min0.bed 15556
SLX23282_CM_U2OS_G4switch400nM_rt_2h_405nm.PCR-10.B2.t1.merged.min0.bed 18968
SLX23282_CM_U2OS_G4switch400nM_rt_2h_405nm.PCR-10.B2.t2.merged.min0.bed 16544
SLX23282_CM_U2OS_G4switch400nM_rt_2h_405nm.PCR-10.B2.t3.merged.min0.bed 16748

SLX23282_CM_U2OS_G4switch400nM_rt_2h_405nm.PCR-10.B3.t1.merged.min0.bed, 15586
SLX23282_CM_U2OS_G4switch400nM_rt_2h_405nm.PCR-10.B3.t2.merged.min0.bed, 14815
SLX23282_CM_U2OS_G4switch400nM_rt_2h_405nm.PCR-10.B3.t3.merged.min0.bed, 20182
SLX23282_CM_U2OS_G4switch400nM_rt_2h_dark.PCR-10.B1.t1.merged.min0.bed, 10084
SLX23282_CM_U2OS_G4switch400nM_rt_2h_dark.PCR-10.B1.t2.merged.min0.bed, 9214
SLX23282_CM_U2OS_G4switch400nM_rt_2h_dark.PCR-10.B1.t3.merged.min0.bed, 7790
SLX23282_CM_U2OS_G4switch400nM_rt_2h_dark.PCR-10.B2.t1.merged.min0.bed, 6056
SLX23282_CM_U2OS_G4switch400nM_rt_2h_dark.PCR-10.B2.t2.merged.min0.bed, 6505
SLX23282_CM_U2OS_G4switch400nM_rt_2h_dark.PCR-10.B2.t3.merged.min0.bed, 5471
SLX23282_CM_U2OS_G4switch400nM_rt_2h_dark.PCR-10.B3.t1.merged.min0.bed, 4152
SLX23282_CM_U2OS_G4switch400nM_rt_2h_dark.PCR-10.B3.t2.merged.min0.bed, 7497
SLX23282_CM_U2OS_G4switch400nM_rt_2h_dark.PCR-10.B3.t3.merged.min0.bed, 5560
SLX23282_CM_U2OS_no1stAb_405nm.PCR-10.B1.t1.merged.min0.bed, 2692
SLX23282_CM_U2OS_no1stAb_405nm.PCR-10.B2.t1.merged.min0.bed, 1193
SLX23282_CM_U2OS_no1stAb_405nm.PCR-10.B3.t1.merged.min0.bed, 271
for G4-CUT&Tag:
SLX22701_bg4_CnT_U2OS_DMSO_noUV_BG4.PCR-10.B1.t1.sortName.1000.clean.fragments.bedgraph.0.01fdr.stringent.bed, 19866
SLX22701_bg4_CnT_U2OS_DMSO_noUV_BG4.PCR-10.B1.t2.sortName.1000.clean.fragments.bedgraph.0.01fdr.stringent.bed, 17752
SLX22701_bg4_CnT_U2OS_DMSO_noUV_BG4.PCR-10.B1.t3.sortName.1000.clean.fragments.bedgraph.0.01fdr.stringent.bed, 15372
SLX22701_bg4_CnT_U2OS_DMSO_noUV_BG4.PCR-10.B1.t4.sortName.1000.clean.fragments.bedgraph.0.01fdr.stringent.bed, 16377
SLX22701_bg4_CnT_U2OS_DMSO_noUV_BG4.PCR-10.B1.t5.sortName.1000.clean.fragments.bedgraph.0.01fdr.stringent.bed, 24248

Software

The following tools were used to analyse Chem-map, SLAM-seq, and G4-CUT&Tag data: demuxIllumina (version 3.0.9), FastQC (version 0.11.8), MultiQC (version 1.11), cutadapt (version 1.18), BWA (version 0.7.17-r1188), Picard (version 2.20.3), Seacr (version 1.3), EnhancedVolcano (v.1.16.0), deepTools (version 2.0), DiffBind (version 3.10.1), Gencode v37, SlamDunk package (v.0.3.4) and DESeq2 (version 1.2.10).
