# Supplementary figures and images for: Optical control of gene expression using a DNA G-quadruplex targeting reversible photoswitch
Source: Nat Chem. 2025 Apr 3;17(6):875–82. doi: 10.1038/s41557-025-01792-1 (PMC12141046; doi:10.1038/s41557-025-01792-1)

**Fig. 5** Unprocessed image of crystal violet staining

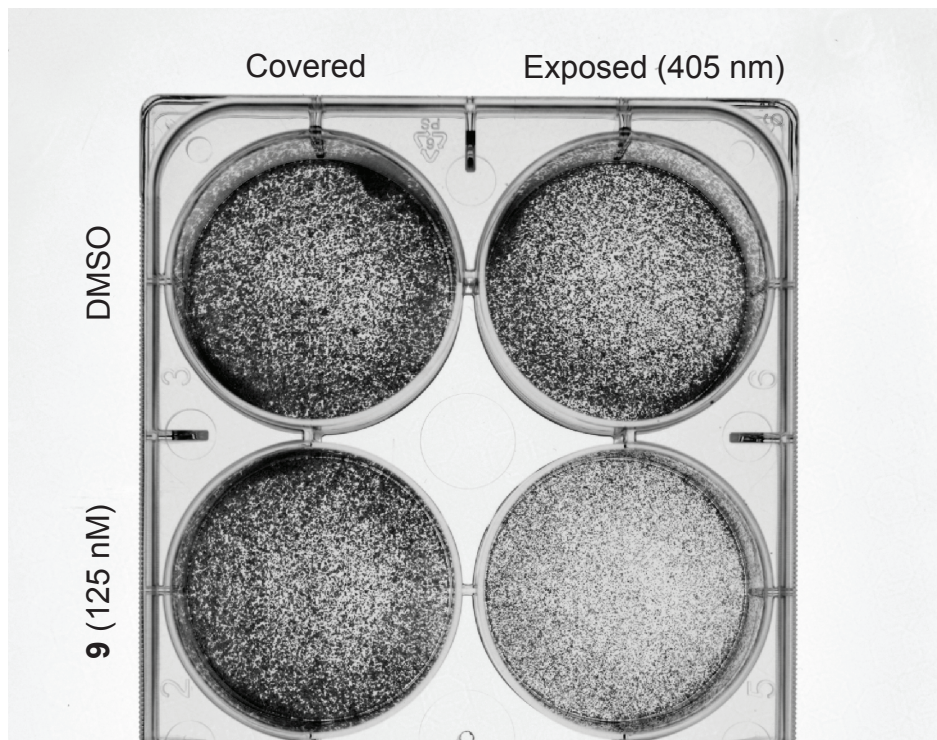

Supplement: Supplementary file 7 — Unprocessed image. [file 41557_2025_1792_MOESM7_ESM.pdf]
