# Supplementary material for: Optical control of gene expression using a DNA G-quadruplex targeting reversible photoswitch
Source: Nat Chem. 2025 Apr 3;17(6):875–82. doi: 10.1038/s41557-025-01792-1 (PMC12141046; doi:10.1038/s41557-025-01792-1)

Extended Data Fig. 4f Unprocessed image of γH2AX Western blot analyzed using a Jess automated capillary Western system from Bio-Techne.

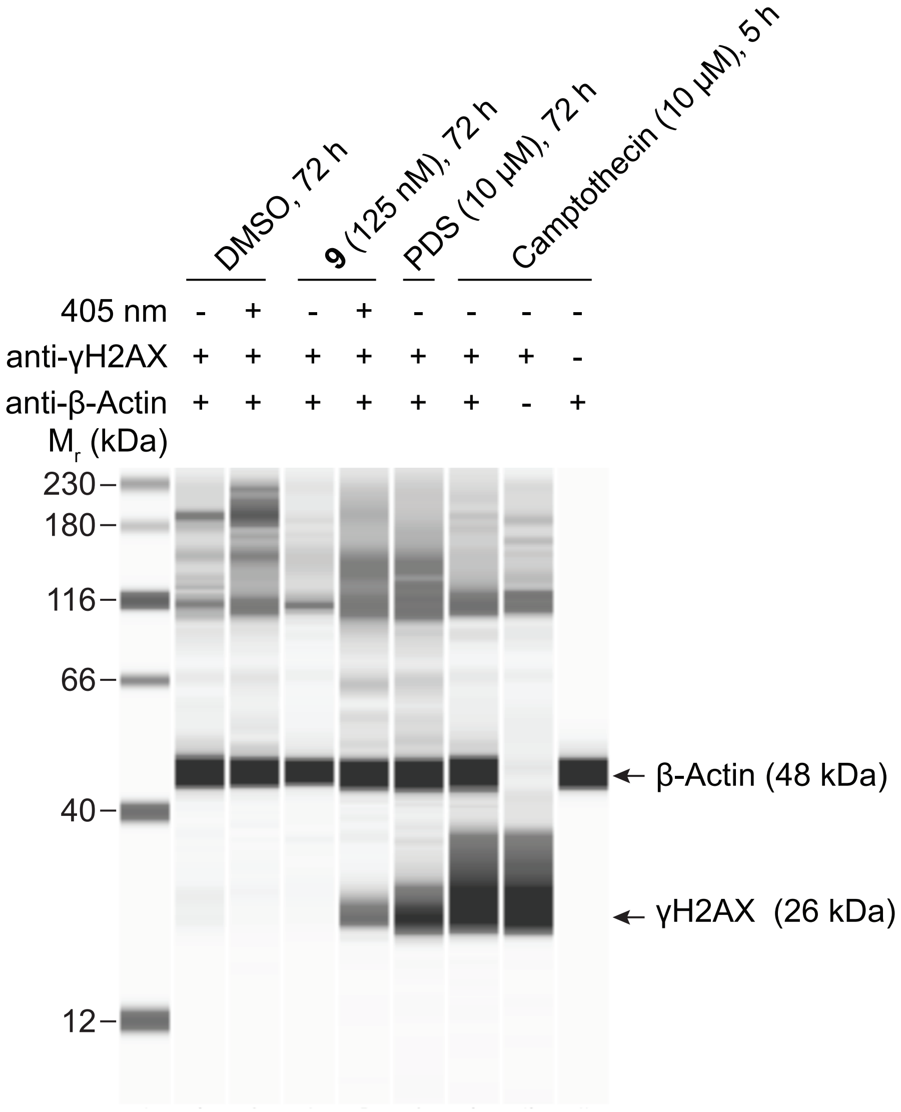

Supplement: Supplementary file 13 — Unprocessed western blots. [file 41557_2025_1792_MOESM13_ESM.pdf]
